# Deuterium isotope effects in drug pharmacokinetics II: Substrate-dependence of the reaction mechanism influences outcome for cytochrome P450 cleared drugs

**DOI:** 10.1371/journal.pone.0206279

**Published:** 2018-11-14

**Authors:** Hao Sun, David W. Piotrowski, Suvi T. M. Orr, Joseph S. Warmus, Angela C. Wolford, Steven B. Coffey, Kentaro Futatsugi, Yinsheng Zhang, Alfin D. N. Vaz

**Affiliations:** Medicine Design, Pfizer Global Research and Development, Groton, Connecticut, United States of America; University of Kentucky, UNITED STATES

## Abstract

Two chemotypes were examined in vitro with CYPs 3A4 and 2C19 by molecular docking, metabolic profiles, and intrinsic clearance deuterium isotope effects with specifically deuterated form to assess the potential for enhancement of pharmacokinetic parameters. The results show the complexity of deuteration as an approach for pharmacokinetic enhancement when CYP enzymes are involved in metabolic clearance. With CYP3A4 the rate limiting step was chemotype-dependent. With one chemotype no intrinsic clearance deuterium isotope effect was observed with any deuterated form, whereas with the other chemotype the rate limiting step was isotopically sensitive, and the magnitude of the intrinsic clearance isotope effect was dependent on the position(s) and extent of deuteration. Molecular docking and metabolic profiles aided in identifying sites for deuteration and predicted the possibility for metabolic switching. However, the potential for an isotope effect on the intrinsic clearance cannot be predicted and must be established by examining select deuterated versions of the chemotypes. The results show how in a deuteration strategy molecular docking, in-vitro metabolic profiles, and intrinsic clearance assessments with select deuterated versions of new chemical entities can be applied to determine the potential for pharmacokinetic enhancement in a discovery setting. They also help explain the substantial failures reported in the literature of deuterated versions of drugs to elicit a systemic enhancement on pharmacokinetic parameters.

## Introduction

Because of the potential to enhance pharmacokinetic properties or decrease toxicity by virtue of a kinetic deuterium isotope effect, the replacement of hydrogen by deuterium at non-exchangeable carbon-hydrogen bonds of drug molecules has received extensive attention as indicated by an exponential increase over the past decade in patent applications for deuterated versions of existing pharmaceuticals and new chemical entities [1,2]. As reported previously by us for aldehyde oxidase-cleared drugs, successful application of a deuteration strategy requires a clear understanding of the metabolic and systemic clearance mechanisms, and species differences in metabolic pathways [3].

Cytochrome P450 enzymes (CYP) are responsible for over 90% of all metabolic clearance of drugs and xenobiotics, and three quarters of these reactions are attributable to five CYP isoforms (1A2, 2C9, 2C19, 2D6, and 3A4) with CYP3A4 contributing approximately 27% to the metabolism of all marketed drugs [4]. Thus, any deuteration strategy must consider the complex reaction mechanisms of these enzymes that can confound a deuteration strategy leading to a failure to achieve significant systemic pharmacokinetic gain even when metabolism by these enzymes may be rate-limiting in systemic clearance [5–8]. Examples of such mechanistic complexity include: a) Differences in reaction mechanisms of C-H bond cleavages such as the N- and O- dealkylation reactions, where single electron transfer and hydrogen atom abstraction mechanisms can have substantial differences in the magnitude of their intrinsic deuterium isotope effect [9,10]; b) Deuterium-induced metabolic switching to proximal or distal non-deuterated sites [11,12] which is possibly due to multiple binding orientations of a molecule within an active site, or freedom for a bound molecule to “tumble” within an active site because of the large active site cavity of some CYP enzymes, such that oxidation at a non-deuterated site compensates for decreased metabolism at the deuterated site resulting in loss of an isotope effect on the intrinsic clearance and a redistribution of the relative abundance of metabolites; and c) A rate limiting release of product resulting in masking of the intrinsic deuterium isotope effect (^*H*^*k/*^*D*^*k*) on the intrinsic clearance (^*H*^*V*_*m*_*/K*_*m*_ */* ^*D*^*V*_*m*_*/K*_*m*_) [13,14].

**Fig 1:**
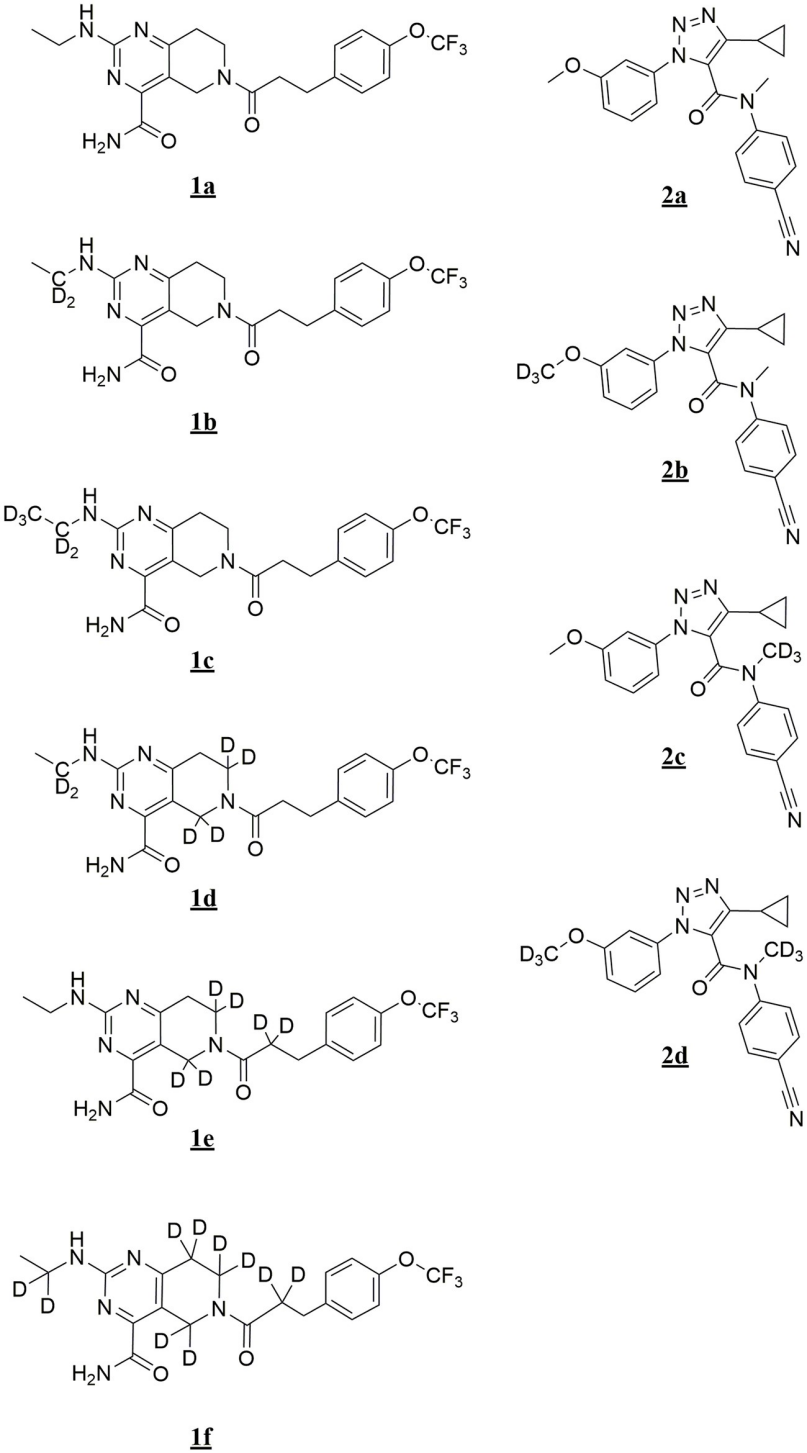
Structures of chemotypes 1a and 2a and their respective deuterated forms examined in this study.

**Fig 2:**
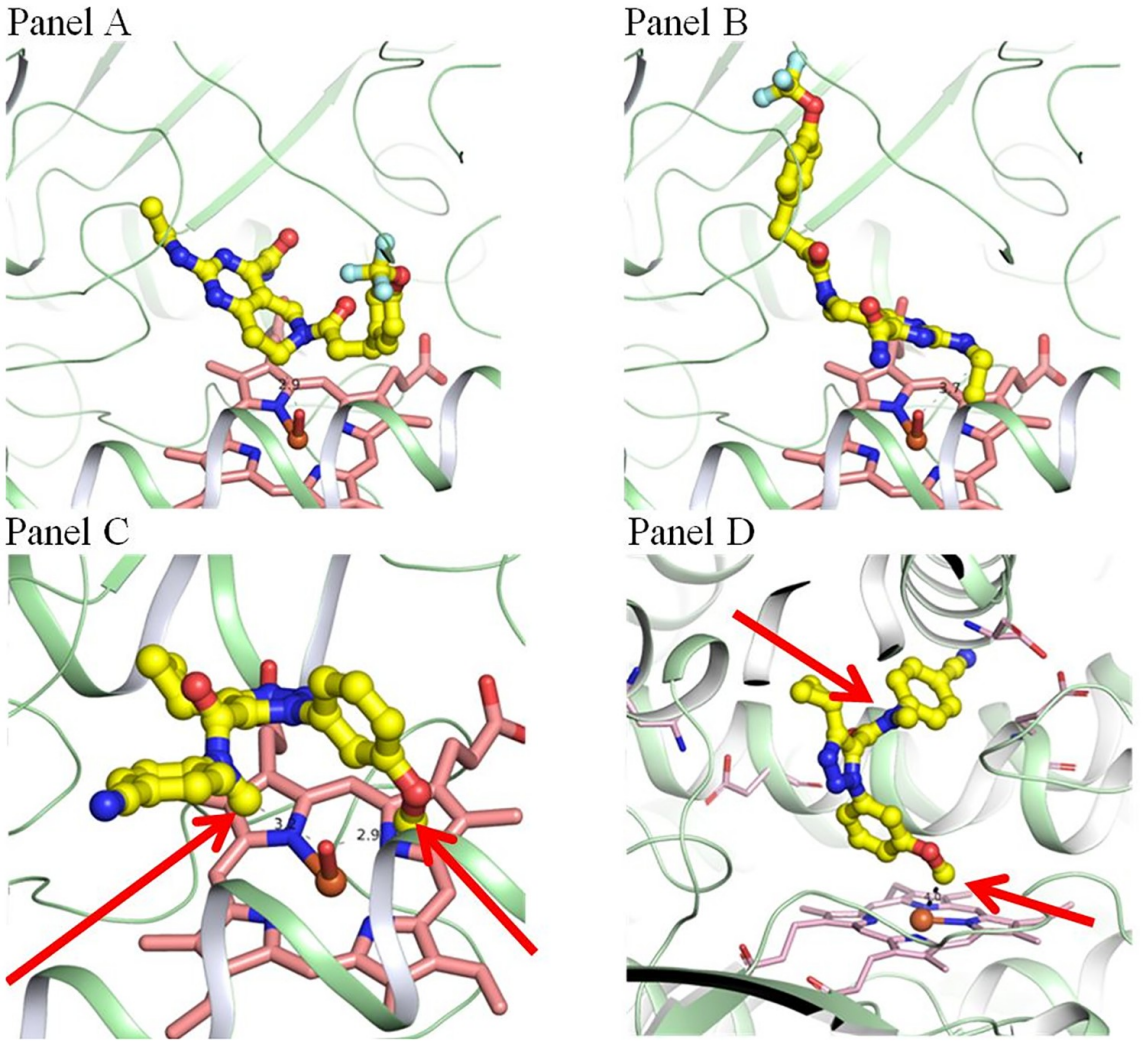
Snapshots of docked 1a to the active site of CYP3A4 (Panels A and B), and 2a to the active sites of CYP 3A4 and CYP 2C19 (Panels C and D respectively). Panels A and B show two discrete binding modes for compound **1a** in the active site of CYP3A4 with either the pyrimidino-piperidine ring (Panel A), or the N-ethyl (Panel B) in proximity to the putative heme iron-oxo species. The arrows in Panels C and D show the N- and O-methyl groups of **2a**. Panel C shows the binding of **2a** to the active site of CYP3A4 with both methyl groups in similar proximity to the putative oxidant in CYP3A4, and Panel D shows the preferred binding mode with CYP2C19, where the O-methyl group of **2a** is in proximity to the putative oxidant.

In this study we examined two structurally distinct chemo-types (Fig 1, **1a** and **2a**) where in-vitro clearance predictions with hepatic microsomes and hepatocytes suggested a blood flow-limited, CYP-mediated oxidative metabolic clearance. Using virtual molecular docking with CYPs 3A4 and 2C19, metabolic profiles and intrinsic clearance isotope effects with human liver microsomes and recombinant CYPs 3A4 and 2C19 with deuterated versions of **1a** and **2a**, we demonstrate the mechanistic complexities of CYP-catalyzed reactions where the rate limiting step may be determined by the substrate under consideration. The two chemotypes examined also provide an understanding of how to address a deuteration strategy for new chemical entities, and helps explain the numerous reports where deuteration has been largely ineffective in substantially altering the in-vivo pharmacokinetics of some CYP-cleared compounds [5–8].

## Materials and methods

The synthesis and characterization of chemotypes **1a** and **2a** have been previously reported [15,16]. Synthesis procedures for **1a** and analytical data for deuterated analogs of **1a** and **2a** are presented in Supporting information (S1 File). The identities of primary metabolites from **1a** and **2a** were established from their mass spectral fragment patterns and are presented in Supporting information (S2 File).

### Molecular docking

Structures of **1a** and **2a** were constructed using ChemBioOffice (PerkinElmer Inc. Waltham, MA) and stored in SD format. These structures were then modified with a customized script written in Python: the explicit hydrogen atoms were added, formal charge was calculated, and the structures were transformed into PDB format, with the integration of the OEChem Toolkit (OpenEye Scientific Software Inc., Santa Fe, NM). The derived molecular structures were further optimized with a DFT/B3LYP (Becke three-parameter Lee-Yang-Parr) approach using a 6-31G** basis set in Gaussian 09 (Gaussian, Inc., Wallingford, CT). The energetically minimized structures of **1a** and **2a** were then modified by AutoDockTools (The Scripps Research Institute, La Jolla, CA) with flexible torsions defined and Gasteiger atomic charges assigned, as the final ligand input files for docking. To prepare the protein templates, three-dimensional coordinates of CYP3A4 and CYP2C19 structures were collected from both Protein Data Bank and Pfizer’s Protein Structure Database. The protein templates were selected and customized specifically for **1a** and **2a** with previous findings [17]. Specifically, for CYP3A4, the core template was 3NXU, a crystal structure determined at resolution of 2.0 Å with the inhibitor ritonavir bound [18]; for CYP2C19, 4GQS, a crystal structure determined at resolution of 2.9 Å complexed with the inhibitor (2-methyl-1-benzofuran-3-yl)-(4-hydroxy-3,5-dimethylphenyl)methanone was used as the template [19]. These templates were further modified by AutoDockTools to add polar hydrogen atoms, Kollman partial charges, and solvation parameters. The partial charge of the iron (Fe) was assigned as 0.262 and the proximal oxygen (O) as -0.342, with the compound I Fe-O length assigned as 1.6 Å, according to previously quantum mechanically derived heme parameters [20]. The active site space of CYP3A4 and CYP2C19 was defined by AutoGrid 4.0 (The Scripps Research Institute), which pre-calculates the van der Waals, hydrogen bonding, electrostatics, torsional, and solvation interactions between protein and studied compounds. Docking procedures were accomplished with AutoDock 4.0 (The Scripps Research Institute) on Pfizer’s high performance computing Linux clusters. The globally optimized conformation and orientation of compound **1a** and **2a** were searched using a Lamarckian generic algorithm, a hybrid of generic algorithms and an adaptive local search method. The derived 100 docking poses for each compound were clustered according to RMSD (root-mean-square deviation). The binding poses with the lowest binding energies and within 5Å to the heme iron-oxo were automatically chosen by customized scripts for further analysis, and visualized using PyMOL (Schrödinger, LLC, New York, NY).

### Reactions with human liver microsomes and recombinant CYP enzymes for the assessment of metabolic profiles, relative CYP isoform activities, and intrinsic clearance isotope effects

Microsomal and recombinant CYP reactions were conducted at 37 °C in final volumes of 1.0 mL (for first order substrate depletion rate constant assessment at substrate concentrations of 1.0 μM), and 2.0 mL (for metabolic profiles at substrate concentrations of 10 μM). Each reaction contained 100 mM potassium phosphate buffer pH 7.4, either 0.5 mg/mL human liver microsomal protein or 10 pmol/mL rCYP isoform co-expressed with cytochrome P450 oxido-reductase in insect cell membranes. Reactions were initiated by the addition of 3.0 mM NADPH or an NADPH regenerating system (0.3mM NADP^+^, 1 mM isocitrate, 0.5 mM MgCl_2_ and 1.0 unit isocitrate dehydrogenase).

For metabolic profiles, reactions were incubated for 30 minutes at 37 °C then quenched by adding 5.0 mL of acetonitrile. After mixing, the samples were centrifuged at 1800 x g for 20 minutes and the supernatants were decanted and dried at room temperature under reduced pressure in a vacuum centrifuge. The residues were re-suspended in 200 μL of acetonitrile:DMSO:water (5:20:75), centrifuged as above to remove insoluble material and a 50 to 100 μL aliquot of the supernatant was analyzed by LC/MS as described below.

For relative activities of CYP isoforms and intrinsic clearance, substrate depletion at 1.0 μM was the method of choice for assessment of the depletion rate constants as each substrate produced multiple metabolites, and an estimation of Km for substrates (**1a** and **2a**) by the substrate depletion method showed that their respective Km’s were greater than 5 μM [21,22]. Relative CYP activities were determined from the ratio of the depletion rate constant for each isoform relative to that for CYP3A4. Intrinsic clearance isotope effects were determined from competitive reactions using 1:1 mixtures of the protio and appropriate deuterio form of the substrate at 0.5 μM each. Eight 100 μL aliquots each were removed over a period of 70 to 90 minutes and added to 100 μL of a 0.1 μM solution of an internal standard in methanol to quench the enzymatic reaction. The samples were filtered through a high protein-binding capacity filter membrane in a 96-well format. The filtrates were evaporated in a vacuum centrifuge to near dryness, diluted with 125 μL of water, and a 99 μL aliquot was analyzed by reversed phase chromatography/mass spectrometry using selected reaction monitoring. Rate constants were determined from the semi-logarithmic plots of the time versus ratio of the area under the peak for specific transitions of the various substrates and the internal standard. The intrinsic clearance isotope effect was determined from the ratio of the rate constants for the protio- and respective deuterio- forms.

### LC-MS methods

An integrated Thermo-Finnigan LC/MS system consisting of a Surveyor Autosampler, LC pump, diode array detector and either an Orbitrap or LCQ mass spectrometer auto-tuned with the protio-form of the compound of interest were used in all analytical work. Two chromatographic conditions were used for analysis. For rate measurements, a steep linear gradient from 30 to 95% acetonitrile was used with a Phenomenex Luna C18, 5 μm 2 x 50 mm column. For the identification of metabolites, a shallow gradient from 5 to 95% acetonitrile at a linear rate of 2.25% per minute was used with a Phenomenex Luna C-18, 3 μm, 4.6 x 150 mm column. The gradients used are shown in Tables 1 and 2.

**Table 1:** Steep gradient for LC/MS analysis.

Time (min)	0.1% formic acid in water	Acetonitrile	Flow rate (μL/min)
0	70	30	500
2.0	70	30	500
5.0	5	95	500
6.0	5	95	500
6.5	95	5	500
8.0	95	5	500

**Table 2:** Shallow gradient for LC/MS analysis.

Time (min)	0.1% formic acid in water	Acetonitrile	Flow rate (μL/min)
0	95	5	500
5	95	5	500
45	5	95	500
50	5	95	500
52	95	5	500
60	95	5	500

Metabolites were identified by standard techniques that include: extraction of ion masses from the total ion current corresponding to known metabolic transformations; identifying drug-derived substances by extracting ion masses from the MS2 and MS3 ion chromatograms that are common to the parents in their MS2 and MS3 spectra; and, examining fragment patterns of ions in the total ion current spectrum to determine if they are drug-related, in regions where UV (250–400 nm) absorbing peaks occurred.

## Results

### Molecular modeling

Molecular docking studies of compound **1a** with CYP3A4 showed two energetically favored binding clusters (clustered at RMSD 2.0 Å) where either the pyrimidino-piperidine ring (Fig 2 Panel A) or the terminal *N*-ethyl moiety (Fig 2 Panel B) of compound **1a** are in proximity to the heme iron of CYP3A4 for aliphatic hydroxylations at the pyrimidino-piperidine ring and *N*-deethylation reactions, respectively. Other binding clusters that were also energetically favored (1–2 kcal/mol within the lowest-energy binding cluster) but not in an orientation for a typical P450-catalyzed reaction, were not included for analysis, an approach we previously reported [23–27]. For example, the binding pose with the trifluoromethyl moiety of **1a** closest to the heme may also be energetically favored, but no common P450-catalyzed reactions are expected. For **1a**, importantly, the three saturated carbon atoms on the piperidine ring are at a comparable distance to the putative CYP heme iron oxo species (3–4 Å, only one representative pose from each binding cluster was selected for Fig 2). These two molecular orientations of **1a** within the active site allow for either pyrimidino-piperidine ring hydroxylations or *N*-deethylation, predicting these to be major metabolic products from **1a**. Due to side chain interactions within the active site, these binding modes are discreet and cannot interconvert by “tumbling” of **1a** within the active site. Thus, any shift from *N*-deethylation to pyrimidino-piperidine ring hydroxylation requires substrate dissociation from the enzyme and appropriate rebinding at the heme center.

With **2a**, molecular docking studies showed that both N-methyl and O-methyl moieties of compound **2a** equally access the heme iron-oxo of CYP3A4 in an energetically favored orientation (Fig 2 Panel C), with the distance to the iron-oxo of 3.2 and 2.9Å, respectively. Anionic and polar active site residues including E308 and S312 of helix I, and Q484 of the C-terminal, together with other hydrophobic residues especially from the B-C and K-β loops, might play important roles to juxtapose the N-methyl and O-methyl moieties of compound **2a** in proximity to the heme iron for catalysis, and indicates that the hydrogen atoms from either methyl groups have an equal access to the active oxidant of CYP3A4. Thus, the ratio of products (N-demethylation vs. O-demethylation) is more likely determined by the intrinsic reactivity and reaction mechanism of these two types of methyl groups (electron transfer/proton loss for N-demethylation or hydrogen atom abstraction for O-demethylation). The proximity of both methyl groups to the active oxidant of CYP3A4 predicts metabolic switching. Molecular modeling of compound **2a** with CYP2C19 presents a different picture. Binding of **2a** to CYP2C19 within its active site space is similarly confined by the I helix, F-G, B-C, and K-β loops, but the binding pocket of CYP2C19 is much more restricted than that of CYP3A4, preventing both methyl groups from positioning at equal distances to the active oxidant of CYP2C19. The most favored binding pose for compound **2a** was with the O-methyl moiety over the heme (3.0Å, Fig 2 Panel D). This was mainly driven by some hydrophobic interactions including those between F100 of the B-C loop, F476 of the C-terminal and the pyrazole and cyclopropyl moieties of compound **2a**. This preferred binding mode predicts O-demethylation to be the primary route of metabolism of this substrate with CYP2C19 and if the rate limiting step for this substrate is hydrogen atom abstraction, an isotope effect should be expected primarily when the O-methyl group is deuterated (**2b** and **2d**) and little to no effect when only the N-methyl group is deuterated (**2c**).

### Identity of metabolites from 1a and 2a

**Fig 3:**
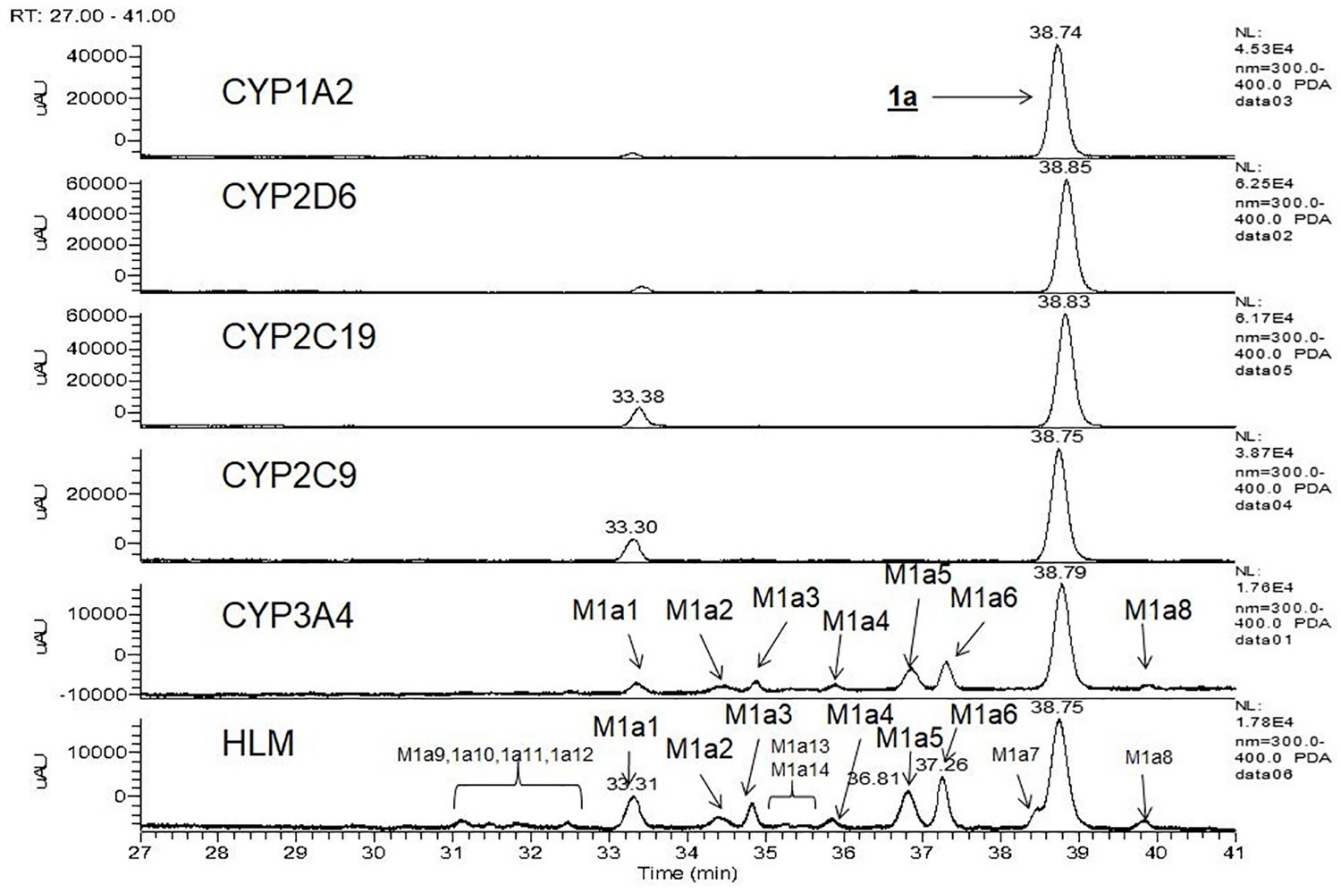
UV (300–400 nm) chromatograms for the metabolism of 1a in human liver microsomes and recombinant cytochromes 3A4, 2C9, 2C19, 2D6, and 1A2.

**Fig 4:**
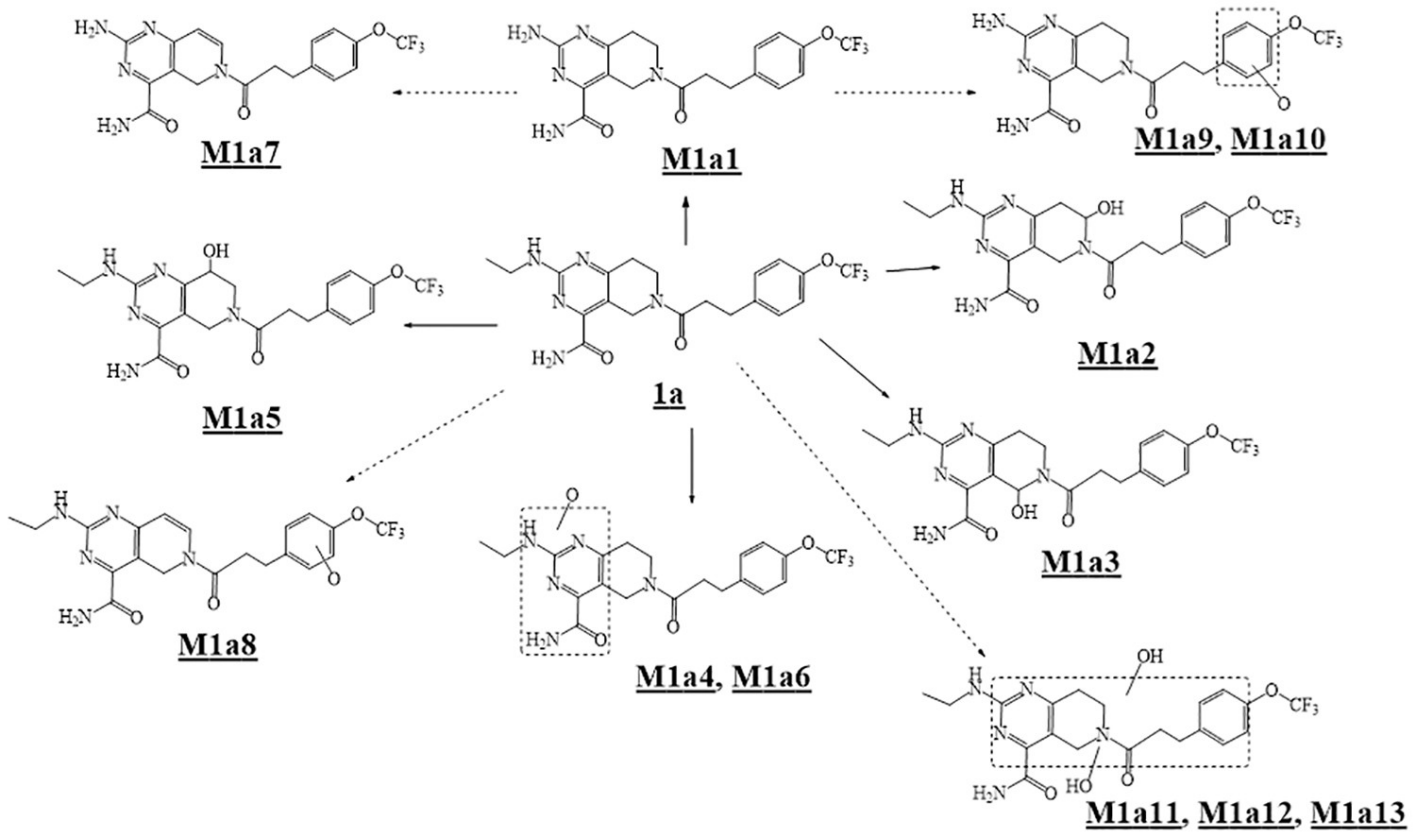
Scheme for metabolites of 1a observed with human liver microsomes.

Mass spectral data for the characterization of primary oxidation metabolites of **1a** and **2a** are presented in Supporting Information (S2 File). Fourteen metabolites were identified from **1a** in human liver microsomes (Fig 3, panel HLM). Metabolites M1a1-M1a6 result from primary oxidative reactions, and metabolites M1a7-M1a14 result from secondary oxidation of the primary oxidative products. A scheme for the oxidative metabolism of **1a** in human liver microsomes is shown in (Fig 4). Metabolite profiles with recombinant CYP isoforms showed that CYP3A4 formed the six primary metabolites, M1a1-M1a6 (Fig 3, panel CYP3A4). CYPs 2C9, 2C19, 2D6 and 1A2 formed the N-desethyl metabolite (M1a1) that was visible as a UV peak in the chromatograms (Fig 3 panels CYP2C9, CYP2C19, CYP2D6, and CYP1A2). Of six recombinant forms of CYP-enzymes examined, CYP 3A4 was the most active (Table 3). Although CYP 2C9 and 2C19 appear to be more active than CYP 3A4 in the N-deethylation of **1a** based on the UV signal (Fig 3, Panels CYP2C9 and CYP2C19), their contributions to overall clearance is minor when assessed by disappearance of substrate (Table 3) and when normalized to their levels in human liver microsomes relative to CYP3A4.

**Table 3:** Activities of recombinant human cytochrome P450 isoforms towards compounds 1a and 2a.

	1a		2a	
CYP isoform	k_obs_(min^-1^)	As % of CYP3A4	k_obs_(min^-1^)	As % of CYP3A4
CYP3A4	0.663	100	0.059	100
CYP1A2	0.009	1	<0.001	0
CYP2C9	0.050	7	<0.001	0
CYP2C19	0.071	11	0.014	24
CYP2D6	0.015	2	<0.001	0
CYP2C8	0.001	0	<0.001	0

**Fig 5:**
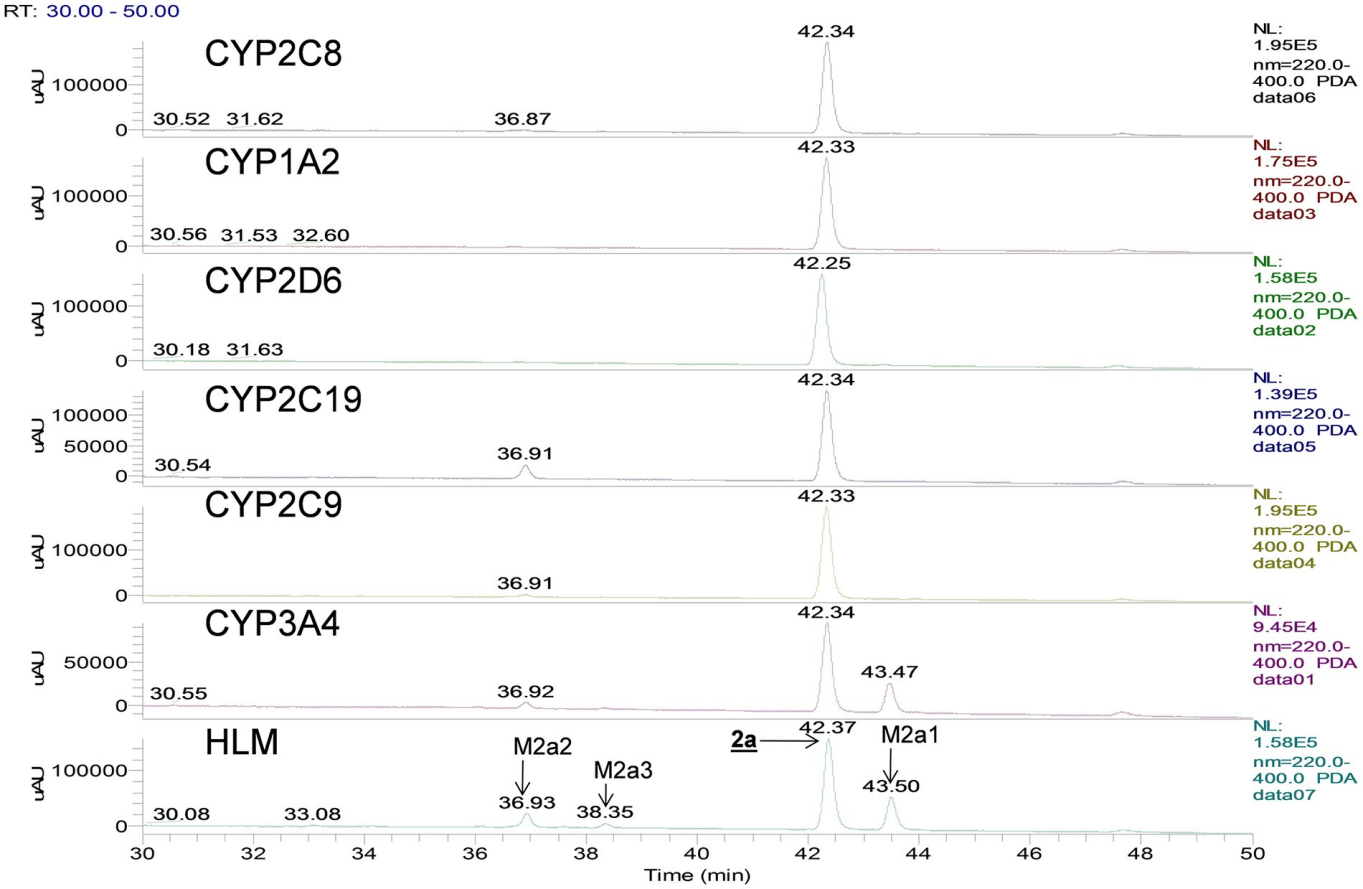
UV (300–400 nm) chromatograms for the metabolism of 2a in human liver microsomes and recombinant cytochromes 3A4, 2C9, 2C19, 2D6, and 1A2.

**Fig 6:**
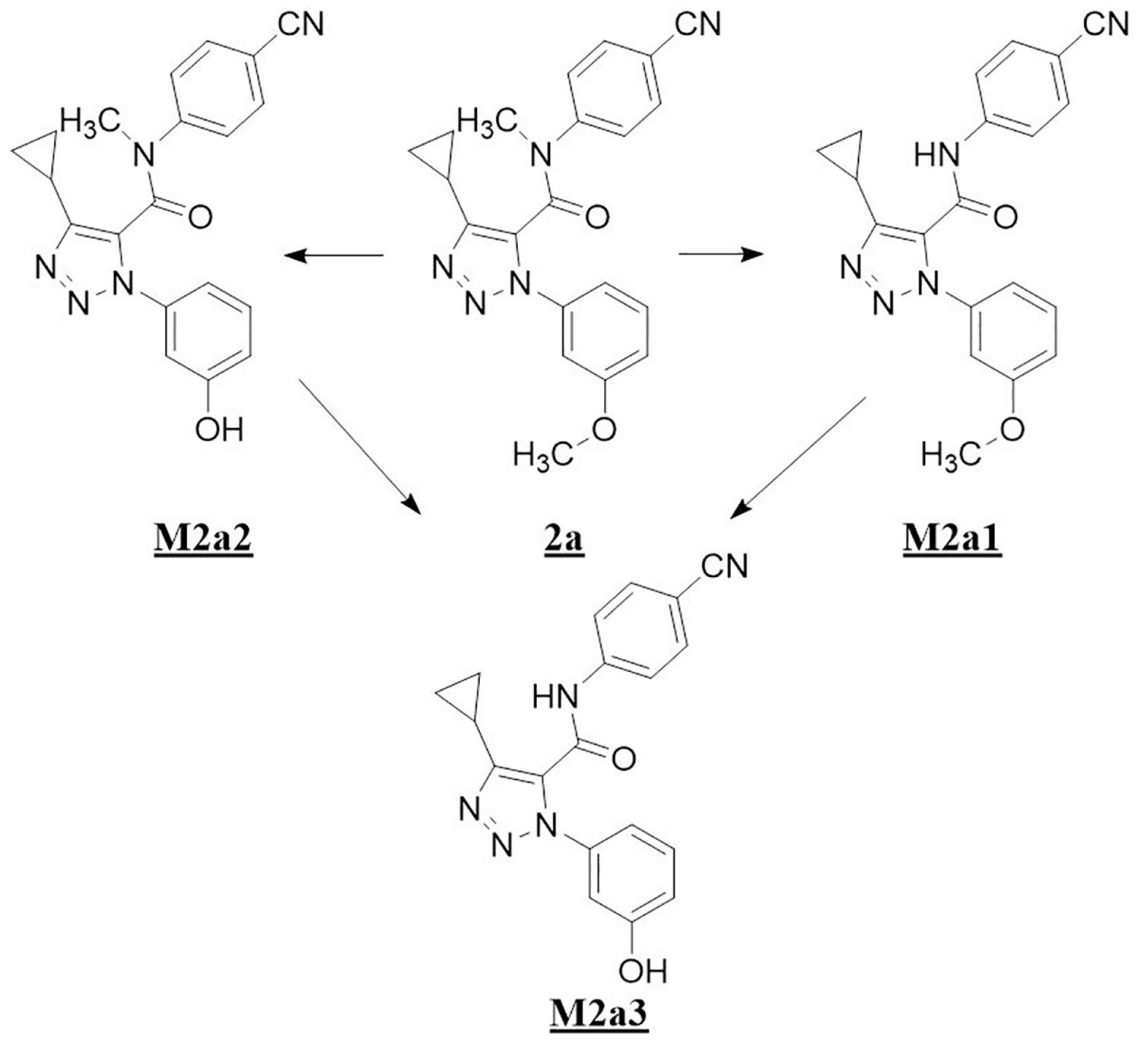
Scheme for metabolites of 2a observed with human liver microsomes.

Three metabolites were identified from **2a** in human liver microsomes. The primary oxidative demethylation products, N-desmethyl-**2a** (**M2a1**), the O-desmethyl-**2a** (**M2a2**), and the secondary metabolic product (**M2a3**) derived from either primary metabolite by further oxidative demethylation (Fig 5, panel HLM). Metabolite profiling with r-CYPs showed that CYP3A4 formed both primary metabolites with higher activity towards forming **M2a1** than **M2a2** (Fig 5, panel CYP3A4), whereas CYP2C19 was more active in forming **M2a2** than **M2a1** (Fig 5, panel CYP2C19). CYPs 2C9 and 2C8 produced only trace amounts of **M2a2**. Fig 6 shows a scheme for the metabolism of **2a**. As shown in Table 3, CYP3A4 was the most active of the six recombinant CYP-isoforms examined. CYP2C19 showed 24% of the activity of CYP3A4 and CYPs 2C9, 2D6, and 2C8, were essentially inactive. When normalized to their respective levels in liver microsomes, CYP3A4 is the primary isoform responsible for microsomal clearance of **2a**.

Accordingly, any strategy to address metabolic clearance by deuterium substitution in either chemotype requires a mechanistic consideration of the CYP3A4 reaction. As CYP2C19 showed 24% of the activity relative to CYP3A4 for **2a**, with the same metabolic profile but opposite preferences for the two metabolites, this isoform was also examined with **2a** and its deuterated forms to compare mechanistic similarities and differences from CYP3A4.

### Intrinsic clearance kinetic deuterium isotope effects and metabolite profiles with 1b-1f and 2b-2d

**Fig 7:**
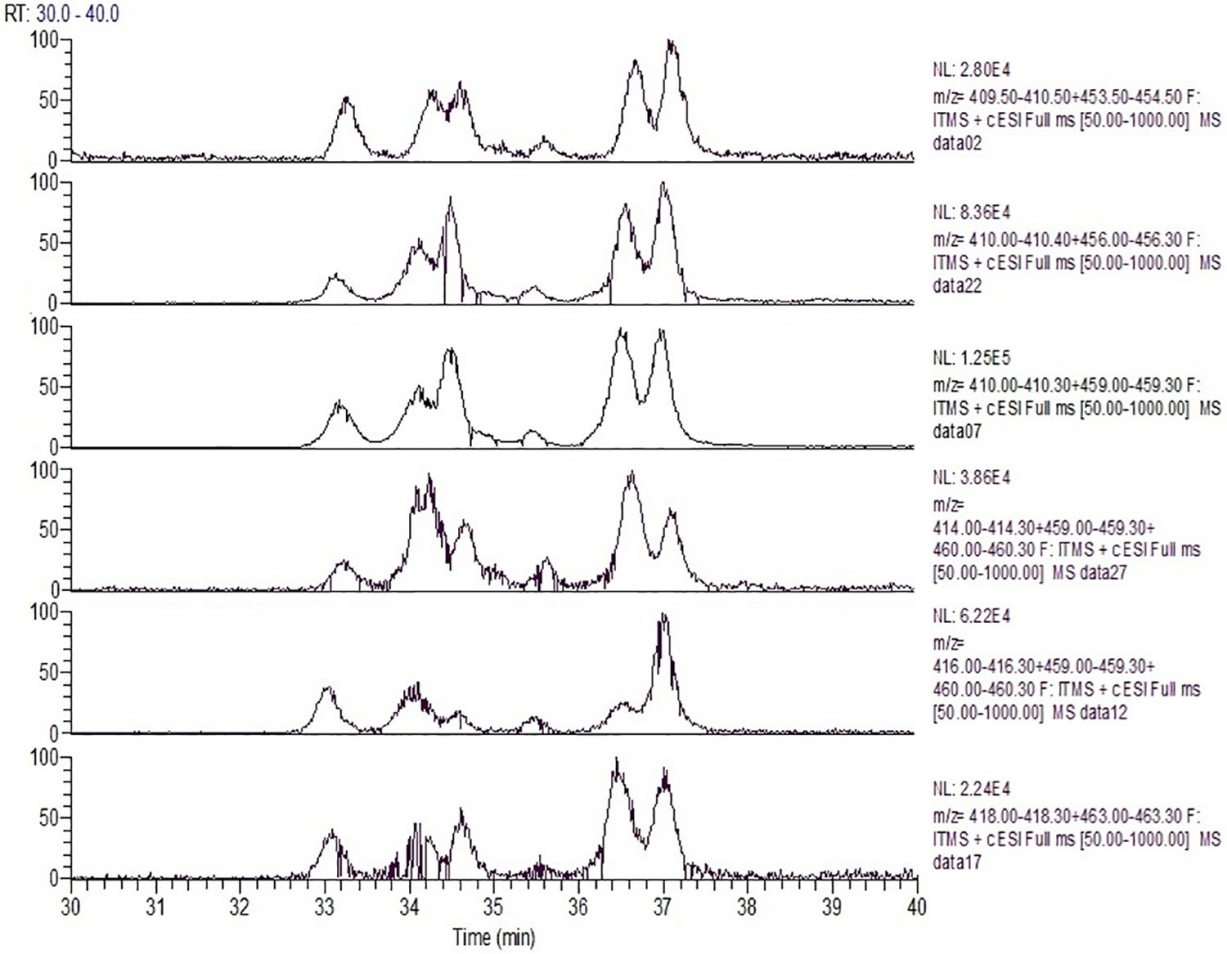
Extracted ion chromatograms for primary oxidative metabolites of 1a and its various deuterated forms with r-CYP3A4.

Results with chemotypes **1a**, **2a** and their deuterated forms (**1b**–**1f** and **2b**–**2d**) show how the chemo-type influences the course of reactions with CYP isoforms and their rate limiting steps. Table 4 summarizes the intrinsic clearance kinetic deuterium isotope effects observed for deuterated forms of **1a** (**1b**–**1f**) with human liver microsomes and recombinant CYP3A4. The lack of a deuterium isotope effect on the intrinsic clearance for any of the deuterated forms of **1a** with either enzyme system, indicates that there is no overall decrease in metabolism of this chemotype as a function of deuteration. Fig 7 shows the extracted ion chromatographic profiles for the identified metabolites from **1a-1f** and Table 5 shows their percentages relative to total metabolites. For each deuterated form, the relative abundance of each metabolite is an indication of the extent to which deuterium substitution has influenced metabolic switching. The pattern that emerges is complex. When deuterium is present at the methylene carbon of the N-ethyl group (**1b**, **1c**, **1d**, and, **1f**), N-deethylation is decreased between 1.4 and 2.5 fold. This decrease depends on the pattern of deuterium substitutions at other sites. With **1b** and **1d** where deuterium is present at the methylene of the ethyl group and additionally in **1d** at the methylenes adjacent to the piperidine ring nitrogen, the decrease in N-deethylation is comparable but metabolic switching occurs to hydroxylation sites at the pyrimidino-piperidine ring of **1d**. For **1b** switching is primarily to the benzylic methylene carbon adjacent to the piperidinyl nitrogen (**M1b3)**, whereas for **1d** it is to the deuterated methylene carbon adjacent to the piperidine nitrogen (**M1d2)**. With **1c** (where the N-ethyl group is per-deuterated) and **1f** (where the methylene of the N-ethyl is deuterated and the piperidine ring is per-deuterated), the decreases in N-deethylation are comparable but are less than with **1b** and **1d**. The metabolic switching is also distinct. For **1c** the metabolic switch occurs to the benzylic positions **M1c3** and **M1c5**, and for **1f** the major metabolic switch is to the benzylic methylene **M1f5** (deuterated). With **1e** where deuterium substitution is only at the methylenes adjacent to the piperidine nitrogen, the N-deethylation is only slightly increased, whereas the hydroxylation profile is substantially altered. Large decreases in **M1e3** (a deuterated site) and **M1e5** (a non-deuterated site) and increases in **M1e2** (a deuterated site) and **M1e6** (an isotope insensitive site) are observed. These results are interpreted in light of the two discrete binding modes revealed by molecular modeling (Fig 2 Panel A and Fig 2 Panel B). N-Deethylation occurs only from the binding mode shown in Fig 2 Panel B where the methylene of the N-ethyl is proximal to the activated heme iron-oxo complex. Deuteration of the methylene results in a decreased N-deethylation and increased oxidations on the pyrimidino-piperidine ring, consistent with metabolic switching between the two binding modes. By contrast, in the other binding cluster (Fig 2 Panel A) where the pyrimidino-piperidine ring is above the heme iron-oxo complex, the differences in metabolic product distribution observed for the various deuterated forms suggests subtle differences in orientation of the pyrimidino-piperidine ring as a consequence of deuterium in the different deuterated forms. In some instances, increases are observed in metabolites where C-D bonds are broken (**M1e2** from **1e**, and **M1d2**/**M1d5** from **1d**) which suggests higher constraints on substrates when bound in this binding cluster (Fig 2 Panel A). More importantly, the results suggest a high degree of ‘commitment to catalysis’ [27] in this binding cluster such that oxidation occurs at those sites that are most proximal to the active oxidant irrespective of them being deuterated, and the rate limiting step is past the catalytic event.

**Table 4:** Kinetic deuterium isotope effect on intrinsic clearance for deuterated forms of 1a with human liver microsomes and recombinant CYP3A4.

	^H^CL_int_ / ^D^CL_int_	
Compound	HLM	CYP3A4
(1b)	1.0	1.0
(1c)	1.0	1.1
(1d)	1.0	1.2
(1e)	1.0	1.0
(1f)	1.1	1.2

**Table 5:** Percentage of primary oxidative metabolites from deuterated forms of 1a with r-CYP3A4.

	Substrate					
	(1a)	(1b)	(1c)	(1d)	(1e)	(1f)
Metabolite	Metabolite as % of total metabolites					
M1x^1^1	18	9	13	7	20	13
M1x2	16	16	13	33	21	18
M1x3	13	23	22	12	5	11
M1x4	3	2	2	4	4	3
M1x5	25	26	30	31	14	35
M1x6	26	25	19	13	36	20

^1^ “x” refers to the form of substrate.

**Fig 8:**
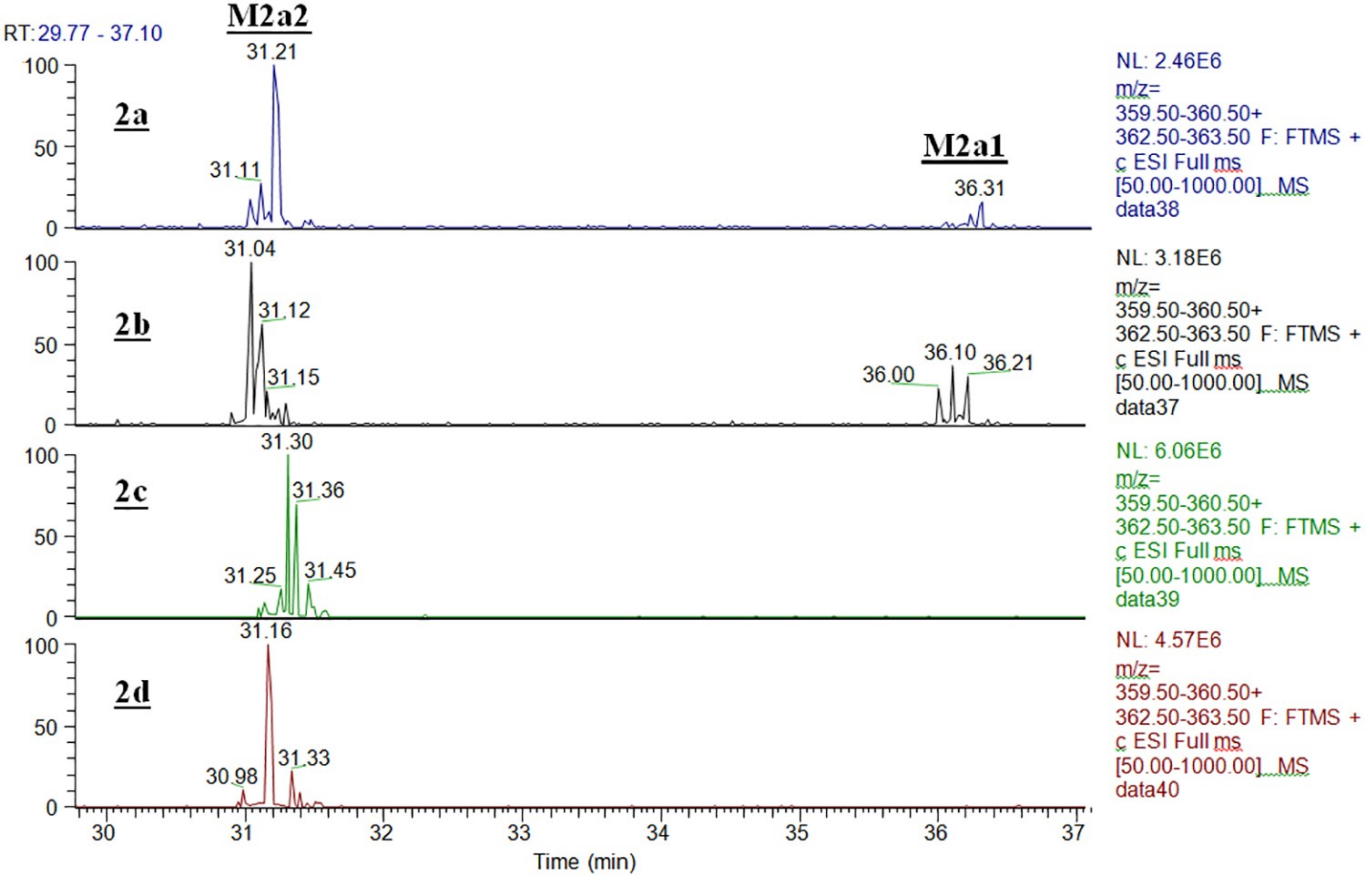
Extracted ion chromatograms for primary oxidative metabolites of 2a and its various deuterated forms (shown in Fig 1) with r-CYP2C19.

Since CYP2C19 showed 24% of the activity of CYP3A4 with chemotype **2a** (Table 3), and it has an active site substantially smaller than that of CYP3A4, isotope effects and product profiles were examined for comparison with both isoforms. Table 6 summarizes the intrinsic clearance deuterium isotope effect observed for **2b**–**2d**, with human liver microsomes, r-CYP3A4, and r-CYP2C19. With **2d**, where both methyl groups are deuterated, the isotope effect with all enzymatic systems is the largest. This indicates that the rate limiting step for this chemotype is C-H bond cleavage of either or both methyl groups. Changes in the magnitude of the isotope effect with r-CYPs 2C19 and 3A4 for substrates **2b** and **2c** where either methyl group is deuterated, indicates the complexity of isoform-dependent CYP mechanisms. For r-CYP 2C19 where O-demethylation is the major metabolic route (Fig 8, Panel 2a), the isotope effect on intrinsic clearance is 4.0 with **2b** where only the O-methyl group is deuterated and 4.5 with **2d** where both methyl groups are deuterated (Table 6), whereas the isotope effect is lost when only the N-methyl group is deuterated (**2c**, Table 6). The isotope effects are mirrored in the metabolic profiles shown in Fig 8. When only the O-methyl group is deuterated (**2b**) the N-des-methyl metabolite (**M2b1**) level is slightly higher than that from the non-deuterated form (**2a**, Fig 8 Panels 2a and 2b), suggesting metabolic switching. Whereas the N-des-methyl metabolites (**M2c1** and **M2d1**) are essentially lost when the N-methyl groups in **2c** and **2d** are deuterated (Fig 8, Panels 2c and 2d). These metabolic profiles and isotope effects are consistent with the low contribution of the **M2a1** metabolic pathway to the overall metabolism by CYP2C19.

**Table 6:** Kinetic deuterium isotope effect on intrinsic clearance for deuterated forms of 2a with human liver microsomes, r-CYP3A4 and r-CYP2C19.

	^H^K / ^D^K		
Compound	HLM	CYP3A4	CYP2C19
(**2b**)	1.3	1.2	4.0
(**2c**)	1.5	2.9	1.0
(**2d**)	3	8.3	4.5

**Fig 9:**
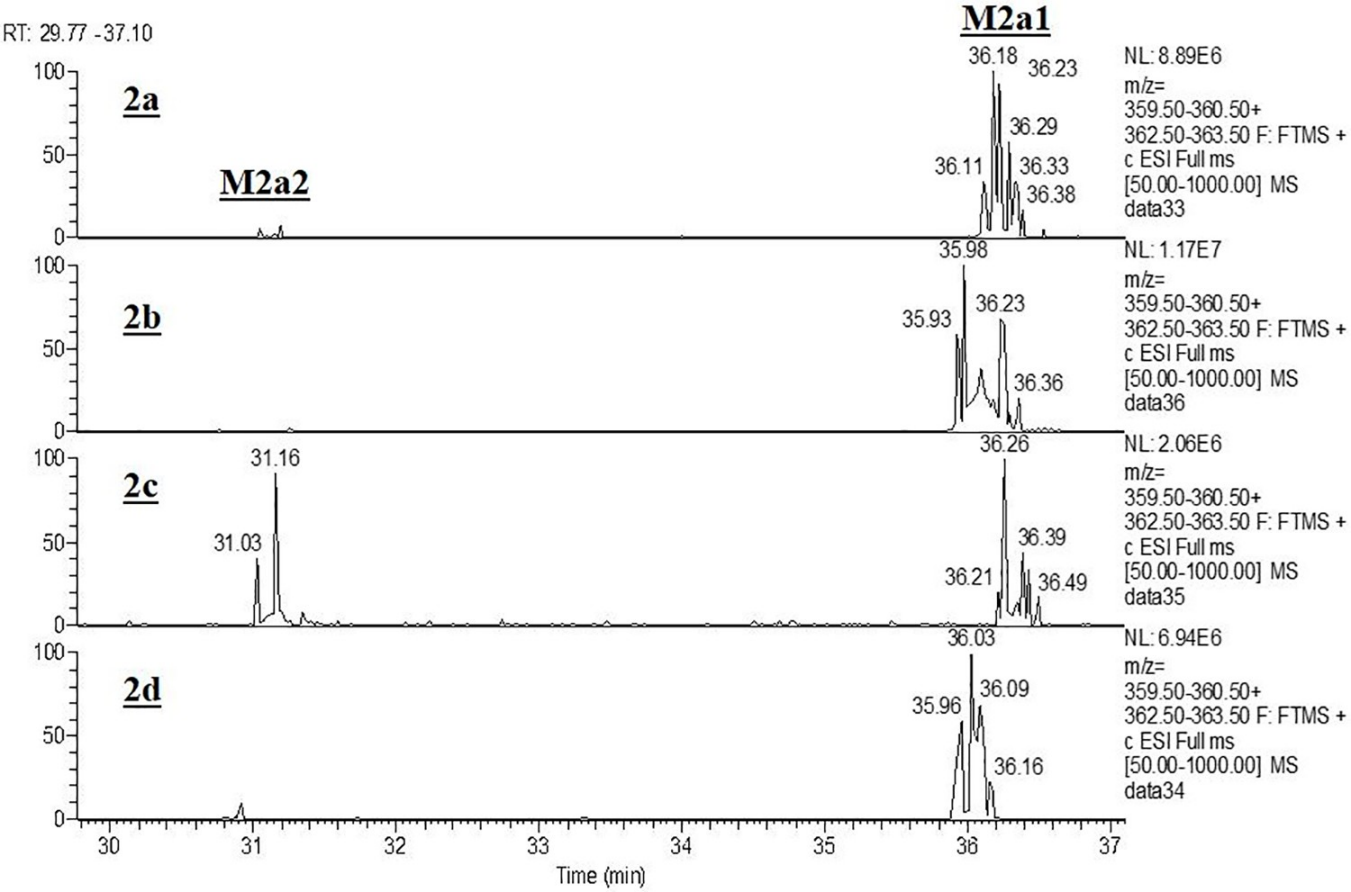
Extracted ion chromatograms for primary oxidative metabolites of 2a and its various deuterated forms (shown in Fig 1) with r-CYP3A4.

For CYP3A4 where N-demethylation (**M2a1**) is the preferred metabolic route (Fig 5 Panel 3A4, and Fig 9 Panel 2a), deuteration of only the O-methyl (**2b**), or N-methyl (**2c**) groups result in a decrease in the intrinsic clearance isotope effect when compared to deuteration at both methyl groups (Table 6, **2d**). This suggests that for CYP3A4, metabolic switching substantially influences the magnitude of the intrinsic clearance isotope effect. As shown in Fig 9, when either the O-methyl group alone (Fig 9 Panel 2b) or both methyl groups are deuterated (Fig 9, Panel 2d), the minor pathway resulting in the O-desmethyl product (**M2a2**) is essentially lost, whereas when only the N-methyl group is deuterated, the N-desmethyl product (**M2a1**) is decreased and the minor O-desmethyl product (**M2a2**) is increased (Fig 9, Panel 2c). These results are consistent with the observed differences in the intrinsic clearance isotope effects in Table 6 and show that for this chemotype and CYP3A4, oxidation at the methyl groups is the rate limiting step, but metabolic switching can limit the magnitude of the intrinsic clearance isotope effect. These results also demonstrate that when multiple CYPs are involved in the metabolism, their individual contributions to clearance and mechanistic nuances need to be understood in order to gain pharmacokinetically from deuteration.

## Discussion

While substitution of deuterium for hydrogen without impactful changes in physico-chemical properties of drug molecules is an attractive proposition for improving the pharmacokinetic or toxicological properties of drugs, differences in reaction mechanisms of the enzymes involved in their metabolic clearance can impact the outcome. The component of a deuteration strategy examined in this study is the in vitro approach early in the drug discovery process that involves mechanistic aspects of the CYP family of enzyme(s) responsible for metabolic clearance. These studies demonstrate how before the synthesis of deuterated forms of NCEs are undertaken, knowledge of the CYP isoforms and their relative contribution to the metabolic clearance can provide insights through molecular docking analysis as to the sites of metabolism and the potential for metabolic switching. When coupled to metabolic profiling these efforts provide a better understanding of which deuterated forms need to be synthesized. For example, for chemotype **1a** and **2a**, CYP3A4 was determined to be the isoform responsible for a major component of the clearance. Accordingly, focusing on CYP 3A4 alone would suffice for analysis of the requirements for deuteration. Molecular modelling of **1a** with CYP3A4 showed two distinct binding clusters where either the pyrimidino-piperidine ring or the N-ethyl is proximal to the heme iron. Protein constraints on these modes indicated that “tumbling” within the active site is unlikely. Consequently, for metabolic switching to occur, bound and unbound substrate would require to be in rapid equilibrium (low commitment to catalysis). Additionally, modeling also showed that hydroxylation could occur at multiple sites on the pyrimidino-piperidine ring. The metabolic profiles with microsomes and rCYP 3A4 were consistent with the two major binding clusters determined by molecular docking and accounted for the diversity of metabolites observed. More importantly, the results showed that while metabolic switching between N-deethylation and pyrimidino-piperidine ring hydroxylation orientations does occur, binding clusters with the pyrimidino-piperidine exposed to the heme-oxo species appear to be more ‘committed to catalysis’ [28] as evidenced by C-D bonds being hydroxylated in preference to available C-H bonds. Less obvious and unlikely to be assessed only from kinetic analysis of the protio form of the substrate is which step(s) in the reaction mechanism is rate-limiting. Thus, for chemotype **1a**, studies conducted in this report with deuterated forms **1b** and **1d** or **1e** would have provided enough information for assessment that deuteration for this chemo-type would result in no pharmacokinetic advantage due to a mechanism that possibly involves a higher commitment to catalysis, and a rate limiting step that is past the catalytic event that masks the intrinsic isotope effect (^*H*^*k*/^*D*^*k* for the catalytic step) on the intrinsic clearance. Accordingly, extensive deuteration efforts could be avoided. Molecular modelling of **2a** with CYP 3A4 showed a single binding mode could result in oxidation of either methyl group, predicting metabolic switching, and therefore requiring deuteration of both methyl groups to potentially gain from a deuteration strategy. Metabolic profiling and the intrinsic clearance isotope effects with the various deuterated forms confirmed these predictions. However, without the deuterated forms of **2a** it would not be possible to assess if the intrinsic isotope effect would be expressed on the intrinsic clearance.

The results reported here are not unique to the chemotypes examined in this study, a similar observation was reported for the CYP-catalyzed oxidation of benzylic alcohols, where the rate limiting step was determined by the substrate and varied with the CYP isoform examined [28]. The impact of this, and other studies, where deuteration has failed to demonstrate a gain in pharmacokinetic advantage, demonstrates that in addition to a clear understanding of the systemic clearance mechanism, a clear understanding of the enzyme reaction mechanism for the substrates under consideration, particularly when CYP enzymes are involved, is critical to the application of a deuteration strategy to enhance pharmacokinetic properties.

## Supporting information

S1 File Figure A Synthesis of intermediate S8 for 1a. Figure B. Synthesis of 1a. Figure C. Synthesis of 1e. Figure D. Synthesis of intermediate S17 for 1b and 1c. Figure E. Synthesis of 1d. Figure F. Synthesis of 1f. Figure G. 2a. Figure H. 2b. Figure I. 2c. Figure J. 2d. Figure K. ^1^H NMR of intermediate S17. Figure L. ^1^H NMR of intermediate S20. Figure M. ^1^H NMR of intermediate S21. Figure N. ^1^H NMR of intermediate S22. Figure O. ^1^H NMR of intermediate S26. Figure P. ^1^H NMR of intermediate S27. Figure Q. ^1^H NMR of compound 1b. Figure R. ^1^H NMR of compound 1c. Figure S. ^1^H NMR of compound 1d. Figure T. ^1^H NMR of compound 1e. Figure U. ^13^C NMR of compound 1e. Figure V. ^1^H NMR of compound 1f. Figure W. ^13^C NMR of compound 1f. Figure X. ^1^H NMR of compound 2b. Figure Y. ^13^C NMR of compound 2b. Figure Z. ^1^H NMR of compound 2c. Figure AA. ^13^C NMR of compound 2c. Figure AB. ^1^H NMR of compound 2d. Figure AC. ^13^C NMR of compound 2d.
(PDF)

S2 File **Figure A. M1a1**. UV retention time 33.31 minutes. **Figure B. M1a2**. UV retention time 34.8 minutes. **Figure C. M1a3**. UV retention time 34.40 minutes. **Figure D. M1a4**. UV retention time 35.9 minutes. **Figure E. M1a5**. UV retention time 36.8 minutes. **Figure F. M1a6**. UV retention time 37.26 minutes. **Figure G. M2a1**. UV retention time 43.5 minutes. **Figure H. M2a2**. **Figure I. M2a3**. UV retention time 30.83 minutes.
(PDF)
