# Supplementary material for: Deuterium isotope effects in drug pharmacokinetics II: Substrate-dependence of the reaction mechanism influences outcome for cytochrome P450 cleared drugs
Source: PLoS One. 2018 Nov 14;13(11):e0206279. doi: 10.1371/journal.pone.0206279 (PMC6235261; doi:10.1371/journal.pone.0206279)
Supplement: S1 File — Figure A Synthesis of intermediate S8 for 1a. Figure B. Synthesis of 1a. Figure C. Synthesis of 1e. Figure D. Synthesis of intermediate S17 for 1b and 1c. Figure E. Synthesis of 1d. Figure F. Synthesis of 1f. Figure G. 2a. Figure H. 2b. Figure I. 2c. Figure J. 2d. Figure K. 1H NMR of intermediate S17. Figure L. 1H NMR of intermediate S20. Figure M. 1H NMR of intermediate S21. Figure N. 1H NMR of intermediate S22. Figure O. 1H NMR of intermediate S26. Figure P. 1H NMR of intermediate S27. Figure Q. 1H NMR of compound 1b. Figure R. 1H NMR of compound 1c. Figure S. 1H NMR of compound 1d. Figure T. 1H NMR of compound 1e. Figure U. 13C NMR of compound 1e. Figure V. 1H NMR of compound 1f. Figure W. 13C NMR of compound 1f. Figure X. 1H NMR of compound 2b. Figure Y. 13C NMR of compound 2b. Figure Z. 1H NMR of compound 2c. Figure AA. 13C NMR of compound 2c. Figure AB. 1H NMR of compound 2d. Figure AC. 13C NMR of compound 2d. (PDF) [file pone.0206279.s001.pdf]

S1 Supporting Information

Deuterium isotope effects in drug pharmacokinetics II: Substrate-dependence of the reaction mechanism influences outcome for cytochrome P450 cleared drugs

Hao Sun,* David W. Piotrowski*, Suvi T. M. Orr, Joseph S. Warmus, Angela C. Wolford, Stephen B. Coffey, Kentaro Futatsugi, Yinsheng Zhang and Alfin D. N. Vaz*

Medicine Design, Pfizer Global Research and Development, Groton, Connecticut, United States of America

General Experimental Details

All chemicals, reagents and solvents were purchased from commercial sources when available and used without purification. Proton (^{1}H NMR) and carbon (^{13}C NMR) nuclear magnetic spectroscopy were recorded with Varian or Bruker spectrometers. Chemical shifts are expressed in parts per million (δ) relative to deuterated solvent as an internal standard. The peak shapes are denoted as follows: s, singlet; d, doublet; t, triplet; q, quartet; m, multiplet; br s, broad singlet. Due to differences in solvents used in sample preparation and, in some cases amount of water present, not all exchangeable protons were observable. Mass spectrometry (MS) was performed via atmospheric chemical ionization (APCI) or electron scatter (ES) ionization sources. High resolution mass spectrometry was performed at NovaBioAssays (Woburn, MA) on Waters UPLC with TUV and Thermo Q Exactive MS instruments. Liquid mass spectrometry (LCMS) was performed on Agilent 1100 Series. Silica gel chromatography was performed using a medium pressure Biotage or ISCO Combiflash system, and Whatman pre-coated silica gel plates (250 μm) were used for analytical TLC. The synthesis of compounds **1a**,[1] **2a**,[2] and associated

intermediates, **S2-S8** and **S11-S15**,[1] have been reported previously. Compounds **S1** (Boc-piperidin-4-one) and **S9** (4-trifluoromethoxyhydrocinnamic acid) were purchased from commercial sources as used as received.

Experimental procedures for compounds (1a-1f)

Fig A.

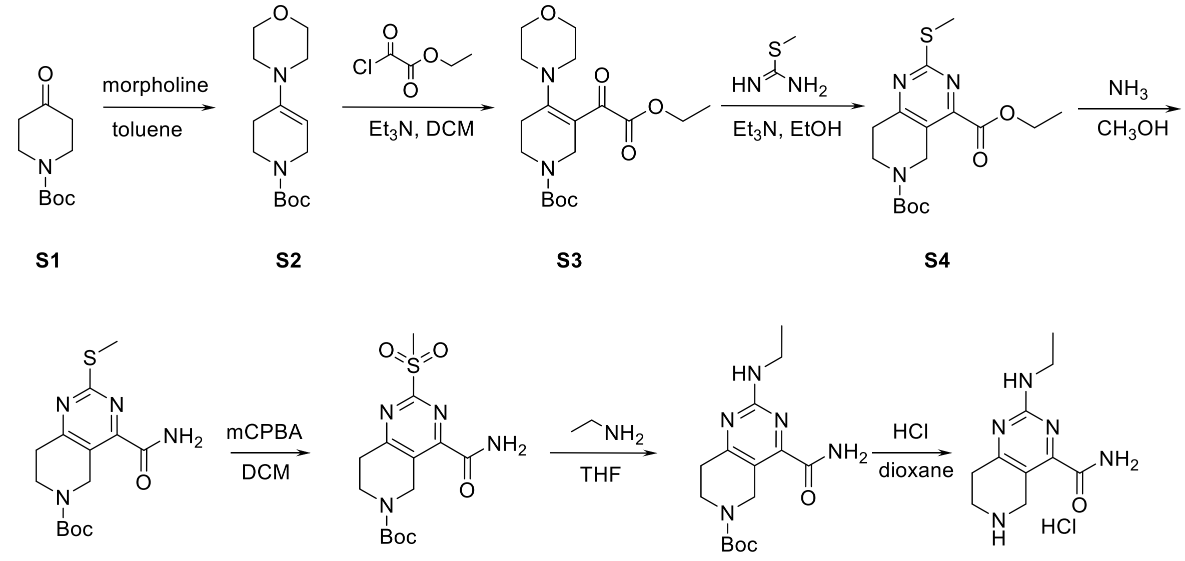

To a solution of Boc-piperidin-4-one **S1** (500 g, 2.51 mol) in toluene (2 L) was added morpholine (250 g, 2.76 mol) and the flask was equipped with a Dean-Stark trap and the mixture was stirred under reflux for 18 h. The mixture was cooled to room temperature, and the solvent was removed under reduced pressure to give enamine **S2** (~720 g) as a yellow liquid. This material was used without further purification.

To a solution of enamine **S2** (~720 g) and triethylamine (327 g, 3.23 mol) in dichloromethane (2 L) was added ethyl oxalyl chloride (405 g, 2.96 mol) dropwise at 0 °C, the mixture was allowed to warm to room temperature while stirring for 18 h. The mixture was filtered and the filtrate was

concentrated under reduced pressure to give compound **S3** (~250 g) as a yellow solid. This material was used without further purification.

To a solution of compound **S3** (~1100 g, ~3 mol) in ethanol (4 L) was added S-methylthiourea (562 g, 3 mol) and triethylamine (1200 g, 11.9 mol) and the mixture was stirred under reflux for 18 h. The mixture was cooled to room temperature and filtered to give a yellow solid that was washed with dichloromethane (2 L). The filtrate was concentrated under reduced pressure to give compound **S4** (~250 g) as a yellow solid. This material was used without further purification.

To a solution of compound **S4** (~120 g, 340 mmol) in methanol (3 L) was added a solution of 7 N ammonia in methanol (500 mL, 3500 mmol), and the mixture was stirred in a sealed bottle at 40 °C for 18 h. The mixture was cooled to room temperature and the solvent was removed under reduced pressure to give compound **S5** (~110 g) as a white solid. This material was used without further purification.

To a solution of compound **S5** (~108 g, ~333 mmol) in dichloromethane (2 L) was added m-chloroperbenzoic acid (145 g, 767 mmol) in portions at 0 °C, and the mixture was allowed to warm to room temperature while stirring for 2 h. The reaction mixture was poured into a separatory funnel and washed with sat. aq. $Na_2S_2O_3$ (2 x 300 mL), sat. aq. K_2CO_3 (2 x 300 mL), dried (Na_2SO_4), filtered and concentrated under reduced pressure to give compound **S6** (~118 g) as a white solid. This material was used without further purification.

To a solution of compound **S6** (~40 g, ~112 mmol) in THF (1.5 L) was added ethylamine (68 g, 1500 mol), and the mixture was stirred at 80 °C for 18 h. The solvent was removed under reduced pressure and the residue was crystallized from THF and ethyl acetate to give compound **S7** (22 g, 61%) as a white solid. ^{1}H NMR ($CDCl_3$, 400 MHz) δ 7.75 (br s, 1 H), 5.56 (br s, 1 H),

5.06 (br s, 1 H), 4.95-4.91 (m, 2 H), 3.72-3.67 (m, 2 H), 3.47-3.40 (m, 2 H), 2.84-2.82 (m, 2 H), 1.48 (s, 9 H), 1.27-1.19 (m, 3 H).

To a solution of compound **S7** (140 g, 436 mmol) in dichloromethane (400 mL) was added 4.0 M HCl in dioxane (1.5 L) dropwise at 0 °C, and the mixture was stirred at room temperature for 6 h. The mixture was filtered to give a solid that was further dried in a vacuum oven to give compound **S8** (122 g, 96%) as a white solid. ^{1}H NMR (D_2O, 400 MHz) δ 4.47 (s, 2 H), 3.47 (t, *J* = 6.8 Hz, 2 H), 3.35 (q, *J* = 7.2 Hz, 2 H), 3.05 (t, *J* = 6.8 Hz, 2 H), 1.09 (t, *J* = 7.2 Hz, 3 H); m/z = 222.1 $(M+H)^+$.

Fig B.

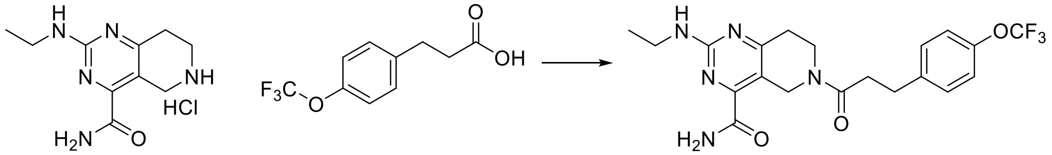

S8 **S9** **1a**

To a solution of the amine **S8**-HCl salt (10.0 g, 34 mmol) in dichloromethane (250 mL) were added 4-trifluoromethoxyhydrocinnamic acid (**S9**) (8.9 g, 38 mmol), triethylamine (12 mL, 86 mmol), DMAP (5.2 g, 41 mmol), and EDCI (9.7 g, 48 mmol). The resulting mixture was stirred at room temperature for 18 h. The reaction mixture was diluted with ethyl acetate (300 mL) and 1 M aqueous HCl solution (300 mL) was added. The layers were separated and the organic layer was washed with 1N NaOH (300 mL), brine (300 mL), dried (Na_2SO_4), filtered and concentrated under reduced pressure to give a clear oil. The crude material was purified by ISCO Combiflash system eluting with heptanes/acetone gradient to give 13.2 g of compound **1a** as clear oil that solidified upon standing. This material was recrystallized from acetonitrile to give 8.25 g (56%) of **1a**[1] as a white solid. mp 132-133 °C. ^{1}H NMR ($CDCl_3$, 400 MHz) δ 9.70 (br s, 1H), 7.18 (d, *J*

= 8 Hz, 2H), 6.98 (d, *J* = 8 Hz, 2H), 4.68 (br s, 1H), 4.11 (q, *J* = 7.0 Hz, 2H), 3.46-3.42 (m, 1H), 3.10-2.96 (m, 2H), 2.76-2.69 (m, 1H), 2.03-1.97 (m, 3H), 1.65-1.62 (m, 2H), 1.24 (t, *J* = 7.0 Hz, 3H); ^{13}C NMR ($CDCl_3$, 100.5 MHz) δ 170.9, 168.1, 167.2, 159.7, 150.7, 147.5, 139.7, 129.8, 120.8, 120.4 (q, $^1J_{CF}$ = 257 Hz), 115.6, 44.7, 38.5, 36.3, 35.0, 32.2, 30.9, 14.8; ^{19}F NMR ($CDCl_3$, 376.5 MHz) δ -57.9; HPLC (purity) 99.3%; HRMS Calcd. for $C_{20}H_{22}F_3N_5O_3$ $(M+H)^+$ 438.1748; Found 438.1734; Anal. Calcd. for $C_{20}H_{22}F_3N_5O_3$: C; 54.92, H; 5.07, N; 16.01. Found, C; 54.91, H; 5.01, N; 16.00.

Fig C.

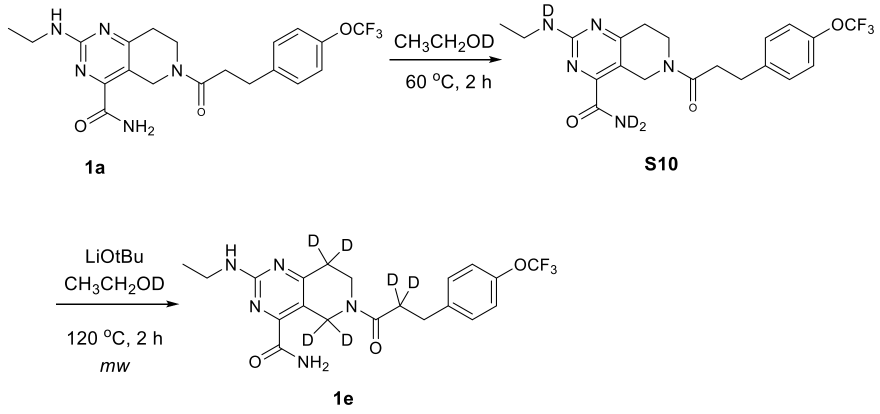

To a microwave vial was added compound **1a** (200 mg, 0.46 mmol) in deuterated ethanol (CH_3CH_2OD, 4 mL). The solution was heated at 60 °C for 2 h under N_2 atmosphere. The solvent was removed under reduced pressure to provide crude **S10**. The residue was dissolved in deuterated ethanol (CH_3CH_2OD, 8 mL) and LiOtBu (25 mg, 0.31 mmol) was added. The resulting mixture was heated in a microwave reactor (200 W) at 120 °C for 2 h. The solvent was removed under reduced pressure to give a crude product, which was purified via silica gel column chromatography by eluting with EtOAc/hexane gradient to give **1e** as a white solid (150 mg, 74%). ^{1}HNMR (400 MHz, $CDCl_3$) δ 7.11 (d, *J* = 8.6Hz, 2 H), 6.94 (d, *J* = 8.0 Hz, 2 H), 3.90 (s, 2 H), 3.37 (q, *J* = 7.2 Hz, 2 H), 2.95 (s, 2 H), 1.27 (t, *J* = 7.2 Hz, 3 H); ^{13}CNMR (100

MHz, $CDCl_3$): 171.0, 166.4, 159.8, 147.5, 139.6, 133.0, 129.9, 121.1, 120.8, 119.1, 115.3, 41.9, 36.5, 34.6, 30.9, 29.8, 29.7, 14.5. m/z = 444.4 $(M+H)^+$. HRMS Calcd. for $C_{20}H_{17}D_6F_3N_5O_3$ $(M+H)^+$ 444.2124; Found 444.2117.

Fig D.

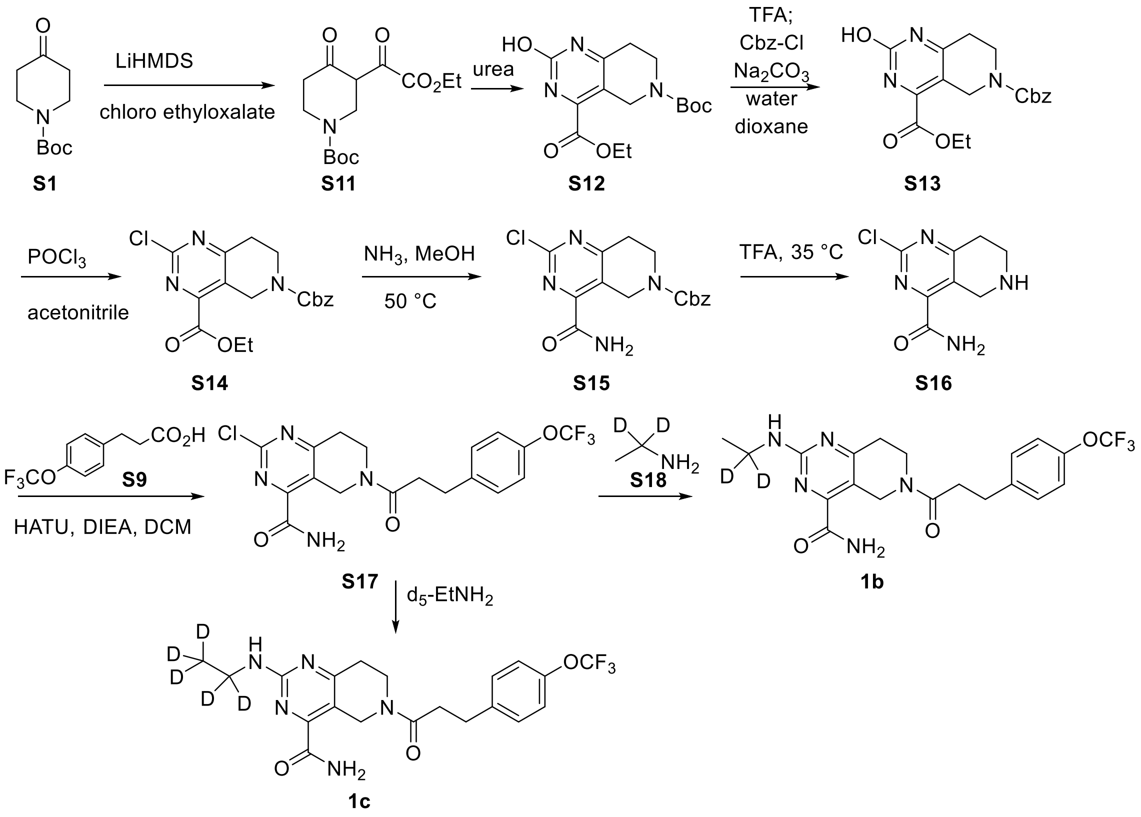

To a solution of N-Boc-piperid-4-one (**S1**) (1.5 kg, 7.5 mol) in THF (1 L) was added LiHMDS (7.5 L, 7.5 mol) dropwise at -78 °C under N_2 atmosphere, and the mixture was stirred for 1 h. Ethyl chlorooxalate (1.1 kg, 7.5 mol) was added at -78 °C, and stirring was continued for 5 h while allowing the cooling bath to warm to room temperature. Water (1 L) was added and the mixture was extracted with MTBE (2 x 1 L). The combined organic layers were washed (brine), dried (Na_2SO_4), filtered and concentrated under reduced pressure to give compound **S11** (1.5 kg, 67%) as a yellow oil.

To a solution of compound **S11** (500 g, 1.67 mol) in AcOH (1 L) was added urea (150 g, 2.51 mol) and the mixture was stirred at 50 °C for 18 h. The solvent was removed under reduced pressure and the residue was dissolved in dichloromethane (500 mL). The organic layer was washed with water (500 mL), dried (Na_2SO_4), filtered and concentrated to give a residue that was purified via silica gel chromatography eluting with dichloromethane/methanol (20:1) to give **S12** (150 g) as a black solid.

To a solution of compound **S12** (156 g, 0.48 mol) in dichloromethane (1.5 L) was added TFA (550 g, 4.8 mol) at room temperature, and the mixture was stirred for 18 h. The solvent was removed under reduced pressure to give 167 g of a residue that was used in the subsequent step without further purification. This material was dissolved in water (500 mL) and dioxane (500 mL). Benzyl chloroformate (127 g, 0.74 mol) and Na_2CO_3 (106 g, 1.0 mol) were added and the mixture was stirred at room temperature for 18 h. Water (300 mL) was added and the mixture was extracted with dichloromethane (2 x 500 mL). The combined organic layers were washed (brine), dried (Na_2SO_4), filtered and concentrated to give a residue that was purified via silica gel column chromatography eluting with dichloromethane/methanol (20:1) to give **S13** as a black solid.

To a solution of **S13** (45 g, 126 mmol) in acetonitrile (500 mL) was added $POCl_3$ (45 mL, 491 mmol) and the mixture was heated under reflux for 2 h. The solvent was removed under reduced pressure and the residue was purified via silica gel column chromatography, eluting with petroleum ether/ethyl acetate (3:1) to give **S14** (22.7 g, 50%) as a yellow solid. ^{1}HNMR (400 MHz, $CDCl_3$) δ 7.37-7.31 (m, 5 H), 5.18 (s, 2 H), 4.96 (s, 2 H), 4.46 (q, *J* = 7.2 Hz, 2 H), 3.84 (t, *J* = 6.0 Hz, 2 H), 3.06 (br s, 2 H), 1.41 (t, *J* = 7.2 Hz, 3 H); m/z = 376.0 $(M+H)^+$.

A solution of compound **S14** (15 g, 39 mmol) in 7 N ammonia in methanol (50 mL, 6 mol) was heated at 50 °C in a sealed bottle for 2.5 h. The mixture was cooled to room temperature, and filtered by washing with methanol (20 mL). The solid was dried in a vacuum oven to give 13 g (99%) of compound **S15** as a green solid. This material was used without further purification.

A mixture of compound **S15** (2 g, 5.7 mmol) and trifluoroacetic acid (20 mL) was stirred at 35 °C for 16 h. The solvent was removed under reduced pressure to give compound **S16** (2.0 g) as a brown oil, which was used without further purification.

To a solution of compound **S16** (920 mg, 4.3 mmol) in anhydrous dichloromethane (50 mL) was added 4-trifluoromethoxyhydrocinnamic acid (**S9**, 1010 mg, 4.3 mmol), diisopropylethylamine (1650 mg, 13 mmol) and HATU (1640 mg, 4.3 mmol), and the mixture was stirred at room temperature for 15 h. The solvent was removed under reduced pressure and the residue was purified via silica gel column chromatography, eluting with petroleum ether/ethyl acetate (1:1) to give compound **S17** (1250 mg, 67%) as a white solid. ^{1}HNMR (400 MHz, CD_3OD) δ 7.24 (d, *J* = 8.0 Hz, 1 H), 7.14 (d, *J* = 8.0 Hz, 1 H), 7.05 (d, *J* = 8.0 Hz, 1 H), 6.89 (d, *J* = 8.0 Hz, 1 H), 5.04 (s, 1 H), 4.92 (s, 1 H), 3.78-3.71 (m, 2 H), 2.87-2.81 (m, 4 H), 2.74-2.69 (m, 2 H); m/z = 428.8 $(M+H)^+$.

Synthesis of Intermediate S18 used for 1b and 1d

Acetonitrile (450 mg, 10.7 mmol) was added to a solution of $LiAlD_4$ (1 g, 23 mmol) in anhydrous THF (15 mL), and the mixture was stirred at room temperature for 2 h. Water (8 mL) was added followed by bubbling through a saturated solution of HCl in methanol at 0 °C. The mixture was concentrated under reduced pressure to give compound **S18** (70 mg) as a yellow solid. This material was used without further purification.

Synthesis of 1b

To a solution of compound **S17** (50 mg, 0.12 mmol) in DMF (2 mL) was added compound **S18** (30 mg, 0.36 mmol), CsF (50 mg, 0.33 mmol), and diisopropylethylamine (80 mg, 0.63 mmol), and the mixture was stirred at 80 °C for 18 h. The mixture was filtered and the filtrate was concentrated under reduced pressure. The residue was purified via preparative HPLC to give compound **1b** (15 mg, 29%) as a white solid. ^{1}H NMR (CD_3OD, 400 MHz) δ 7.34 (d, *J* = 8.4 Hz, 1 H), 7.24 (d, J = 8.4 Hz, 1 H), 7.17 (d, *J* = 8.4 Hz, 1 H), 7.02 (d, *J* = 8.4 Hz, 1 H), 4.96 (br s, 2 H), 3.82-3.72 (m, 2 H), 3.01-2.87 (m, 2 H), 2.83-2.69 (m, 4 H), 1.20 (s, 3 H); m/z = 440.1 $(M+H)^+$. HRMS Calcd. for $C_{20}H_{21}D_2F_3N_5O_3$ $(M+H)^+$ 440.1873; Found 440.1872.

Synthesis of 1c

To a mixture of compound **S17** (50 mg, 23 mmol) in DMF (2 mL) was added K_2CO_3 (50 mg, 36 mmol) and d_5-ethylamine-1,1,2,2,2 (100 mg, 23 mmol), and the mixture was stirred at room temperature for 16 h. The mixture was filtered and the crude product was purified by preparative HPLC to give compound **1c** (8.7 mg, 15%) as a white solid. ^{1}HNMR (CD_3OD, 400 MHz) δ 7.34 (d, *J* = 8 Hz, 1 H), 7.24 (d, *J* = 8 Hz, 1 H), 7.16 (d, *J* = 8 Hz, 1 H), 7.01 (d, *J* = 8 Hz, 1 H), 4.98-4.95 (m, 1 H), 3.80-3.75 (m, 2 H), 2.99-2.73 (m, 7 H). m/z = 443.1 $(M+H)^+$.

Fig E.

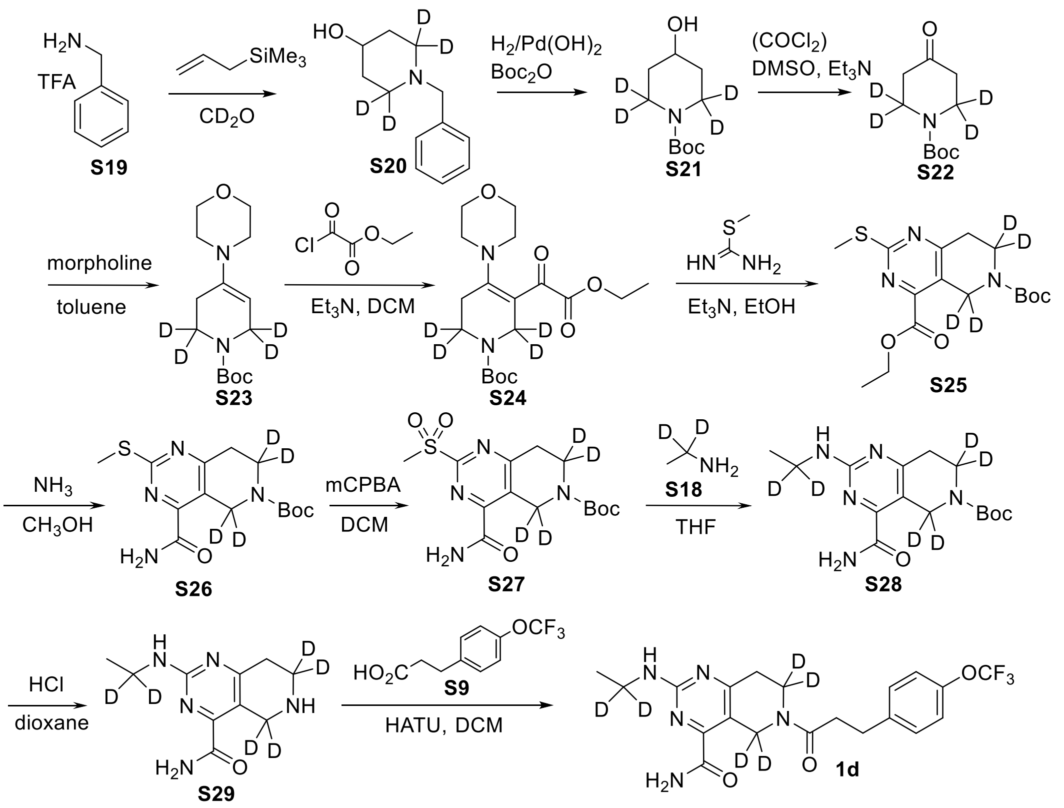

To a solution of benzylamine (10 g, 93 mmol) in dichloromethane (100 mL) was added trifluoroacetic acid (10.5 g, 93 mmol) dropwise at room temperature and the mixture was stirred for 1 h. The solid was filtered to give 19 g of a white solid **S19**. To a portion of this solid (7 g, ~32 mmol) was added a 20% solution of CD_2O (10 mL, 72 mmol) in D_2O and the mixture was sonicated for 10 min, and stirred at room temperature for 1 h. Allyltrimethylsilane (5.5 mL, 34 mmol) was added and the mixture was stirred at 40 °C. After 18 h, it was cooled to room temperature and diluted with water (10 mL) and the pH was adjusted to > pH 9 (as indicated by pH paper) with K_2CO_3. The mixture was extracted with ethyl acetate (3 x 30 mL), the combined organic layers were dried (Na_2SO_4), filtered and concentrated under reduced pressure to give an oil that was purified by silica gel column chromatography by eluting with

dichloromethane/methanol (10:1) to give compound **S20** (3.5 g, 57%) as a white solid. ^{1}H NMR ($CDCl_3$, 400 MHz) δ 7.32-7.28 (m, 5 H), 3.77-3.75 (m, 1 H), 3.62-3.48 (m, 2 H), 2.03-1.92 (m, 2 H), 1.65-1.62 (m, 2 H); exchangeable hydroxy proton not visible.

To a solution of compound **S20** (2.7 g, 14 mmol) and Boc_2O (3.6 g, 18 mmol) in methanol (30 mL) was added $Pd(OH)_2$ (0.65 g) and the mixture was hydrogenated in a Parr shaker at 55 psi for 24 h. The mixture was filtered through Celite® and the filtrate was concentrated under reduced pressure to give an oil that was purified via silica gel column chromatography eluting with dichloromethane/methanol (10:1) to give compound **S21** (2.2 g, 78%) as a colorless oil. ^{1}H NMR ($CDCl_3$, 400 MHz) δ 3.86-3.05 (m, 1 H), 1.85-1.81 (m, 2 H), 1.65-1.62 (m, 2 H), 1.45 (s, 9 H); exchangeable hydroxy proton not visible.

To a solution of oxalyl chloride (825 mg, 6.5 mmol) in dichloromethane (40 mL) was added DMSO (1 g, 12.9 mmol) at -78 °C, and the mixture was stirred for 1 h. Compound **S21** (1.0 g, 5.0 mmol) in dichloromethane (15 mL) was added at -78 °C and stirring was continued for 2 h. Triethylamine (3.0 g, 30 mmol) was added and the mixture was allowed to warm to room temperature while stirring for 30 min. The mixture was diluted with dichloromethane (20 mL) and washed with sat. aq. citric acid (40 mL). The organic layer was dried (Na_2SO_4), filtered and concentrated to give compound **S22** (1 g, >99%) as a yellow solid. ^{1}H NMR ($CDCl_3$, 400 MHz) δ 2.42 (s, 4 H), 1.48 (s, 9 H).

To a solution of compound **S22** (2.0 g, 9.8 mmol) in toluene (30 mL) was added morpholine (0.9 g, 10.8 mmol) and the flask was equipped with a Dean-Stark trap. The reaction mixture was heated under reflux for 18 h, whereupon it was cooled to room temperature and concentrated

under reduced pressure to give compound **S23** (2.6 g) as a yellow liquid. This material was used without further purification.

To a solution of compound **S23** (~2.6 g, ~9.5 mmol) in dichloromethane (15 mL) was added triethylamine (1.2 g, 11.7 mmol) and ethyloxalyl chloride (1.5 g, 11.0 mmol) dropwise at 0 °C, and the mixture was stirred at room temperature for 18 h. The mixture was filtered and the filtrate was concentrated under reduced pressure to give compound **S24** (4.5 g) as a yellow oil. This material was used without further purification.

To a solution of compound **S24** (~4.5 g, ~ 12 mmol) in ethanol (20 mL) was added S-methylthiourea (2.3 g, 12 mmol) and triethylamine (4.9 g, 48 mmol) and the mixture was heated under reflux for 18 h. The mixture was allowed to cool to room temperature and filtered to give a solid. The solid was washed with dichloromethane (20 mL) and the filtrate was concentrated under reduced pressure to give compound **S25** (0.7 g) as a white solid.

To a solution of compound **S25** (~0.7 g, 1.96 mmol) in methanol (15 mL) was added a solution of 7 N ammonia in methanol (15 mL, 105 mmol), and the mixture was stirred in a sealed bottle at 40 °C for 18 h. The mixture was cooled to room temperature and the solvent was removed under reduced pressure to give compound **S26** (~0.6 g) as a white solid. This material was used without further purification. ^{1}H NMR ($CDCl_3$, 400 MHz) δ 7.74 (br s, 1 H), 5.53 (br s, 1 H), 2.94 (s, 2 H), 2.54 (s, 3 H), 1.46 (s, 9 H).

To a solution of compound **S26** (~0.35 g, 1.06 mmol) in dichloromethane (7 mL) was added m-chloroperbenzoic acid (0.47 g, 2.5 mmol) in portions at 0 °C, and the mixture was allowed to warm to room temperature while stirring for 2 h. The reaction mixture was poured into a separatory funnel and washed with sat. aq. $Na_2S_2O_3$ (2 x 10 mL), sat. aq. K_2CO_3 (2 x 10 mL),

dried (Na_2SO_4), filtered and concentrated under reduced pressure to give compound **S27** (~300 mg) as a white solid. This material was used without further purification. ^1H NMR ($CDCl_3$, 400 MHz) δ 3.36 (s, 3 H), 3.16 (s, 2 H), 1.48 (s, 9 H).

To a solution of compound **S27** (~365 mg, ~1.0 mmol) in THF (5 mL) was added **S18** (d_2-ethylamine, 320 mg, 3.2 mmol), and the mixture was stirred at 80 °C for 18 h. The solvent was removed under reduced pressure and the residue was purified by a preparative TLC (petroleum ether/ethylacetate) to give compound **S28** (110 mg) as a colorless oil.

To a solution of compound **S28** (110 mg) in dichloromethane (5 mL) was added 4.0 M HCl in dioxane (5 mL) dropwise at 0 °C, and the mixture was stirred at room temperature for 2 h. The mixture was filtered to give a solid that was further dried in a vacuum oven to give compound **S29** (105 mg, >99%) as a yellow solid

To a solution of compound **S29** (100 mg, 0.4 mmol) in dichloromethane (5 mL) was added 4-trifluoromethoxyhydrocinnamic acid (**S9**, 98 mg, 0.4 mmol), triethylamine (112 mg, 1.1 mmol) and HATU (174 mg, 0.5 mmol), and the mixture was stirred at room temperature for 15 h. The solvent was removed under reduced pressure and the residue was purified via preparative HPLC to give the compound **1d** (75 mg, 45%) as a white solid. ^1H NMR ($CDCl_3$, 400 MHz) δ 7.84 (br s, 1 H), 7.19 (d, J = 8.0 Hz, 2 H), 7.01 (d, J = 8.0 Hz, 2 H), 5.47 (br s, 1 H), 5.03 (br s, 1 H), 3.01-2.94 (m, 2 H), 2.77-2.67 (m, 4 H), 1.29 (s, 3 H); m/z = 444.1$(M+H)^+$.

Fig F.

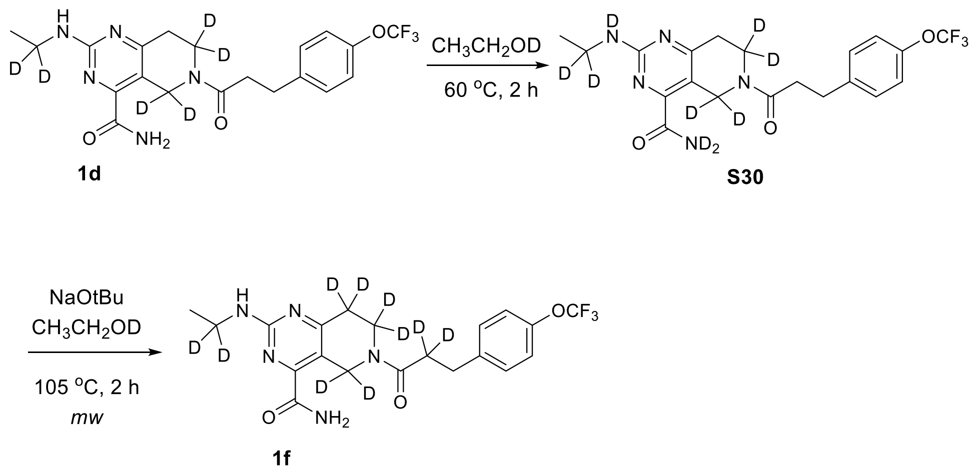

To a microwave vial was added compound **1d** (100 mg, 0.22 mmol) in deuterated ethanol (CH_3CH_2OD, 2 mL). The solution was heated at 60 °C for 2 h under N_2 atmosphere. The solvent was removed under reduced pressure to provide crude **S30**. The residue was dissolved in deuterated ethanol (CH_3CH_2OD, 4 mL) and NaOtBu (5 mg, 0.05 mmol) was added. The resulting mixture was heated in a microwave reactor (200 W) at 105 °C for 2 h. The solvent was removed under reduced pressure to give a residue, to which was added additional portion of NaOtBu (5 mg, 0.05 mmol) and deuterated ethanol (CH_3CH_2OD, 2 mL). The resulting mixture was heated in a microwave (200 W) at 105 °C for 1.5 h. The solvent was removed under reduced pressure to give a crude product, which was purified via silica gel column chromatography eluting with EtOAc/hexane gradient to give **1f** as white solid (85 mg, 85%). ^{1}H NMR (400 MHz, $CDCl_3$) δ 7.12 (d, J = 8.6 Hz, 2 H), 6.94 (d, J = 8.1 Hz, 2 H), 2.95 (s, 2 H), 1.27 (s, 3 H); ^{13}C NMR (100 MHz, $CDCl_3$): 170.9, 168.1, 167.3, 150.9, 147.5, 139.6, 129.9, 120.9, 120.5 (q, $^1J_{CF}$ = 256 Hz) , 119.1, 115.4, 44.0 (m), 41.9, 35.8(m), 34.6 (m), 30.8, 29.8 (m), 14.5. m/z = 448.4 $(M+H)^+$. HRMS Calcd. for $C_{20}H_{13}D_{10}F_3N_5O_3$ $(M+H)^+$ 448.2375; Found 448.2367.

Summary of spectral data for deuterated triazole compounds (2a-d)

Fig G.

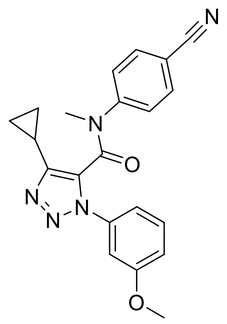

^{1}H NMR (400 MHz, CD_3OD) δ 7.51 (d, *J* = 8.4 Hz, 2 H), 7.44-7.40 (m, 1 H), 7.01 (dd, *J* = 8.2, 2.3 Hz, 1 H), 6.87-6.78 (m, 3 H), 6.68 (br s, 1 H), 3.82 (s, 3 H), 3.39 (s, 3 H), 2.03-2.00 (m, 1 H), 1.04-0.80 (m, 4 H); m/z 374.2 9 $(M+1)^{+}$.

Fig H.

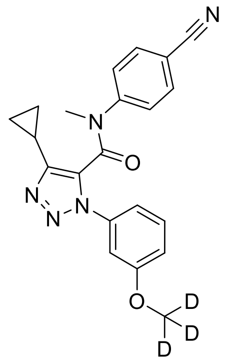

^{1}H NMR (CD_3OD, 400 MHz) δ 7.53 (d, *J* = 8.4 Hz, 2 H), 7.44 (t, *J* = 8.4 Hz, 1 H), 7.12 (d, *J* = 5.6 Hz, 1 H), 6.84-6.82 (m, 3 H), 6.71 (s, 1 H), 3.43 (s, 3 H), 2.05 (p, *J* = 7.6 Hz, 1 H), 1.07-0.75 (m, 4 H); ^{13}C NMR (CD_3OD, 100.5 MHz) δ 162.2, 162.0, 151.3, 147.0, 138.4, 134.3, 131.8, 130.3, 127.5, 119.0, 116.9, 116.1, 111.9, 109.9, 37.6, 8.5, 7.1; signal for expected septet for CD_3-

N at ~ 55 ppm not above baseline. m/z = 377.0 $(M+H)^+$. HRMS Calcd. for $C_{21}H_{17}D_3N_5O_2$ $(M+H)^+$ 377.1800; Found 377.1798.

Fig I.

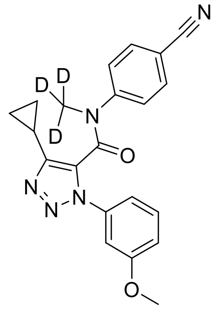

^1H NMR (CD_3OD, 400 MHz) δ 7.53 (d, *J* = 8.4 Hz, 2 H), 7.44 (t, *J* = 8.4 Hz, 1 H), 7.12 (dd, *J* = 8.4, 2.4 Hz, 1 H), 6.84-6.82 (m, 3 H), 6.71 (s, 1 H), 3.84 (s, 3 H), 2.05 (p, *J* = 7.6 Hz, 1 H), 1.20-0.85 (m, 4 H); ^{13}C NMR (CD_3OD, 100.5 MHZ) δ 160.7, 160.4, 149.7, 145.5, 136.9, 132.8, 130.3, 128.8, 126.0, 117.4, 115.4, 114.6, 110.4, 108.4, 54.8, 7.0, 5.5; signal for expected septet for $-OCD_3$ at ~ 36 ppm not above baseline. m/z = 377.2 $(M+H)^+$. HRMS Calcd. for $C_{21}H_{17}D_3N_5O_2$ $(M+H)^+$ 377.1800; Found 377.1798.

Fig J.

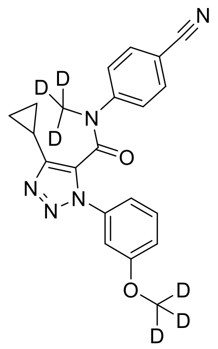

^{1}H NMR ($CDCl_3$, 400 MHz) δ 7.35 (d, *J* = 8.4 Hz, 2 H), 7.30 (t, *J* = 8.4 Hz, 1 H), 6.96 (dd, *J* = 8.4, 2.4 Hz, 1 H), 6.76 (d, *J* = 8.4 Hz, 1 H), 6.69-6.55 (m, 2 H), 1.99 (pentet, *J* = 7.6 Hz, 1 H), 1.05-0.95 (m, 4 H); ^{13}C NMR ($CDCl_3$, 100.5 MHz) δ 160.6, 160.4, 150.3, 145.5, 137.1, 132.8, 130.1, 128.2, 125.6, 117.6, 115.7, 114.7, 110.4, 108.1, 54.8 (septet, J = 22.7 Hz), 36.8 (partially resolved septet, J = 26.4 Hz), 8.0, 6.2. m/z = 380.3 $(M+H)^+$. HRMS Calcd. for $C_{21}H_{14}D_6N_5O_2$ $(M+H)^+$ 380.1988; Found 380.1984.

Fig K.

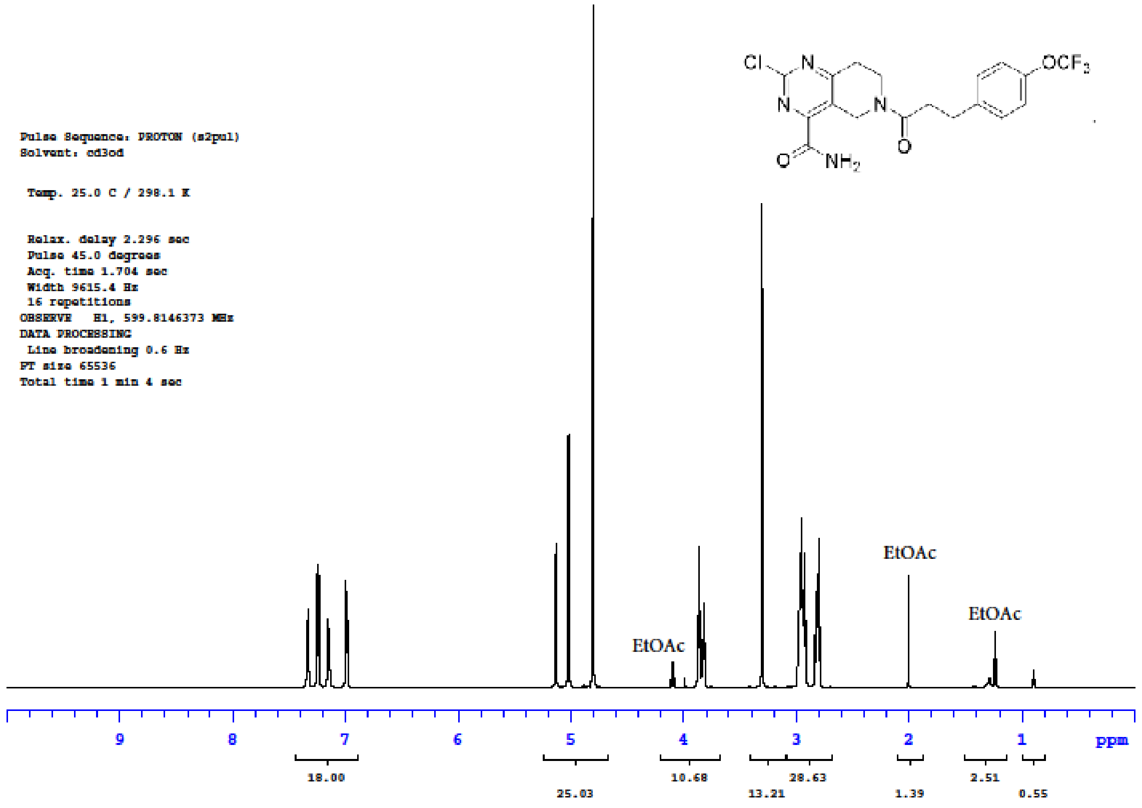
Pulse Sequence: PROTON (s2pul)
Solvent: cd3od
Temp. 25.0 C / 298.1 K
Relax. delay 2.296 sec
Pulse 45.0 degrees
Acq. time 1.704 sec
Width 9615.4 Hz
16 repetitions
OBSERVE H1, 599.8146373 MHz
DATA PROCESSING
Line broadening 0.6 Hz
FT size 65536
Total time 1 min 4 sec
Cl
N
N
N
O
NH2
O
OCF3
EtOAc
EtOAc
EtOAc
9
8
7
6
5
4
3
2
1
ppm
18.00
25.03
10.68
13.21
28.63
1.39
2.51
0.55

Fig L.

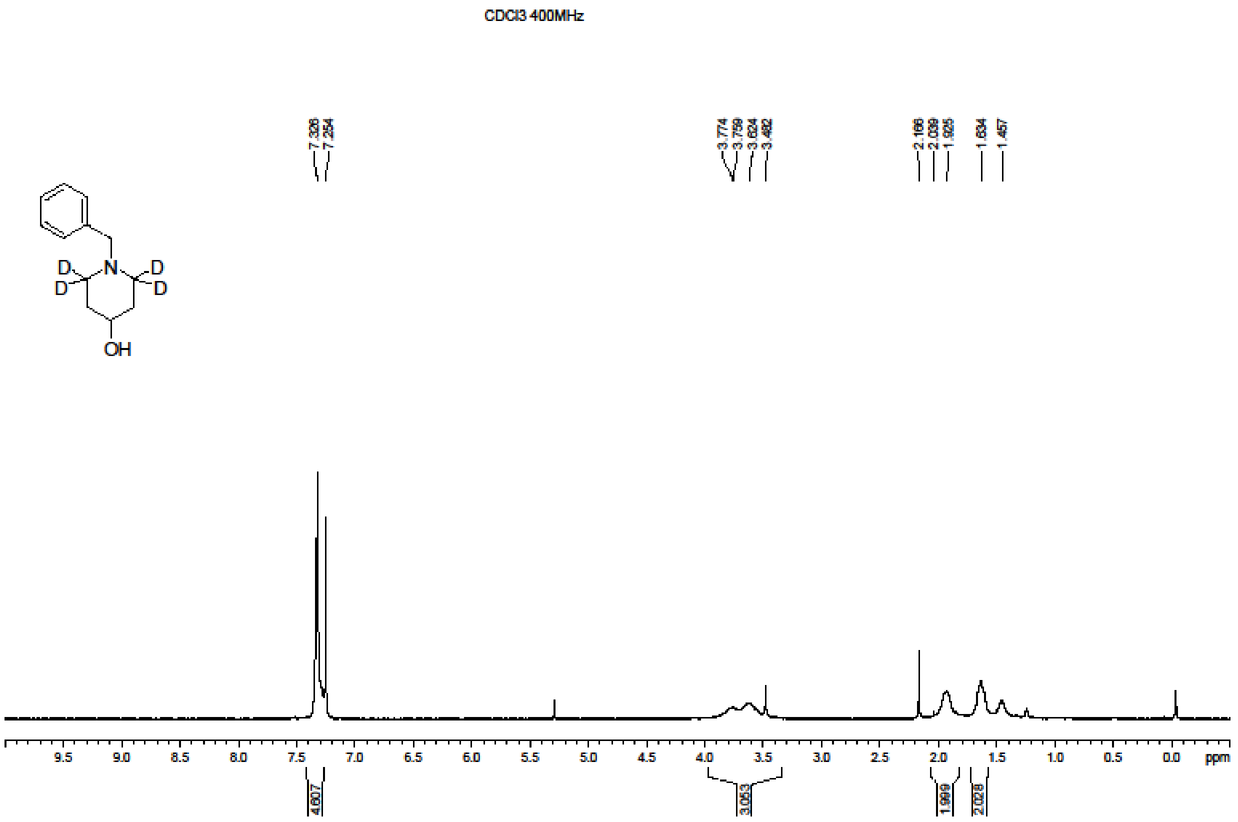
CDCl3 400MHz
7.326
7.254
3.774
3.759
3.624
3.482
2.166
2.039
1.925
1.634
1.457
N
D
D
D
D
OH
9.5
9.0
8.5
8.0
7.5
7.0
6.5
6.0
5.5
5.0
4.5
4.0
3.5
3.0
2.5
2.0
1.5
1.0
0.5
0.0
ppm
4.607
3.053
1.999
2.028

Fig M.

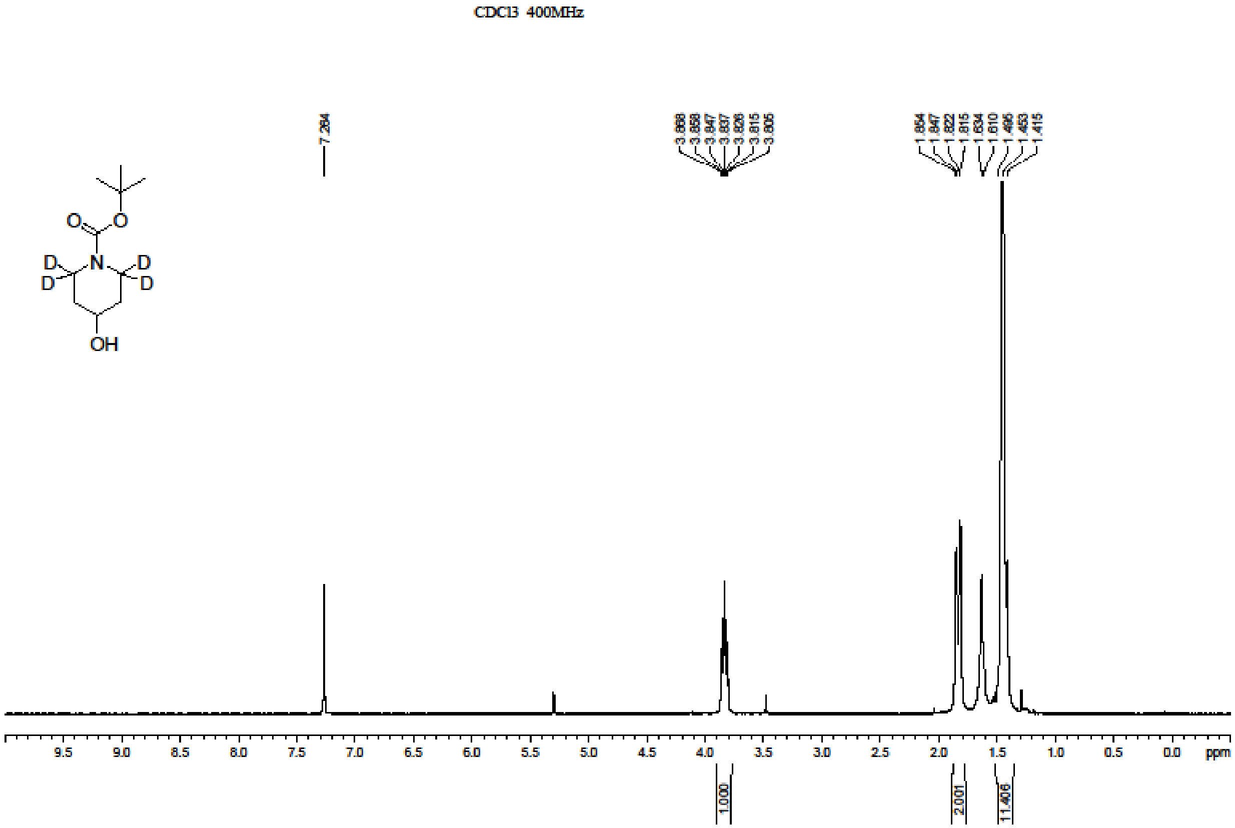
CDCl3 400MHz
7.264
3.868
3.858
3.847
3.837
3.826
3.815
3.805
1.854
1.847
1.822
1.815
1.634
1.610
1.495
1.453
1.415
9.5
9.0
8.5
8.0
7.5
7.0
6.5
6.0
5.5
5.0
4.5
4.0
3.5
3.0
2.5
2.0
1.5
1.0
0.5
0.0
ppm
1.000
2.001
11.406
O
O
N
D
D
D
D
OH

Fig N.

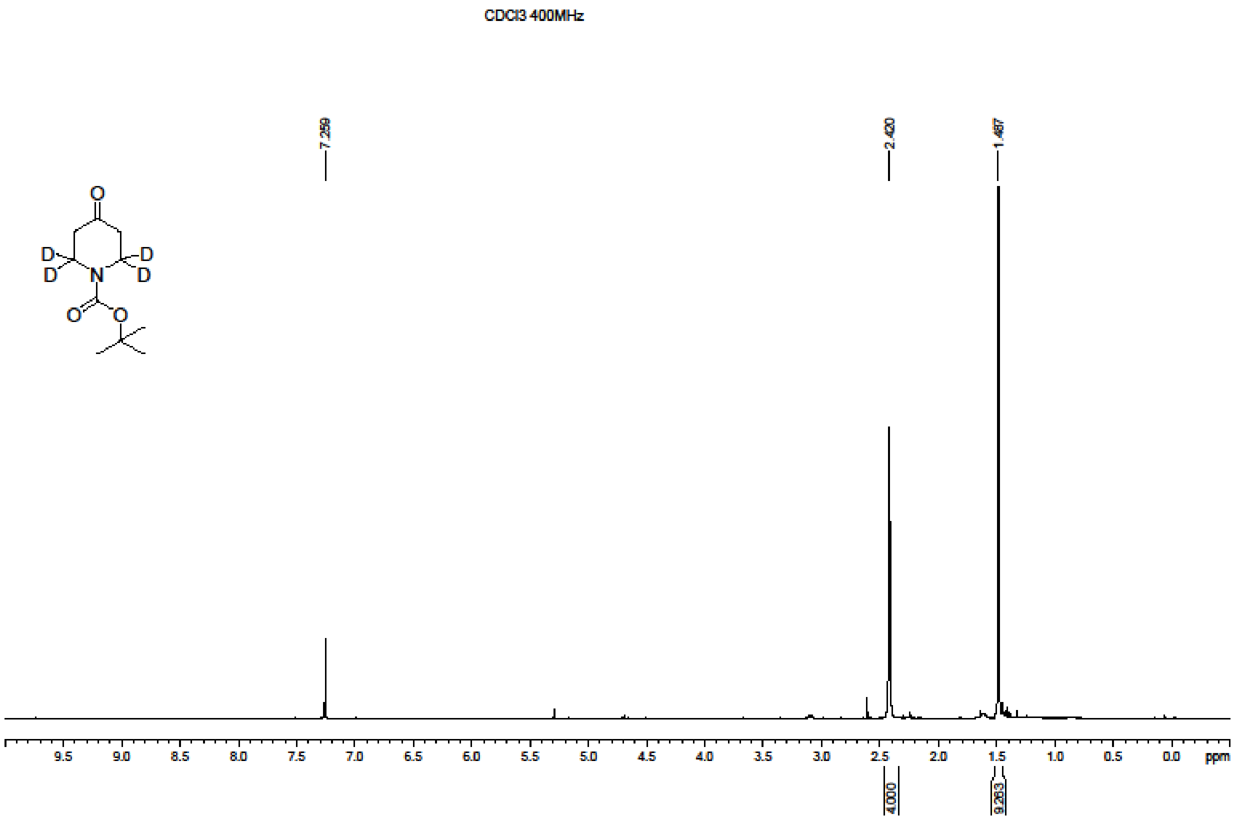
CDCl3 400MHz
7.259
2.420
1.467
9.5
9.0
8.5
8.0
7.5
7.0
6.5
6.0
5.5
5.0
4.5
4.0
3.5
3.0
2.5
2.0
1.5
1.0
0.5
0.0
ppm
4.000
9.263
O
D
D
N
D
D
O
O

Fig O.

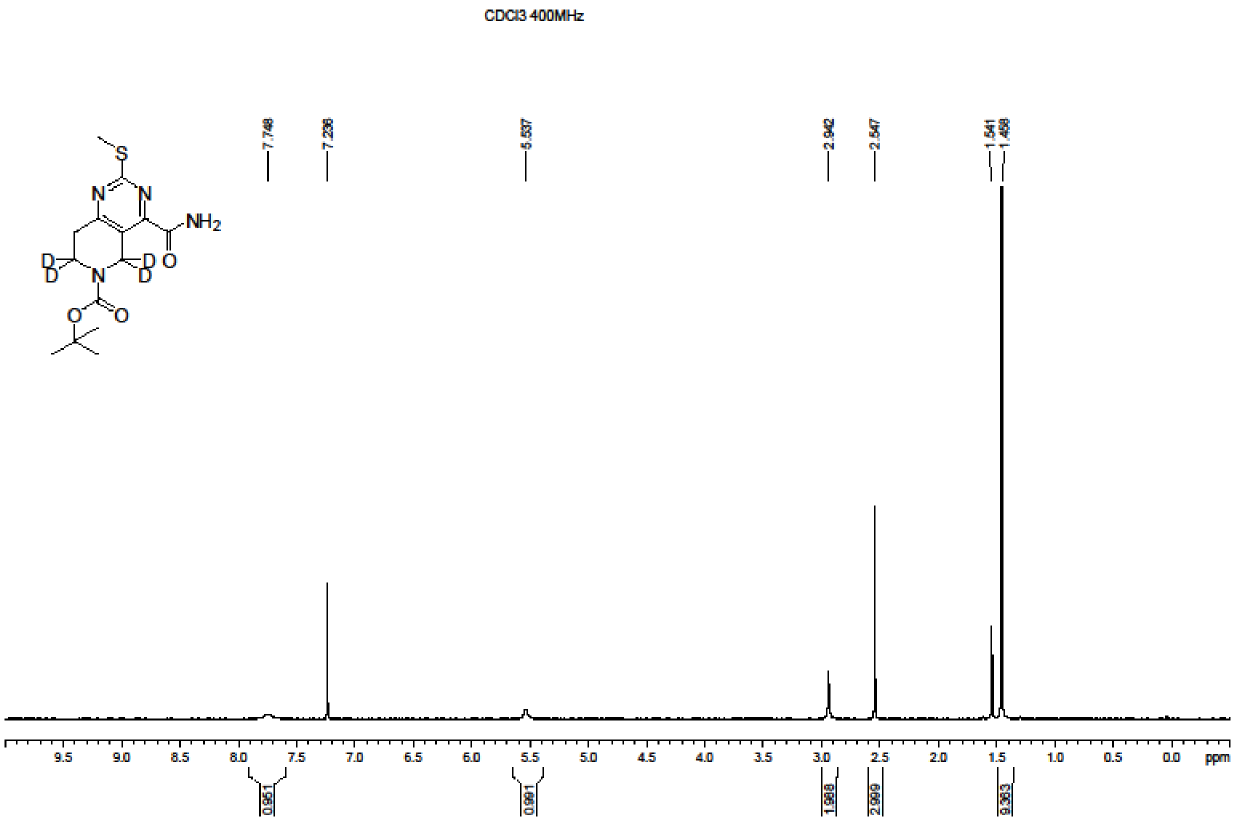
CDCl3 400MHz
7.748
7.236
5.537
2.942
2.547
1.541
1.458
9.5
9.0
8.5
8.0
7.5
7.0
6.5
6.0
5.5
5.0
4.5
4.0
3.5
3.0
2.5
2.0
1.5
1.0
0.5
0.0
ppm
0.951
0.991
1.988
2.999
9.363
S
N
N
NH2
O
D
D
D
D
N
O
O

Fig P.

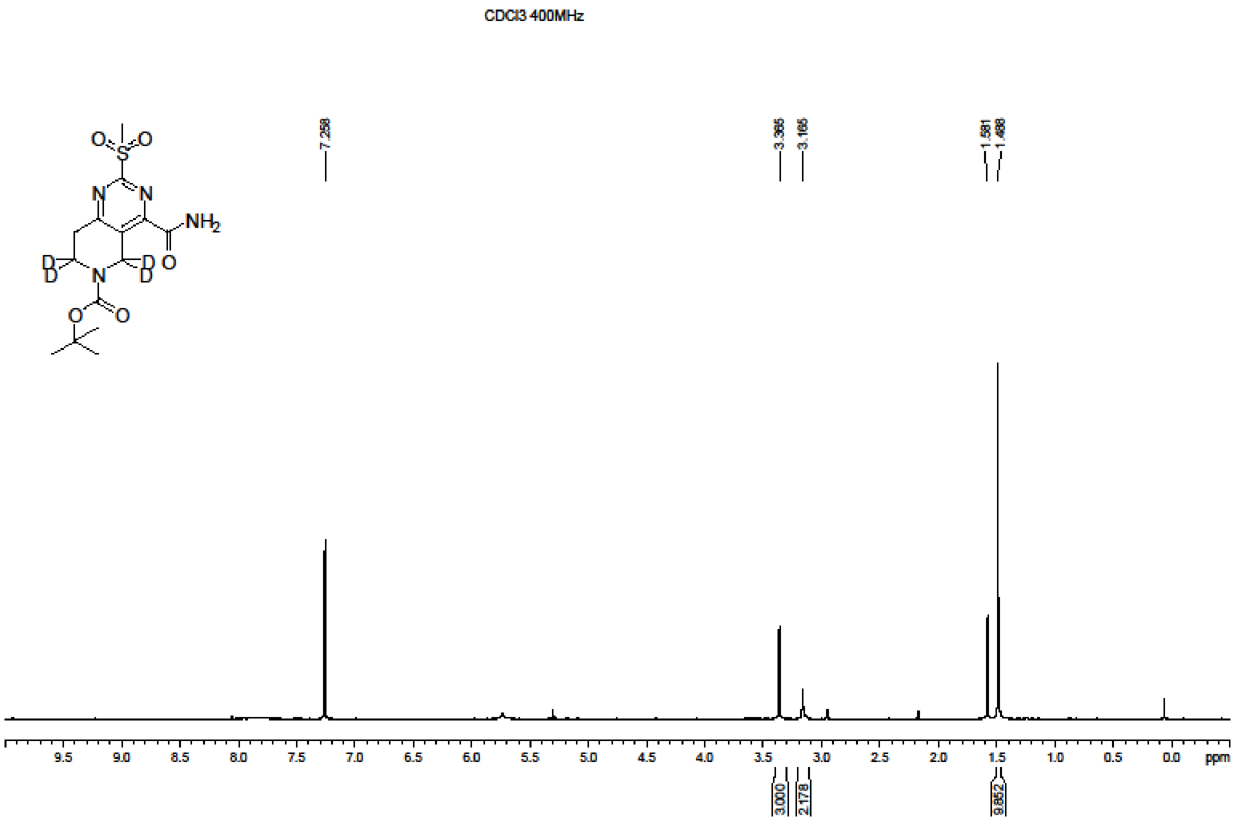
CDCl3 400MHz
7.258
3.365
3.165
1.581
1.498
9.5
9.0
8.5
8.0
7.5
7.0
6.5
6.0
5.5
5.0
4.5
4.0
3.5
3.0
2.5
2.0
1.5
1.0
0.5
0.0
ppm
3.000
2.178
9.852
NH2
D
N
O
S

Fig Q.

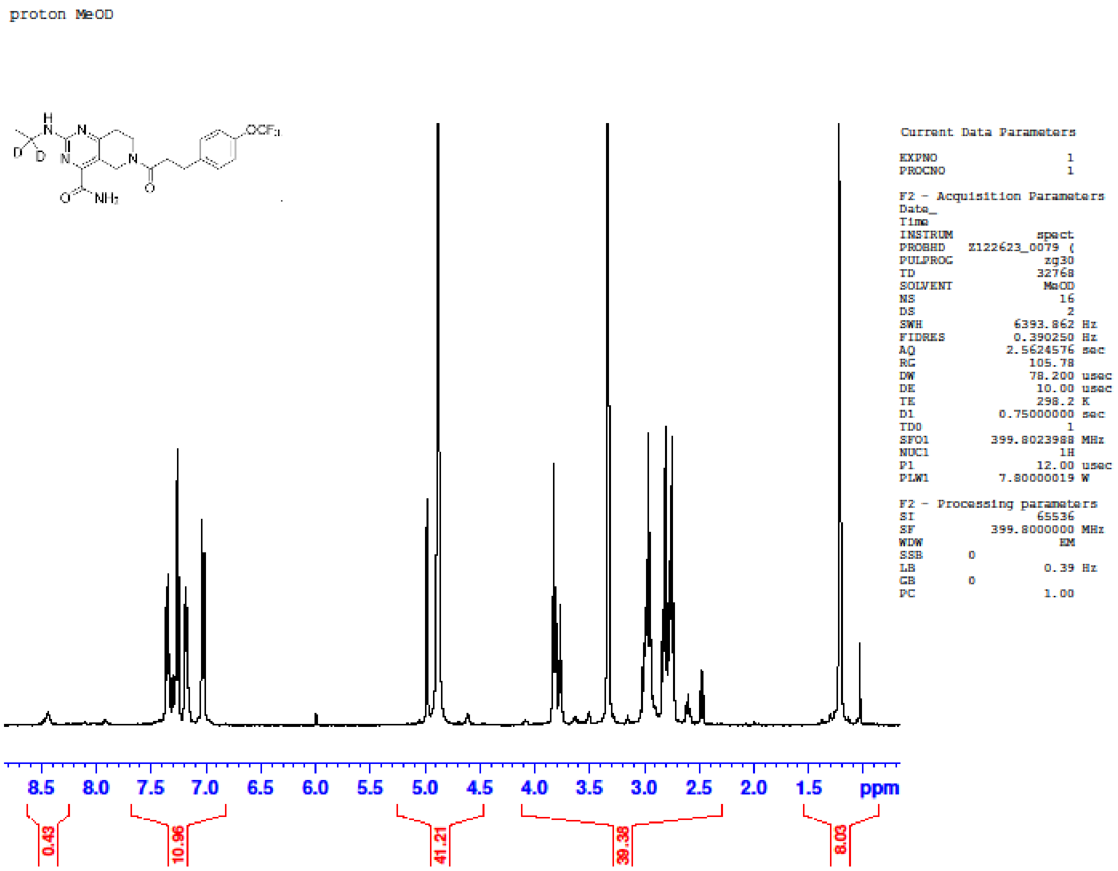

proton MeOD
OCF3
NH2
Current Data Parameters
EXPNO 1
PROCNO 1
F2 - Acquisition Parameters
Date_
Time
INSTRUM spect
PROBHD Z122623_0079 (
PULPROG zg30
TD 32768
SOLVENT MeOD
NS 16
DS 2
SWH 6393.862 Hz
FIDRES 0.390250 Hz
AQ 2.5624576 sec
RG 105.78
DW 78.200 usec
DE 10.00 usec
TE 298.2 K
D1 0.75000000 sec
TD0 1
SFO1 399.8023988 MHz
NUC1 1H
P1 12.00 usec
PLW1 7.80000019 W
F2 - Processing parameters
SI 65536
SF 399.8000000 MHz
WDW EM
SSB 0
LB 0.39 Hz
GB 0
PC 1.00
8.5 8.0 7.5 7.0 6.5 6.0 5.5 5.0 4.5 4.0 3.5 3.0 2.5 2.0 1.5 ppm
0.43
10.96
41.21
39.38
8.03

Fig R.

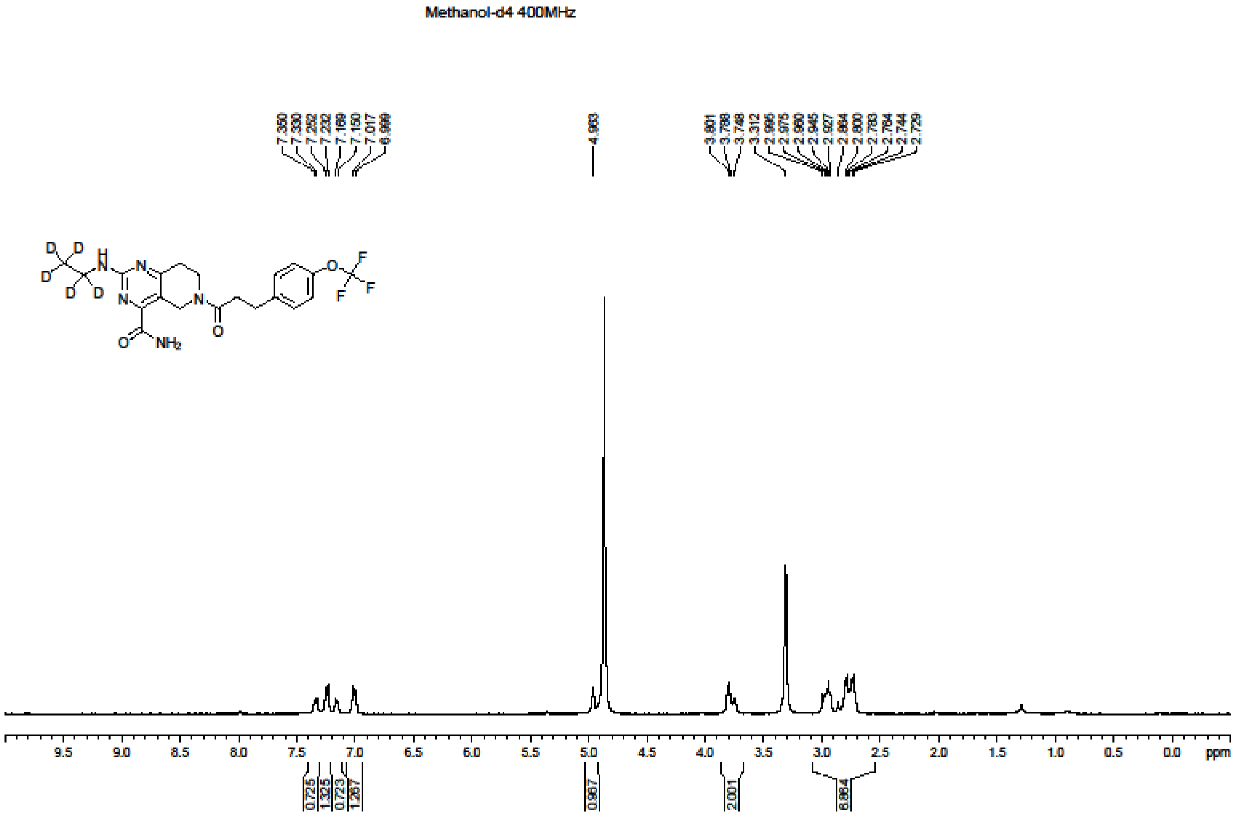

Fig S.

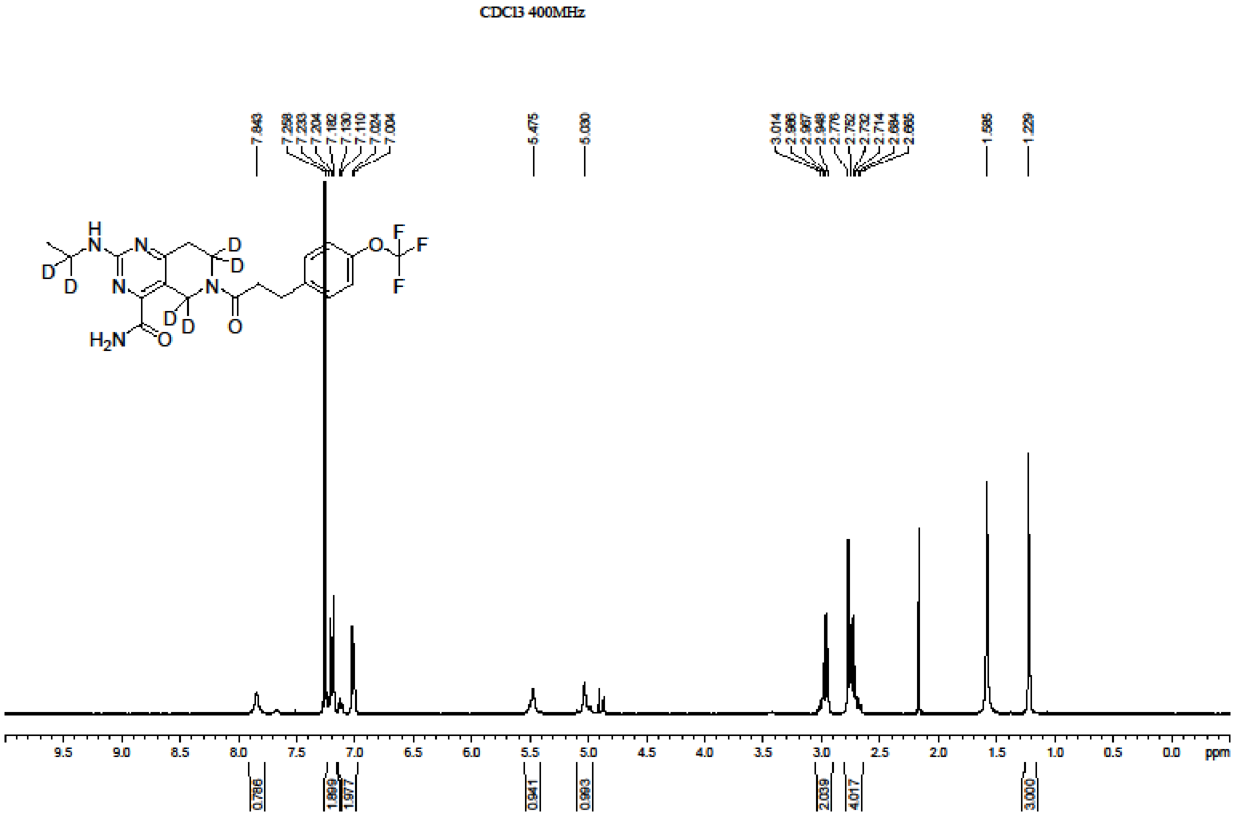

Fig T.

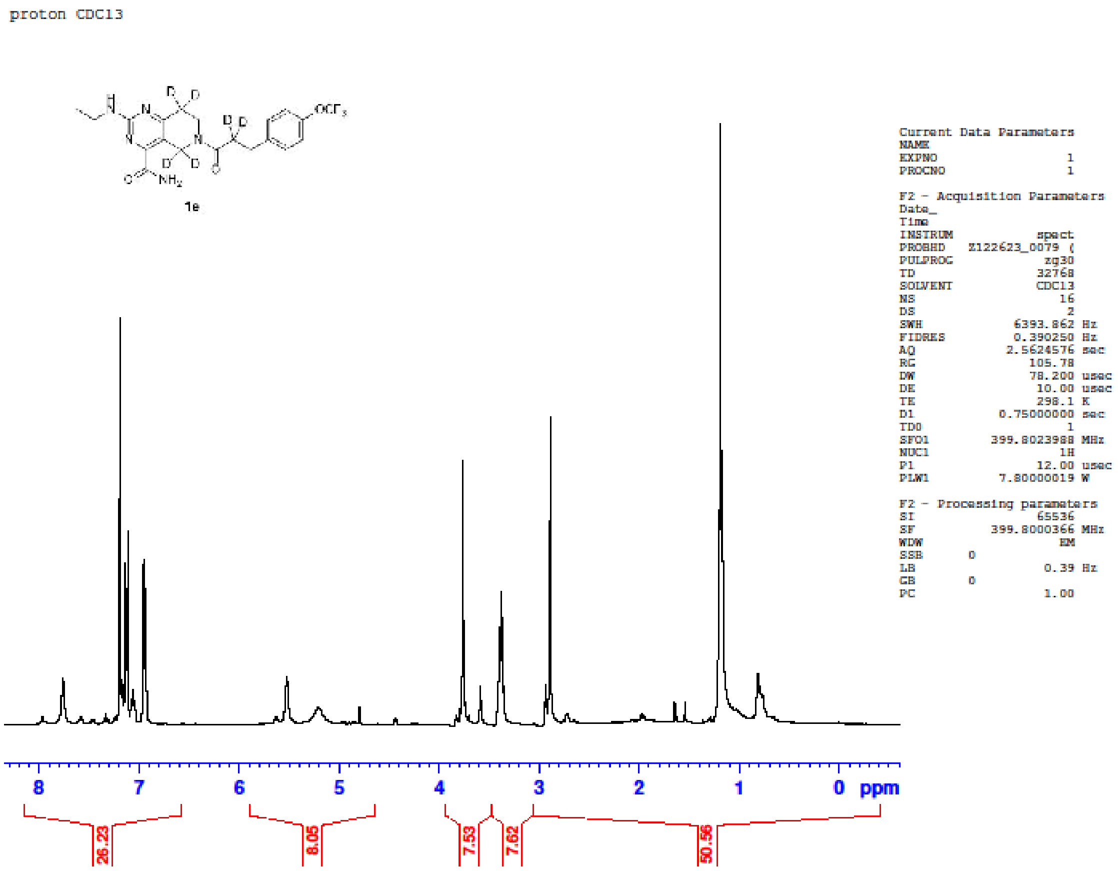
proton CDCl3
OCF3
NH2
1e
Current Data Parameters
NAME
EXPNO 1
PROCNO 1
F2 - Acquisition Parameters
Date_
Time
INSTRUM spect
PROBHD Z122623_0079 (
PULPROG zg30
TD 32768
SOLVENT CDCl3
NS 16
DS 2
SWH 6393.862 Hz
FIDRES 0.390250 Hz
AQ 2.5624576 sec
RG 105.78
DW 78.200 usec
DE 10.00 usec
TE 298.1 K
D1 0.75000000 sec
TD0 1
SFO1 399.8023988 MHz
NUC1 1H
P1 12.00 usec
PLW1 7.80000019 W
F2 - Processing parameters
SI 65536
SF 399.8000366 MHz
WDW EM
SSB 0
LB 0.39 Hz
GB 0
PC 1.00
8
7
6
5
4
3
2
1
0
ppm
26.23
8.05
7.53
7.62
50.56

Fig U.

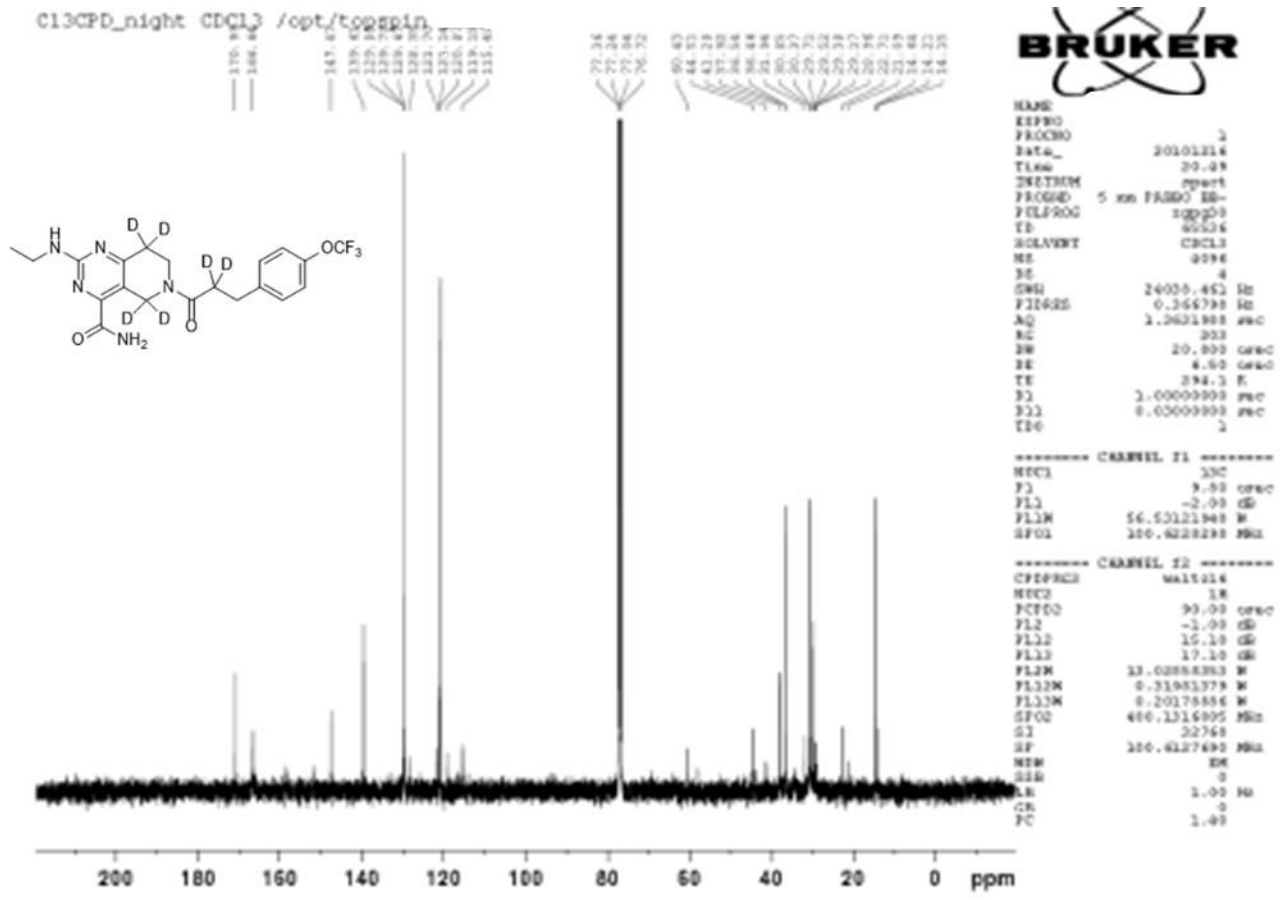

C13CPD_night CDCl3 /opt/topspin
BRUKER
ppm
200
180
160
140
120
100
80
60
40
20
0

Fig V.

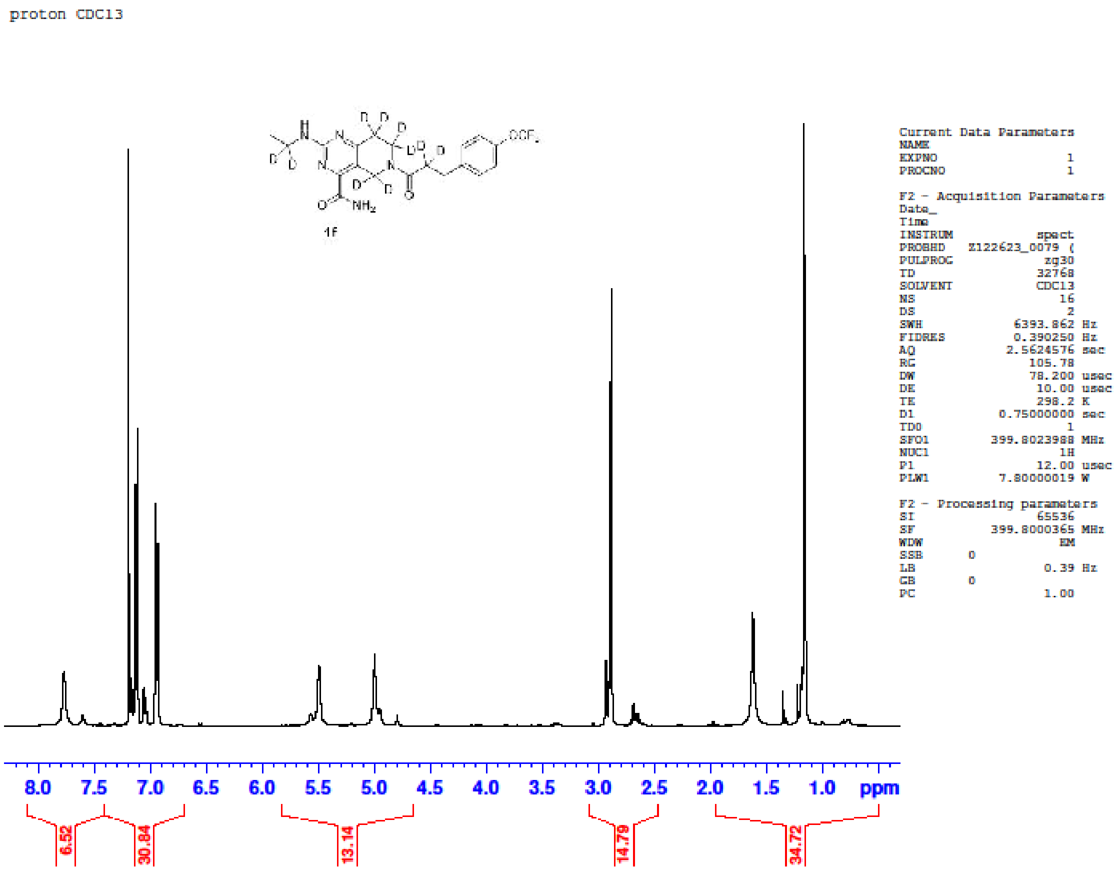
proton CDC13
1f
Current Data Parameters
NAME
EXPNO 1
PROCNO 1
F2 - Acquisition Parameters
Date_
Time
INSTRUM spect
PROBHD Z122623_0079 (
PULPROG zg30
TD 32768
SOLVENT CDC13
NS 16
DS 2
SWH 6393.862 Hz
FIDRES 0.390250 Hz
AQ 2.5624576 sec
RG 105.78
DW 78.200 usec
DE 10.00 usec
TE 298.2 K
D1 0.75000000 sec
TD0 1
SFO1 399.8023988 MHz
NUC1 1H
P1 12.00 usec
PLW1 7.80000019 W
F2 - Processing parameters
SI 65536
SF 399.8000365 MHz
WDW EM
SSB 0
LB 0.39 Hz
GB 0
PC 1.00
8.0 7.5 7.0 6.5 6.0 5.5 5.0 4.5 4.0 3.5 3.0 2.5 2.0 1.5 1.0 ppm
6.52
30.84
13.14
14.79
34.72

Fig W.

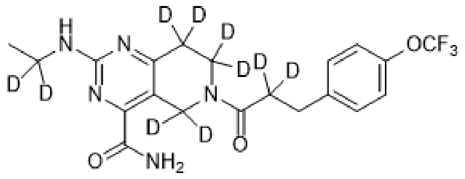
OCF3
NH2

1f

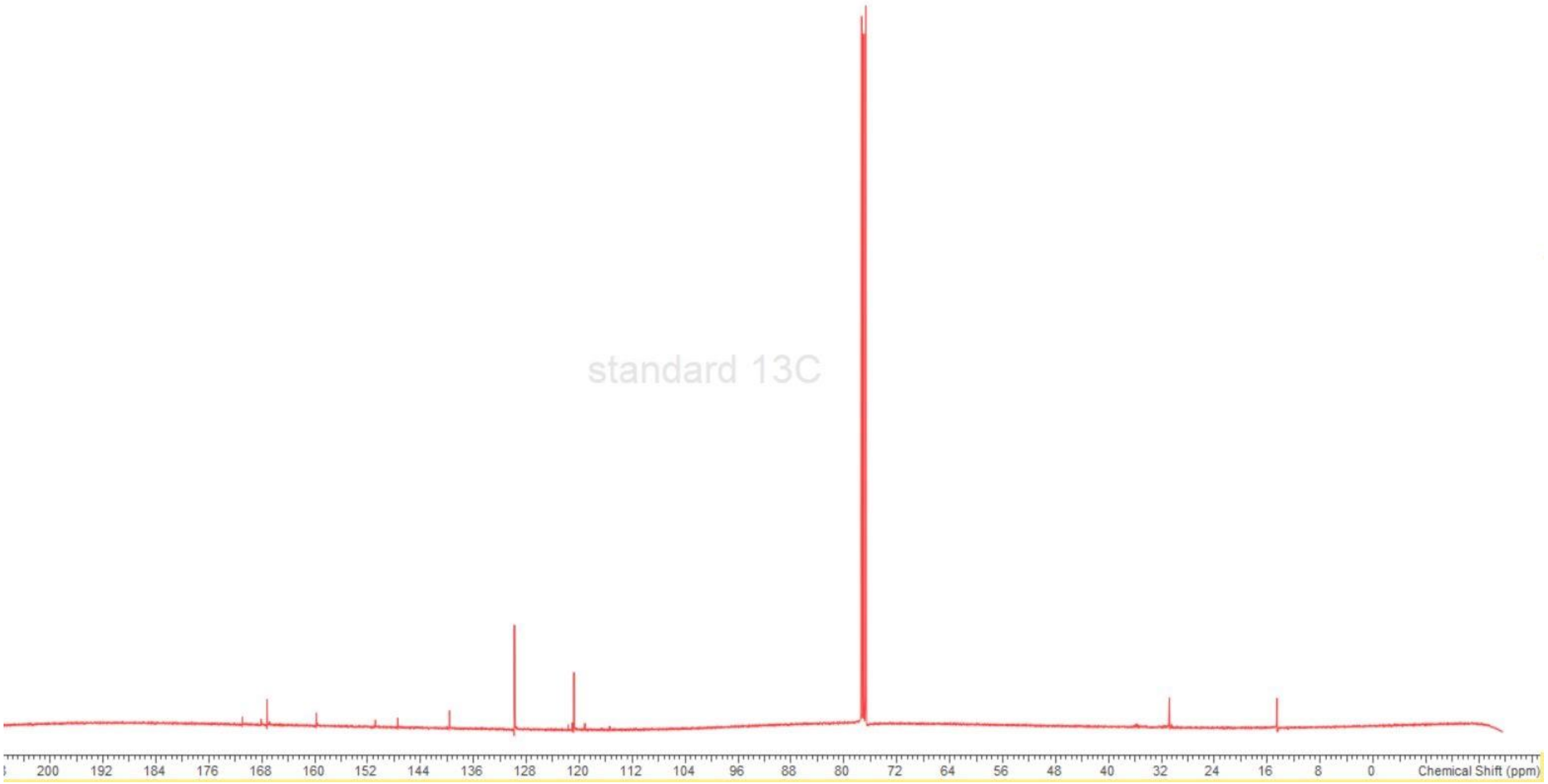
standard 13C
200
192
184
176
168
160
152
144
136
128
120
112
104
96
88
80
72
64
56
48
40
32
24
16
8
0
Chemical Shift (ppm)

Fig X.

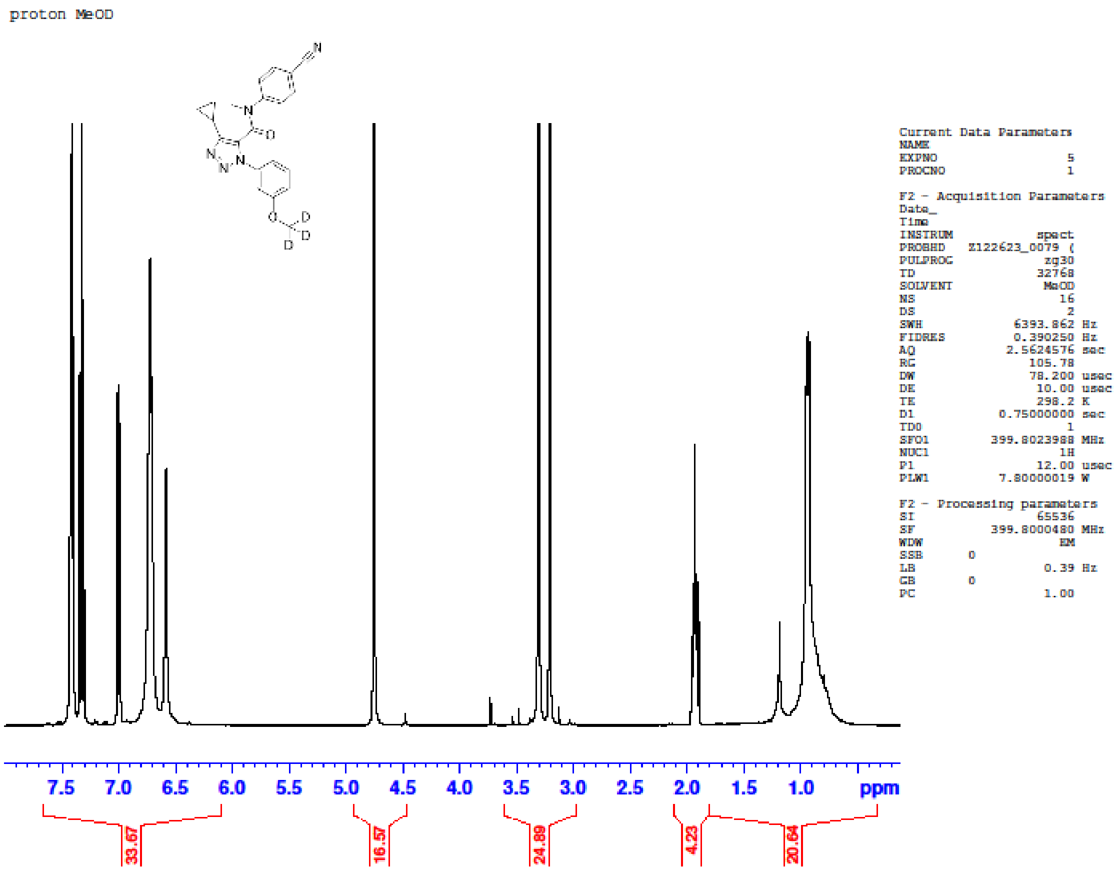

proton MeOD
N
N
O
N
N
N
O
D
D
D
Current Data Parameters
NAME
EXPNO 5
PROCNO 1
F2 - Acquisition Parameters
Date_
Time
INSTRUM spect
PROBHD Z122623_0079 (
PULPROG zg30
TD 32768
SOLVENT MeOD
NS 16
DS 2
SWH 6393.862 Hz
FIDRES 0.390250 Hz
AQ 2.5624576 sec
RG 105.78
DW 78.200 usec
DE 10.00 usec
TE 298.2 K
D1 0.75000000 sec
TD0 1
SFO1 399.8023988 MHz
NUC1 1H
P1 12.00 usec
PLW1 7.80000019 W
F2 - Processing parameters
SI 65536
SF 399.8000480 MHz
WDW EM
SSB 0
LB 0.39 Hz
GB 0
PC 1.00
7.5
7.0
6.5
6.0
5.5
5.0
4.5
4.0
3.5
3.0
2.5
2.0
1.5
1.0
ppm
33.67
16.57
24.89
4.23
20.64

Fig Y.

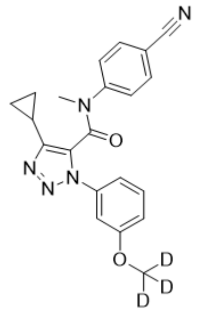
N
N
N
N
O
N
O
D
D
D

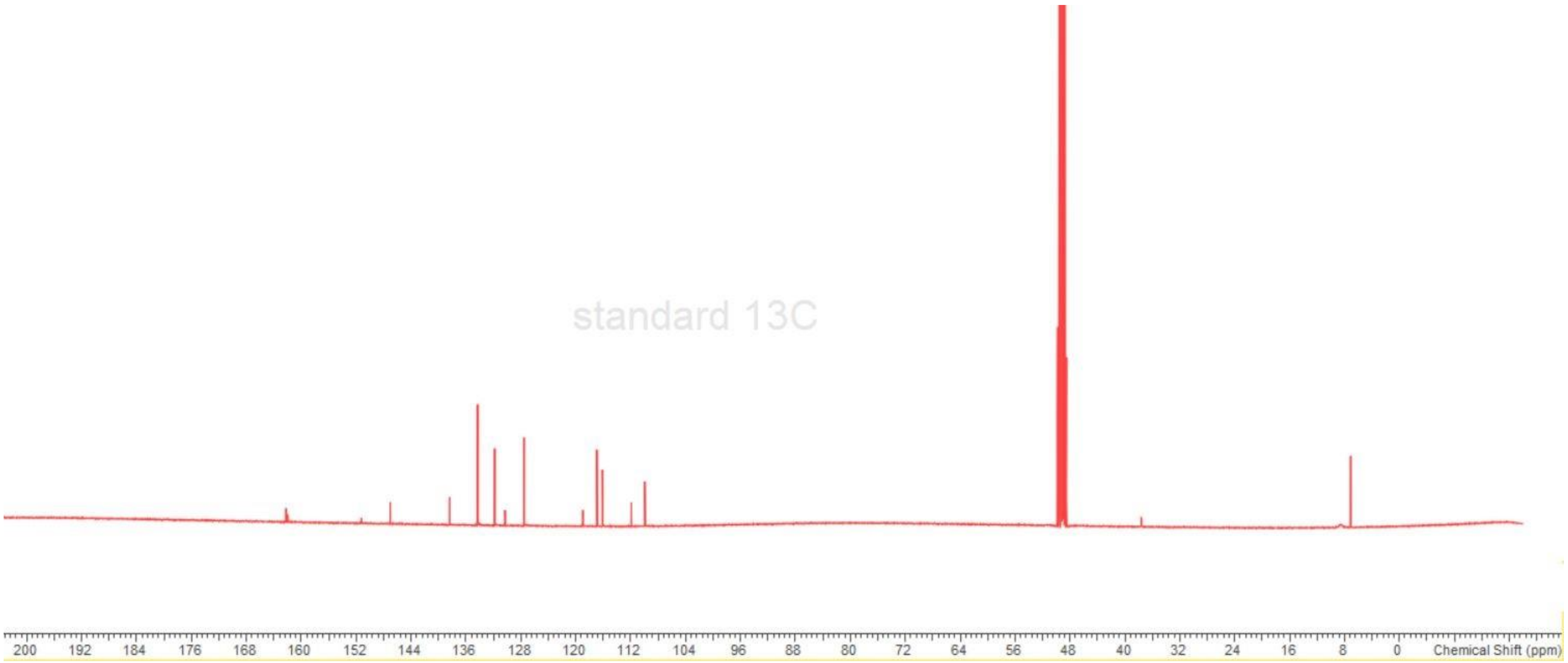
standard 13C
200
192
184
176
168
160
152
144
136
128
120
112
104
96
88
80
72
64
56
48
40
32
24
16
8
0
Chemical Shift (ppm)

Fig Z.

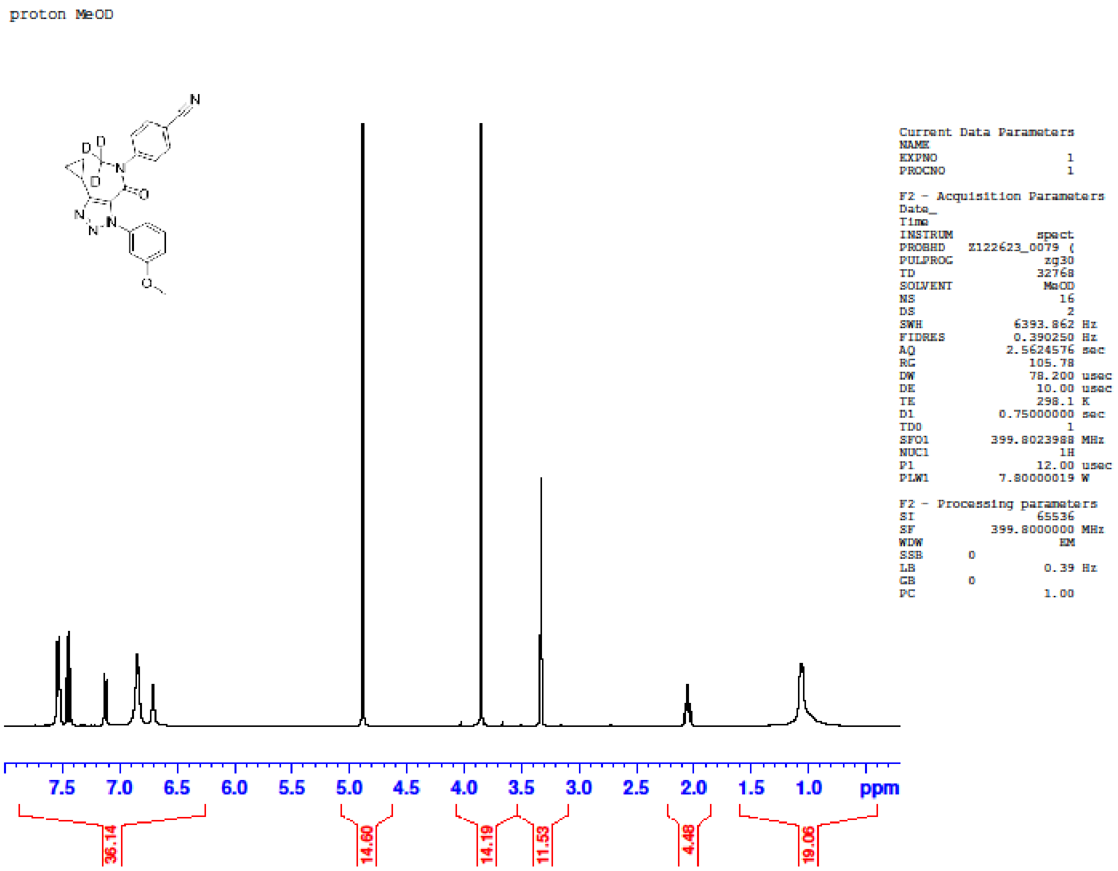
proton MeOD
N
D
D
D
N
O
N
N
N
O
Current Data Parameters
NAME
EXPNO 1
PROCNO 1
F2 - Acquisition Parameters
Date_
Time
INSTRUM spect
PROBHD Z122623_0079 (
PULPROG zg30
TD 32768
SOLVENT MeOD
NS 16
DS 2
SWH 6393.862 Hz
FIDRES 0.390250 Hz
AQ 2.5624576 sec
RG 105.78
DW 78.200 usec
DE 10.00 usec
TE 298.1 K
D1 0.75000000 sec
TD0 1
SFO1 399.8023988 MHz
NUC1 1H
P1 12.00 usec
PLW1 7.80000019 W
F2 - Processing parameters
SI 65536
SF 399.8000000 MHz
WDW EM
SSB 0
LB 0.39 Hz
GB 0
PC 1.00
7.5 7.0 6.5 6.0 5.5 5.0 4.5 4.0 3.5 3.0 2.5 2.0 1.5 1.0 ppm
36.14
14.60
14.19
11.53
4.48
19.06

Fig AA.

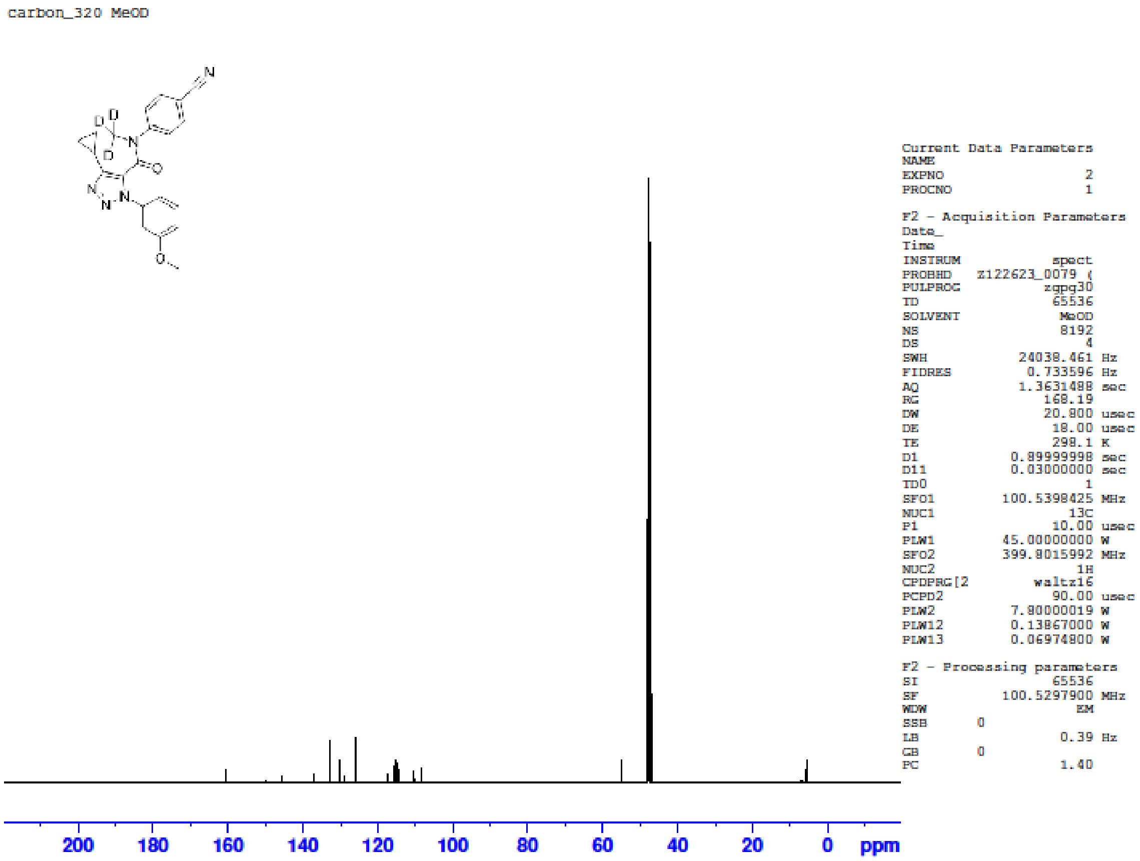
carbon_320 MeOD
N
D
D
D
D
N
O
N
N
N
O
Current Data Parameters
NAME
EXPNO 2
PROCNO 1
F2 - Acquisition Parameters
Date_
Time
INSTRUM spect
PROBHD Z122623_0079 (
PULPROG zgpg30
TD 65536
SOLVENT MeOD
NS 8192
DS 4
SWH 24038.461 Hz
FIDRES 0.733596 Hz
AQ 1.3631488 sec
RG 168.19
DW 20.800 usec
DE 18.00 usec
TE 298.1 K
D1 0.89999998 sec
D11 0.03000000 sec
TD0 1
SFO1 100.5398425 MHz
NUC1 13C
P1 10.00 usec
PLW1 45.00000000 W
SFO2 399.8015992 MHz
NUC2 1H
CPDPRG[2 waltz16
PCPD2 90.00 usec
PLW2 7.80000019 W
PLW12 0.13867000 W
PLW13 0.06974800 W
F2 - Processing parameters
SI 65536
SF 100.5297900 MHz
WDW EM
SSB 0
LB 0.39 Hz
GB 0
PC 1.40
200
180
160
140
120
100
80
60
40
20
0
ppm

Fig AB.

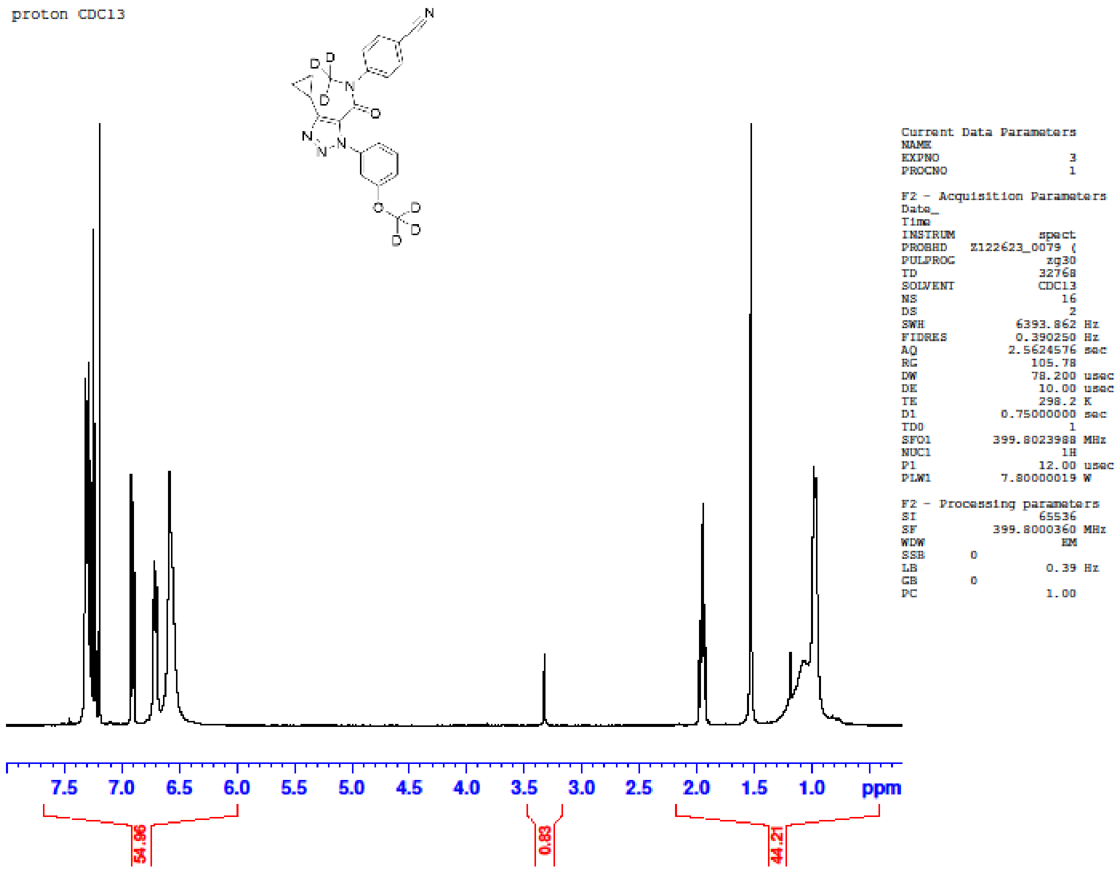
proton CDCl3
Current Data Parameters
NAME
EXPNO 3
PROCNO 1
F2 - Acquisition Parameters
Date_
Time
INSTRUM spect
PROBHD Z122623_0079 (
PULPROG zg30
TD 32768
SOLVENT CDCl3
NS 16
DS 2
SWH 6393.862 Hz
FIDRES 0.390250 Hz
AQ 2.5624576 sec
RG 105.78
DW 78.200 usec
DE 10.00 usec
TE 298.2 K
D1 0.75000000 sec
TD0 1
SFO1 399.8023988 MHz
NUC1 1H
P1 12.00 usec
PLW1 7.80000019 W
F2 - Processing parameters
SI 65536
SF 399.8000360 MHz
WDW EM
SSB 0
LB 0.39 Hz
GB 0
PC 1.00
7.5 7.0 6.5 6.0 5.5 5.0 4.5 4.0 3.5 3.0 2.5 2.0 1.5 1.0 ppm
54.96
0.83
44.21

Fig AC.

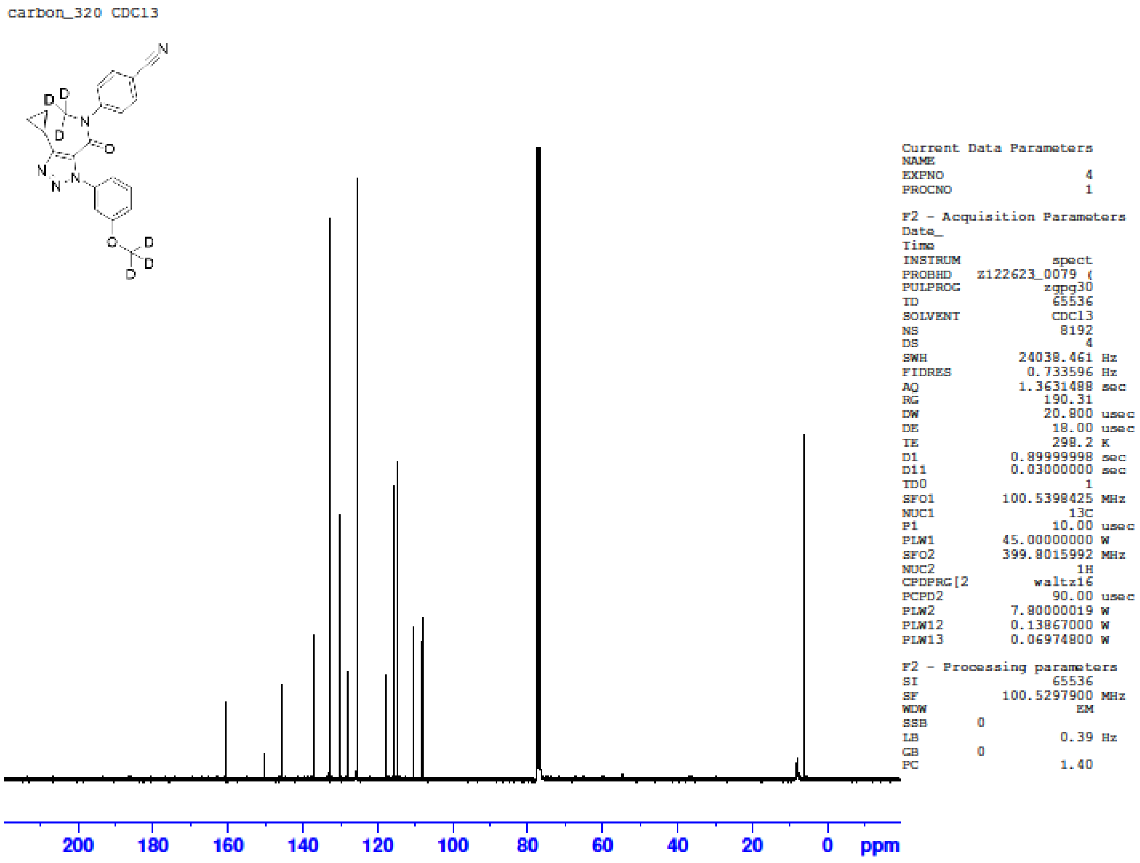

---

[1] Piotrowski DW, Futatsugi K, Warmus JS, Orr STM, Freeman-Cook KD, Londregan AT, et al. Identification of Tetrahydropyrido[4,3-*d*]pyrimidine Amides as a New Class of Orally Bioavailable TGR5 Agonists. ACS Med. Chem. Lett. 2013;4(1): 63–68.

[2] Futatsugi K, Bahnck KB, Brenner MB, Buxton J, Chin JE, Coffey SB, et al. Optimization of triazole-based TGR5 agonists towards orally available agents. Med. Chem. Commun. 2013; 4: 205–210.
