# Supplementary material for: Deuterium isotope effects in drug pharmacokinetics II: Substrate-dependence of the reaction mechanism influences outcome for cytochrome P450 cleared drugs
Source: PLoS One. 2018 Nov 14;13(11):e0206279. doi: 10.1371/journal.pone.0206279 (PMC6235261; doi:10.1371/journal.pone.0206279)
Supplement: S2 File — Figure A. M1a1. UV retention time 33.31 minutes. Figure B. M1a2. UV retention time 34.8 minutes. Figure C. M1a3. UV retention time 34.40 minutes. Figure D. M1a4. UV retention time 35.9 minutes. Figure E. M1a5. UV retention time 36.8 minutes. Figure F. M1a6. UV retention time 37.26 minutes. Figure G. M2a1. UV retention time 43.5 minutes. Figure H. M2a2. Figure I. M2a3. UV retention time 30.83 minutes. (PDF) [file pone.0206279.s002.pdf]

S2 Supporting Information

Deuterium isotope effects in drug pharmacokinetics II: Substrate-dependence of the reaction mechanism influences outcome for cytochrome P450 cleared drugs

Hao Sun,* David W. Piotrowski*, Suvi T. M. Orr, Joseph S. Warmus, Angela C. Wolford, Stephen B. Coffey, Kentaro Futatsugi, Yinsheng Zhang and Alfin D. N. Vaz*

Medicine Design, Pfizer Global Research and Development, Groton, Connecticut, United States of America

Fig A.

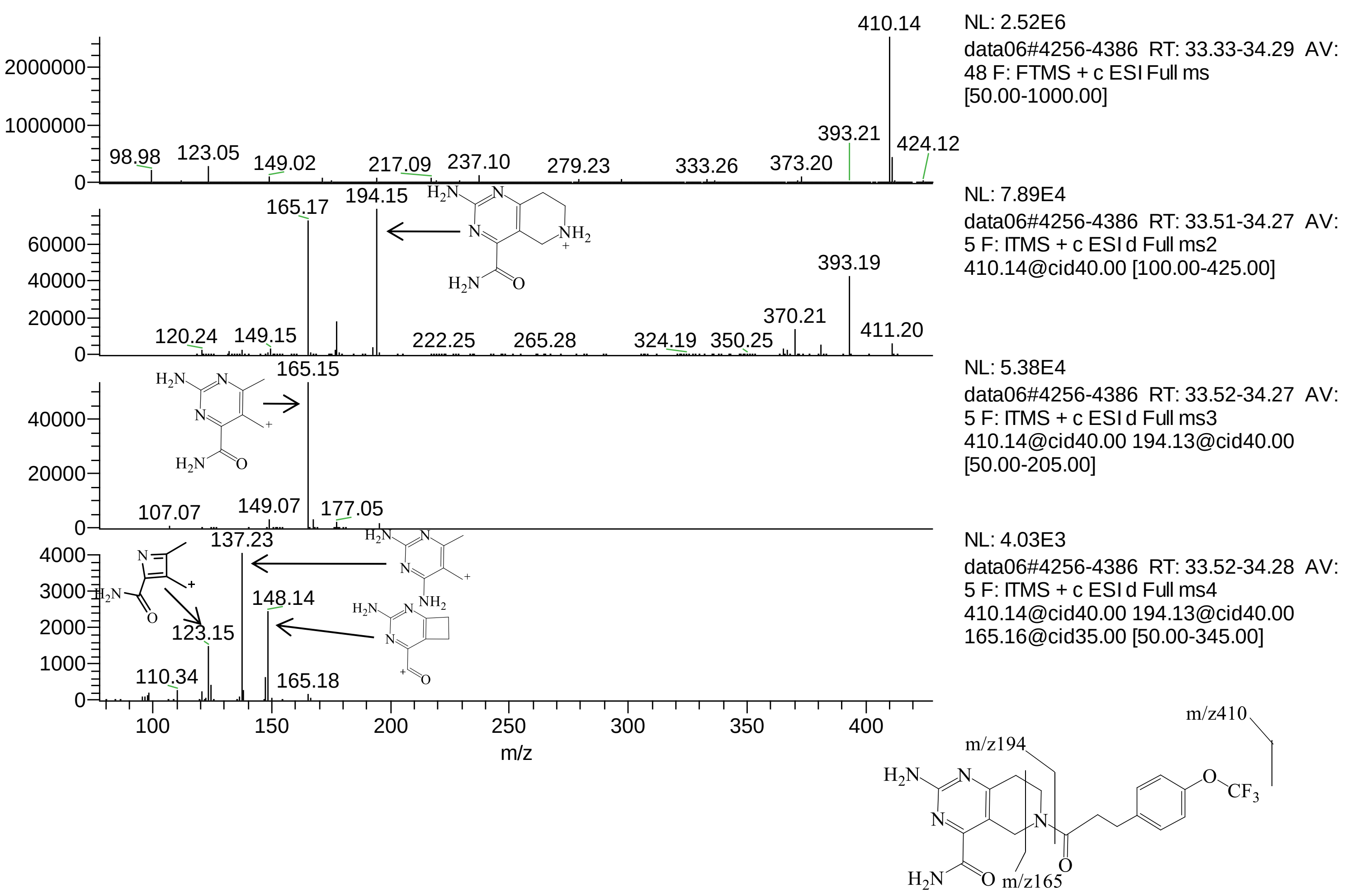
410.14
NL: 2.52E6
data06#4256-4386 RT: 33.33-34.29 AV: 48 F: FTMS + c ESI Full ms [50.00-1000.00]
2000000
1000000
0
98.98
123.05
149.02
217.09
237.10
279.23
333.26
373.20
393.21
424.12
NL: 7.89E4
data06#4256-4386 RT: 33.51-34.27 AV: 5 F: ITMS + c ESI d Full ms2 410.14@cid40.00 [100.00-425.00]
165.17
194.15
60000
40000
20000
393.19
370.21
411.20
120.24
149.15
222.25
265.28
324.19
350.25
NL: 5.38E4
data06#4256-4386 RT: 33.52-34.27 AV: 5 F: ITMS + c ESI d Full ms3 410.14@cid40.00 194.13@cid40.00 [50.00-205.00]
165.15
107.07
149.07
177.05
NL: 4.03E3
data06#4256-4386 RT: 33.52-34.28 AV: 5 F: ITMS + c ESI d Full ms4 410.14@cid40.00 194.13@cid40.00 165.16@cid35.00 [50.00-345.00]
137.23
148.14
123.15
110.34
165.18
4000
3000
2000
1000
100
150
200
250
300
350
400
m/z
m/z410
m/z194
m/z165

Fig B.

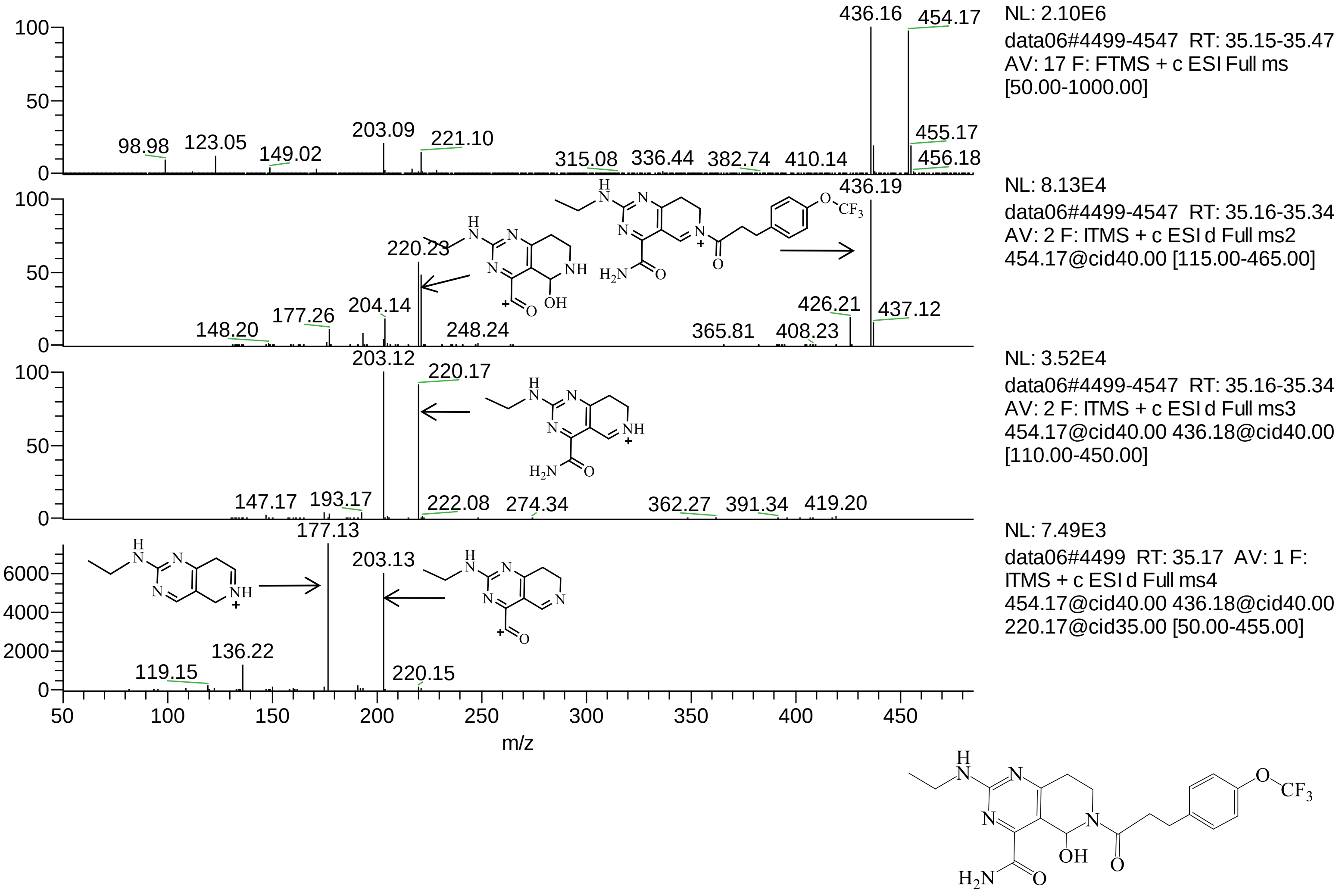
436.16
454.17
NL: 2.10E6
data06#4499-4547 RT: 35.15-35.47
AV: 17 F: FTMS + c ESI Full ms
[50.00-1000.00]
98.98
123.05
149.02
203.09
221.10
315.08
336.44
382.74
410.14
455.17
456.18
436.19
NL: 8.13E4
data06#4499-4547 RT: 35.16-35.34
AV: 2 F: ITMS + c ESI d Full ms2
454.17@cid40.00 [115.00-465.00]
220.23
148.20
177.26
204.14
248.24
365.81
408.23
426.21
437.12
203.12
220.17
NL: 3.52E4
data06#4499-4547 RT: 35.16-35.34
AV: 2 F: ITMS + c ESI d Full ms3
454.17@cid40.00 436.18@cid40.00
[110.00-450.00]
147.17
193.17
222.08
274.34
362.27
391.34
419.20
177.13
NL: 7.49E3
data06#4499 RT: 35.17 AV: 1 F:
ITMS + c ESI d Full ms4
454.17@cid40.00 436.18@cid40.00
220.17@cid35.00 [50.00-455.00]
203.13
136.22
119.15
220.15
m/z

Fig C.

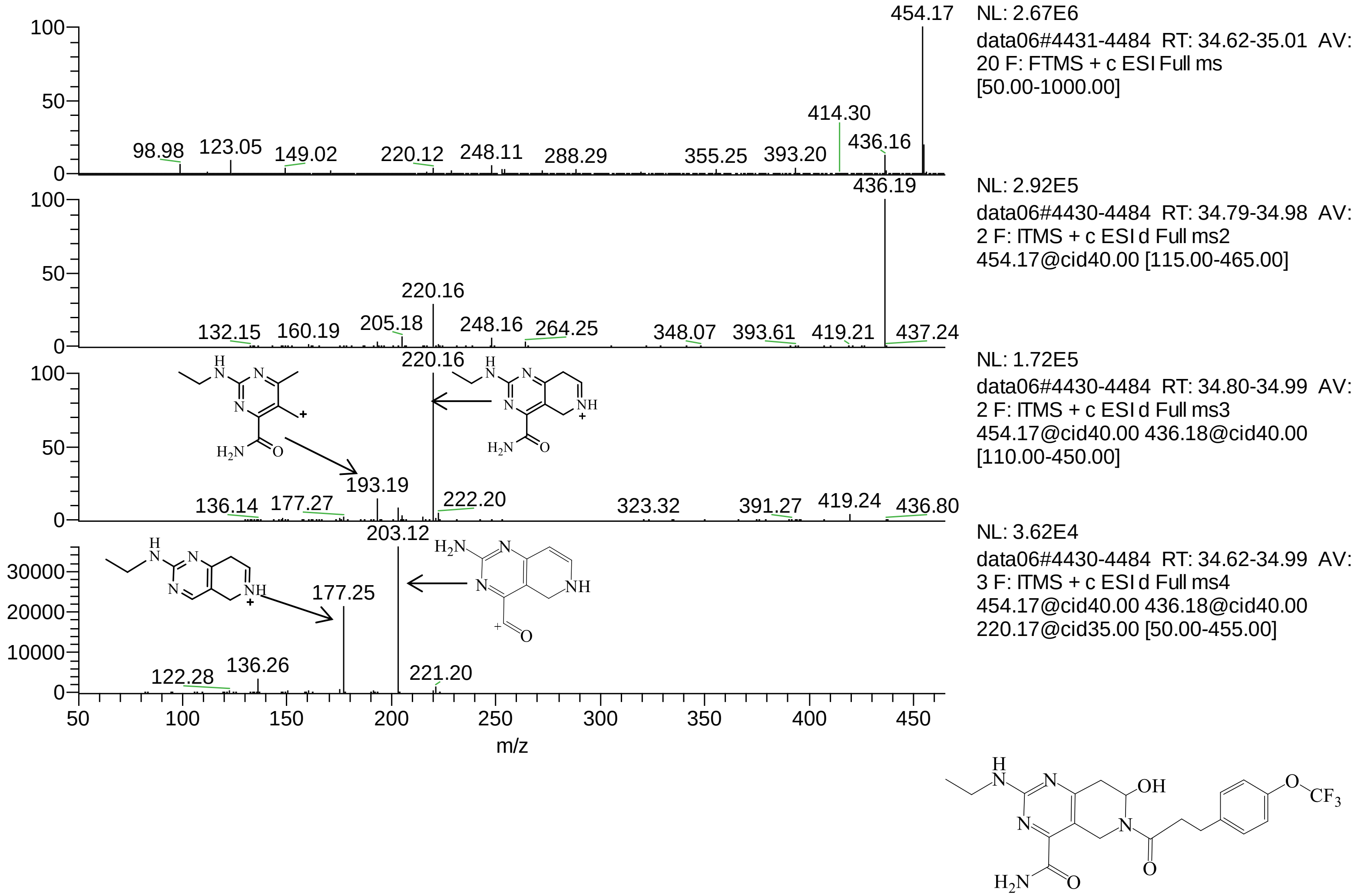

Integration = 0.63 E8 MS; 0.03 E6 UV

Fig D.

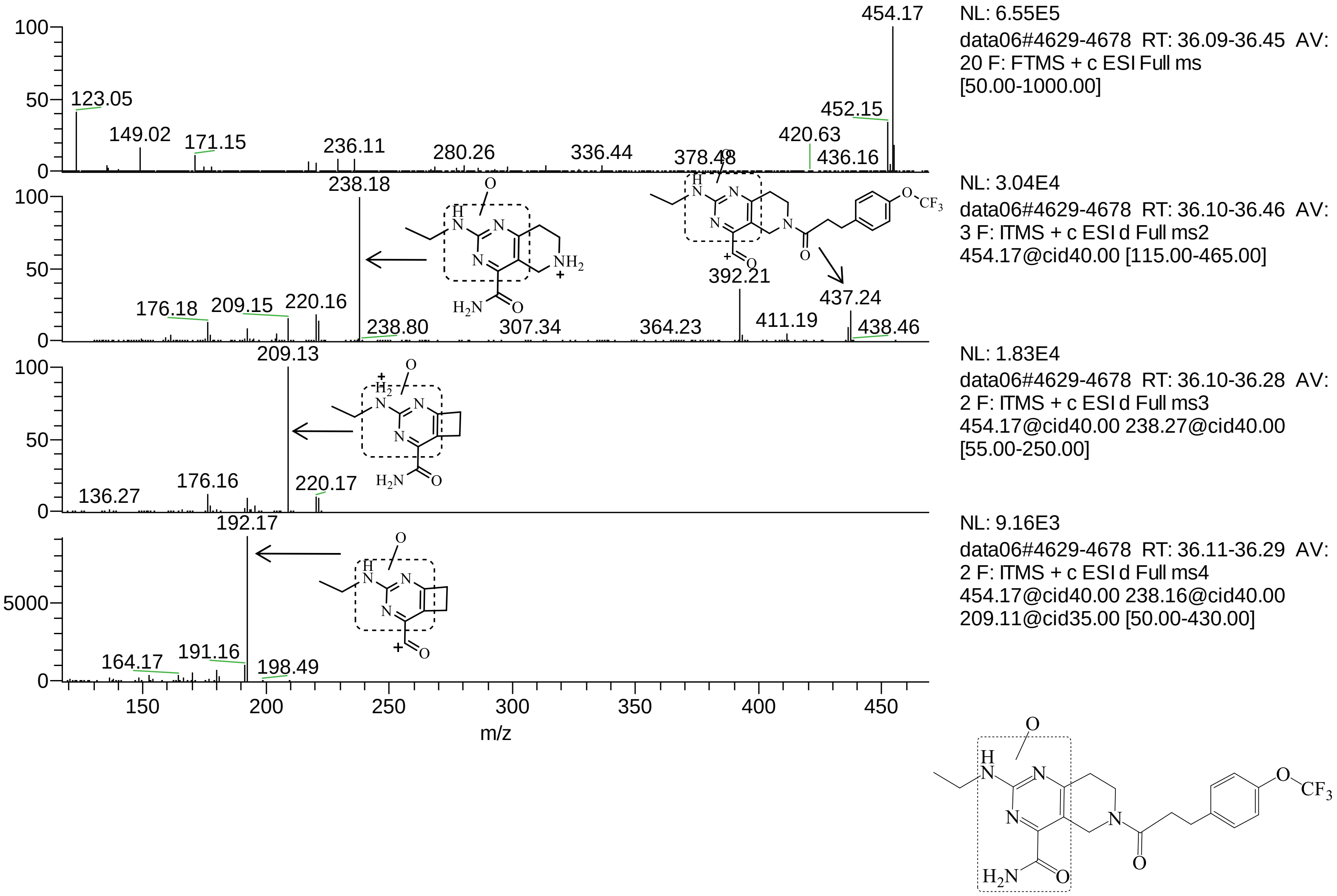
454.17
123.05
149.02
171.15
236.11
280.26
336.44
378.48
420.63
452.15
436.16
NL: 6.55E5
data06#4629-4678 RT: 36.09-36.45 AV: 20 F: FTMS + c ESI Full ms [50.00-1000.00]
238.18
176.18
209.15
220.16
238.80
307.34
364.23
392.21
411.19
437.24
438.46
NL: 3.04E4
data06#4629-4678 RT: 36.10-36.46 AV: 3 F: ITMS + c ESI d Full ms2 454.17@cid40.00 [115.00-465.00]
209.13
136.27
176.16
220.17
NL: 1.83E4
data06#4629-4678 RT: 36.10-36.28 AV: 2 F: ITMS + c ESI d Full ms3 454.17@cid40.00 238.27@cid40.00 [55.00-250.00]
192.17
164.17
191.16
198.49
NL: 9.16E3
data06#4629-4678 RT: 36.11-36.29 AV: 2 F: ITMS + c ESI d Full ms4 454.17@cid40.00 238.16@cid40.00 209.11@cid35.00 [50.00-430.00]
m/z

Fig E.

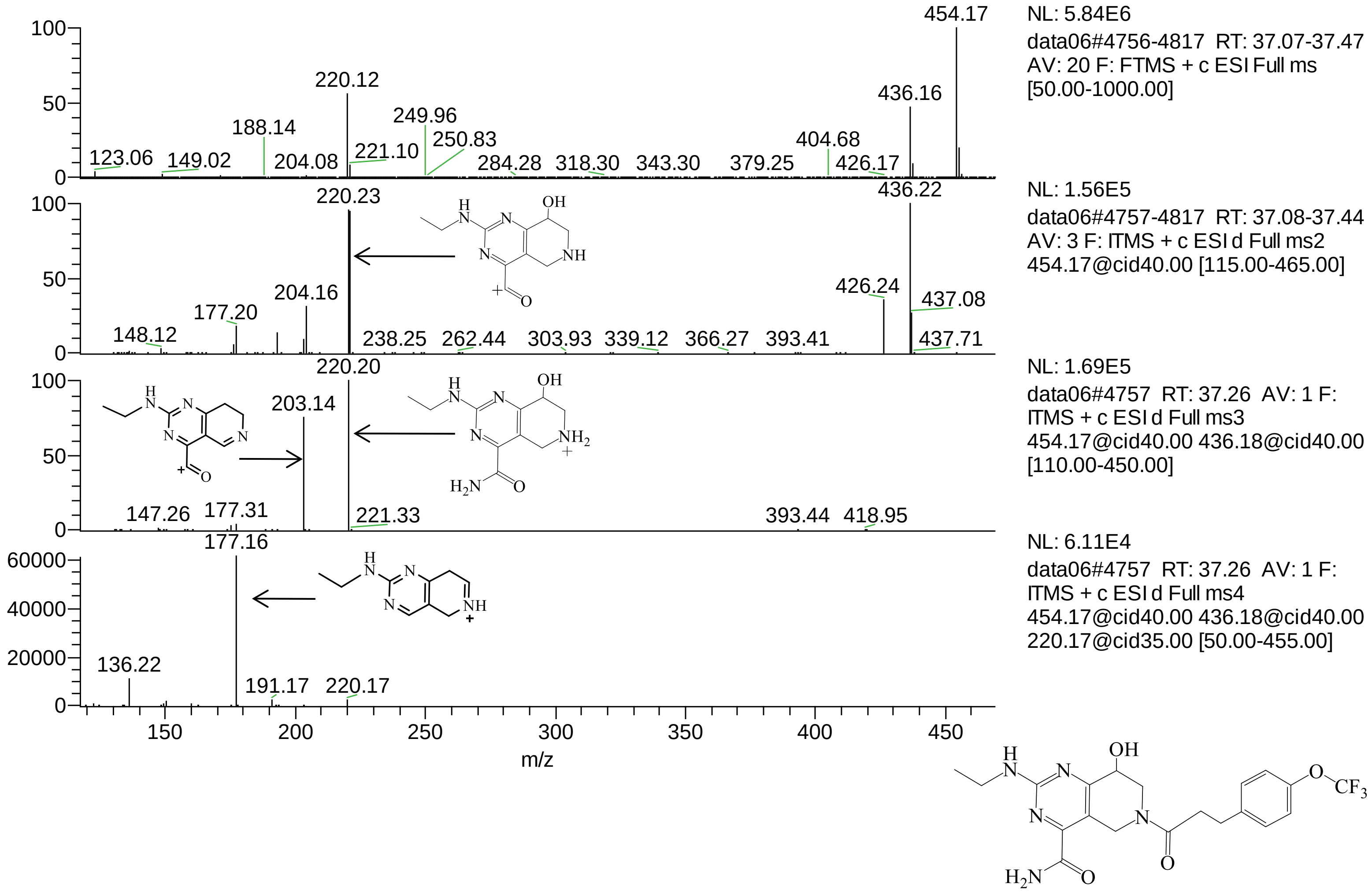
454.17
NL: 5.84E6
data06#4756-4817 RT: 37.07-37.47
AV: 20 F: FTMS + c ESI Full ms
[50.00-1000.00]
220.12
436.16
249.96
188.14
250.83
404.68
123.06
149.02
204.08
221.10
284.28
318.30
343.30
379.25
426.17
220.23
436.22
NL: 1.56E5
data06#4757-4817 RT: 37.08-37.44
AV: 3 F: ITMS + c ESI d Full ms2
454.17@cid40.00 [115.00-465.00]
204.16
426.24
437.08
177.20
148.12
238.25
262.44
303.93
339.12
366.27
393.41
437.71
220.20
NL: 1.69E5
data06#4757 RT: 37.26 AV: 1 F:
ITMS + c ESI d Full ms3
454.17@cid40.00 436.18@cid40.00
[110.00-450.00]
203.14
147.26
177.31
221.33
393.44
418.95
177.16
NL: 6.11E4
data06#4757 RT: 37.26 AV: 1 F:
ITMS + c ESI d Full ms4
454.17@cid40.00 436.18@cid40.00
220.17@cid35.00 [50.00-455.00]
136.22
191.17
220.17
m/z

Fig F.

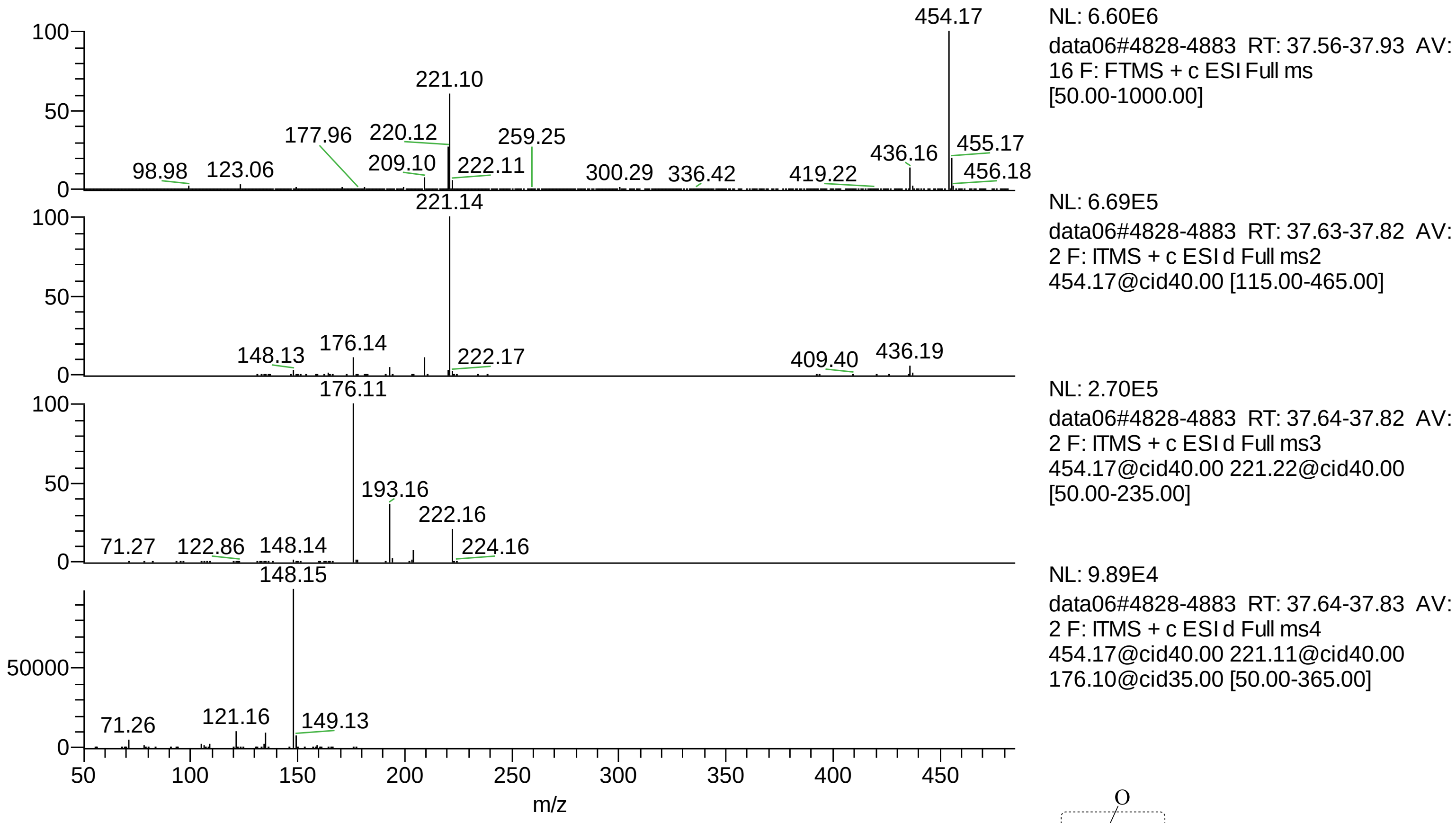
454.17
221.10
177.96
220.12
259.25
455.17
436.16
209.10
222.11
300.29
336.42
419.22
456.18
98.98
123.06
NL: 6.60E6
data06#4828-4883 RT: 37.56-37.93 AV: 16 F: FTMS + c ESI Full ms [50.00-1000.00]
221.14
148.13
176.14
222.17
409.40
436.19
NL: 6.69E5
data06#4828-4883 RT: 37.63-37.82 AV: 2 F: ITMS + c ESI d Full ms2 454.17@cid40.00 [115.00-465.00]
176.11
193.16
222.16
71.27
122.86
148.14
224.16
NL: 2.70E5
data06#4828-4883 RT: 37.64-37.82 AV: 2 F: ITMS + c ESI d Full ms3 454.17@cid40.00 221.22@cid40.00 [50.00-235.00]
148.15
71.26
121.16
149.13
NL: 9.89E4
data06#4828-4883 RT: 37.64-37.83 AV: 2 F: ITMS + c ESI d Full ms4 454.17@cid40.00 221.11@cid40.00 176.10@cid35.00 [50.00-365.00]
100
50
0
50000
50
100
150
200
250
300
350
400
450
m/z

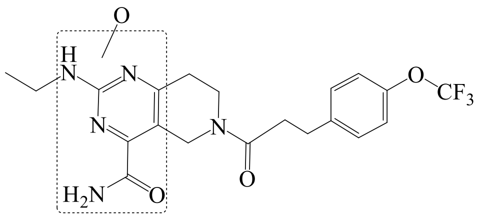
O
H
N
N
N
N
O
CF3
H2N
O
O

Fig G.

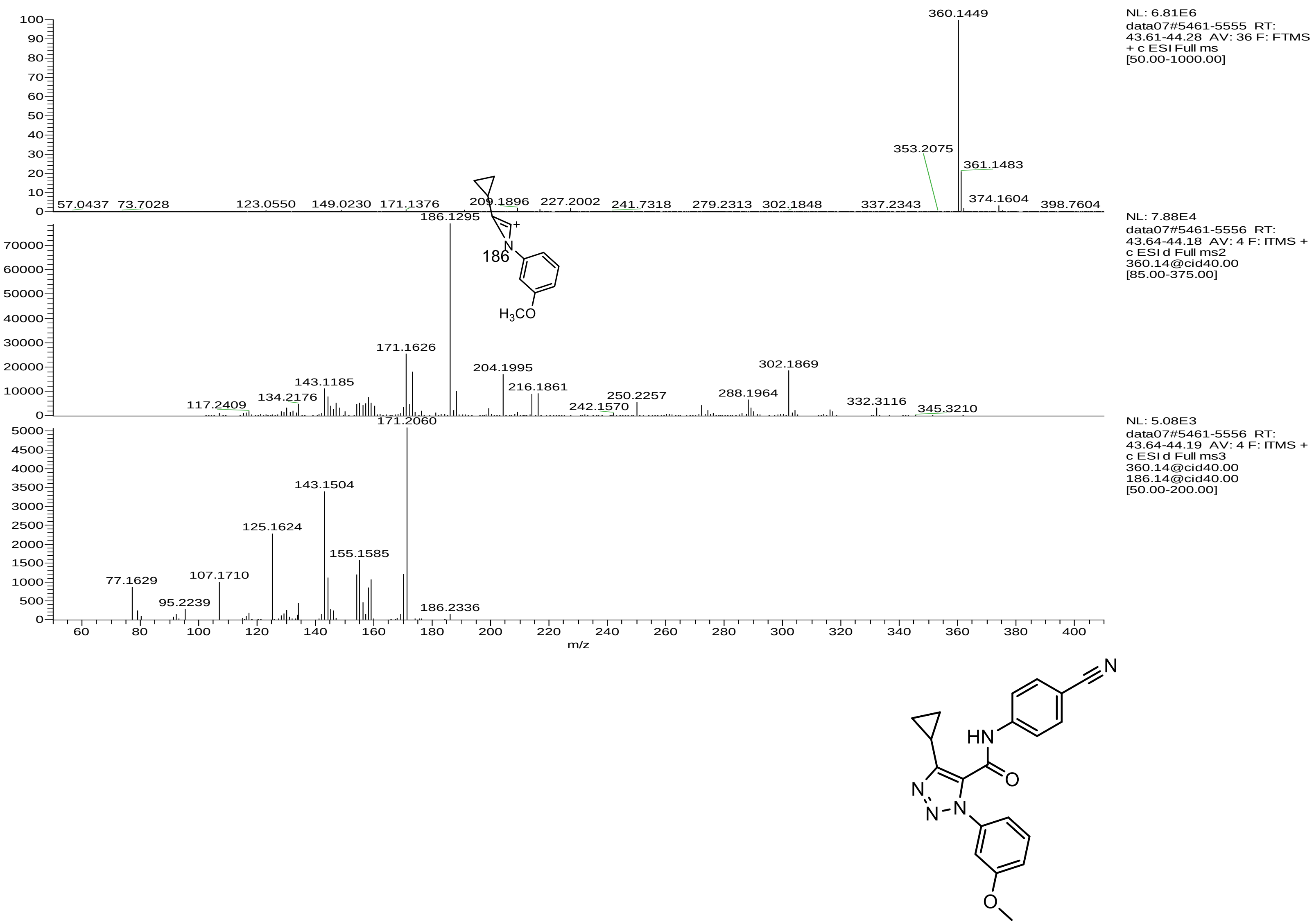
NL: 6.81E6
data07#5461-5555 RT: 43.61-44.28 AV: 36 F: FTMS + c ESI Full ms [50.00-1000.00]
360.1449
353.2075
361.1483
374.1604
398.7604
57.0437
73.7028
123.0550
149.0230
171.1376
209.1896
227.2002
241.7318
279.2313
302.1848
337.2343
NL: 7.88E4
data07#5461-5556 RT: 43.64-44.18 AV: 4 F: ITMS + c ESI d Full ms2 360.14@cid40.00 [85.00-375.00]
186.1295
186
H_3CO
171.1626
204.1995
216.1861
302.1869
143.1185
134.2176
117.2409
242.1570
250.2257
288.1964
332.3116
345.3210
NL: 5.08E3
data07#5461-5556 RT: 43.64-44.19 AV: 4 F: ITMS + c ESI d Full ms3 360.14@cid40.00 186.14@cid40.00 [50.00-200.00]
171.2060
143.1504
125.1624
155.1585
107.1710
77.1629
95.2239
186.2336
m/z
HN
O
N
N
N
N
O

Fig H.

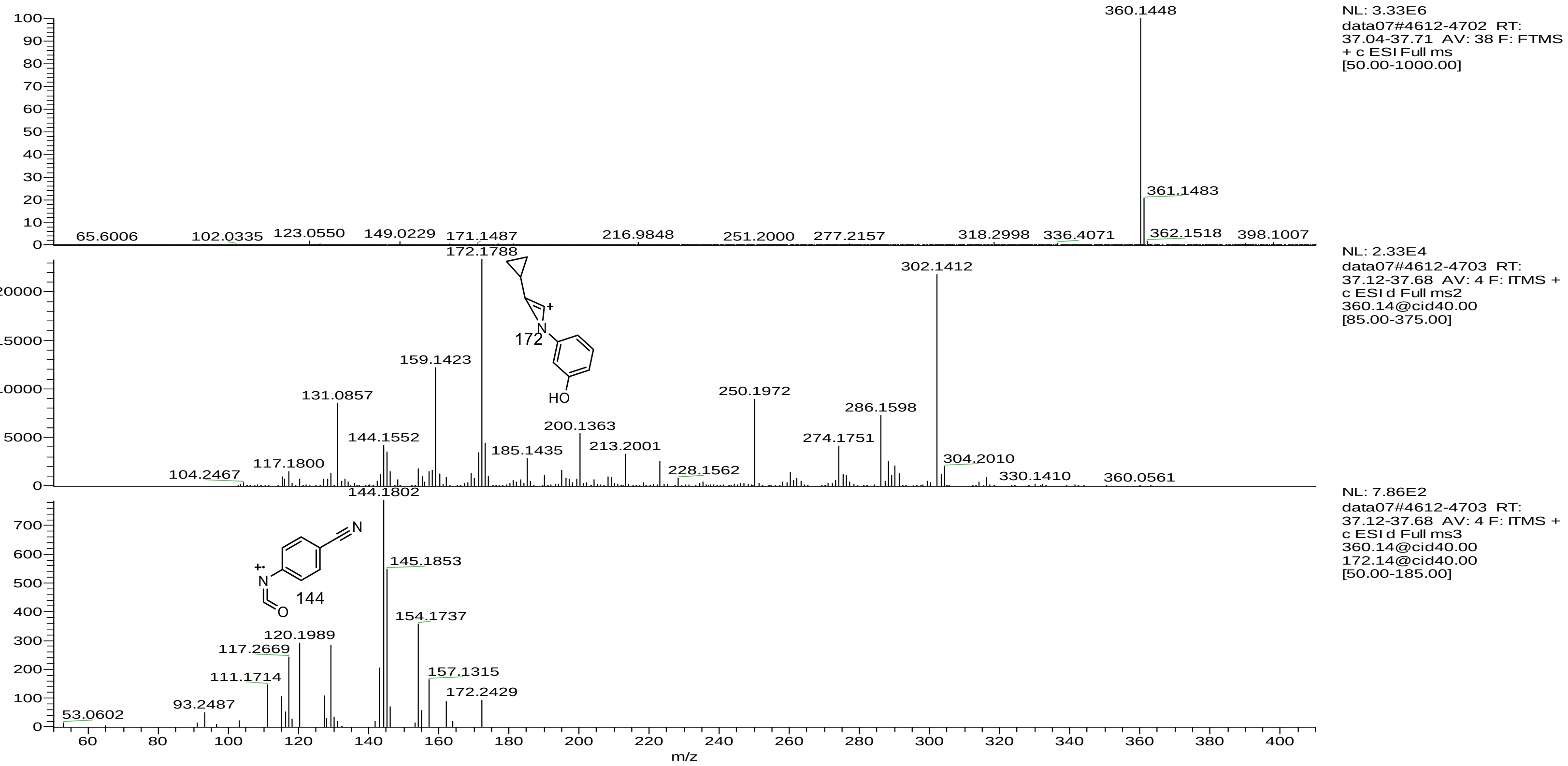
360.1448
361.1483
65.6006
102.0335
123.0550
149.0229
171.1487
216.9848
251.2000
277.2157
318.2998
336.4071
362.1518
398.1007
NL: 3.33E6
data07#4612-4702 RT: 37.04-37.71 AV: 38 F: FTMS + c ESI Full ms [50.00-1000.00]
172.1788
302.1412
172
HO
159.1423
131.0857
250.1972
286.1598
144.1552
200.1363
274.1751
185.1435
213.2001
117.1800
104.2467
228.1562
304.2010
330.1410
360.0561
NL: 2.33E4
data07#4612-4703 RT: 37.12-37.68 AV: 4 F: ITMS + c ESI d Full ms2 360.14@cid40.00 [85.00-375.00]
144.1802
145.1853
144
154.1737
120.1989
117.2669
111.1714
157.1315
172.2429
93.2487
53.0602
NL: 7.86E2
data07#4612-4703 RT: 37.12-37.68 AV: 4 F: ITMS + c ESI d Full ms3 360.14@cid40.00 172.14@cid40.00 [50.00-185.00]
m/z

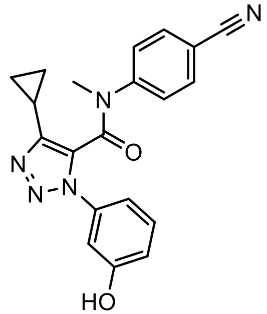
N
N
O
N
N
N
HO

Fig I.

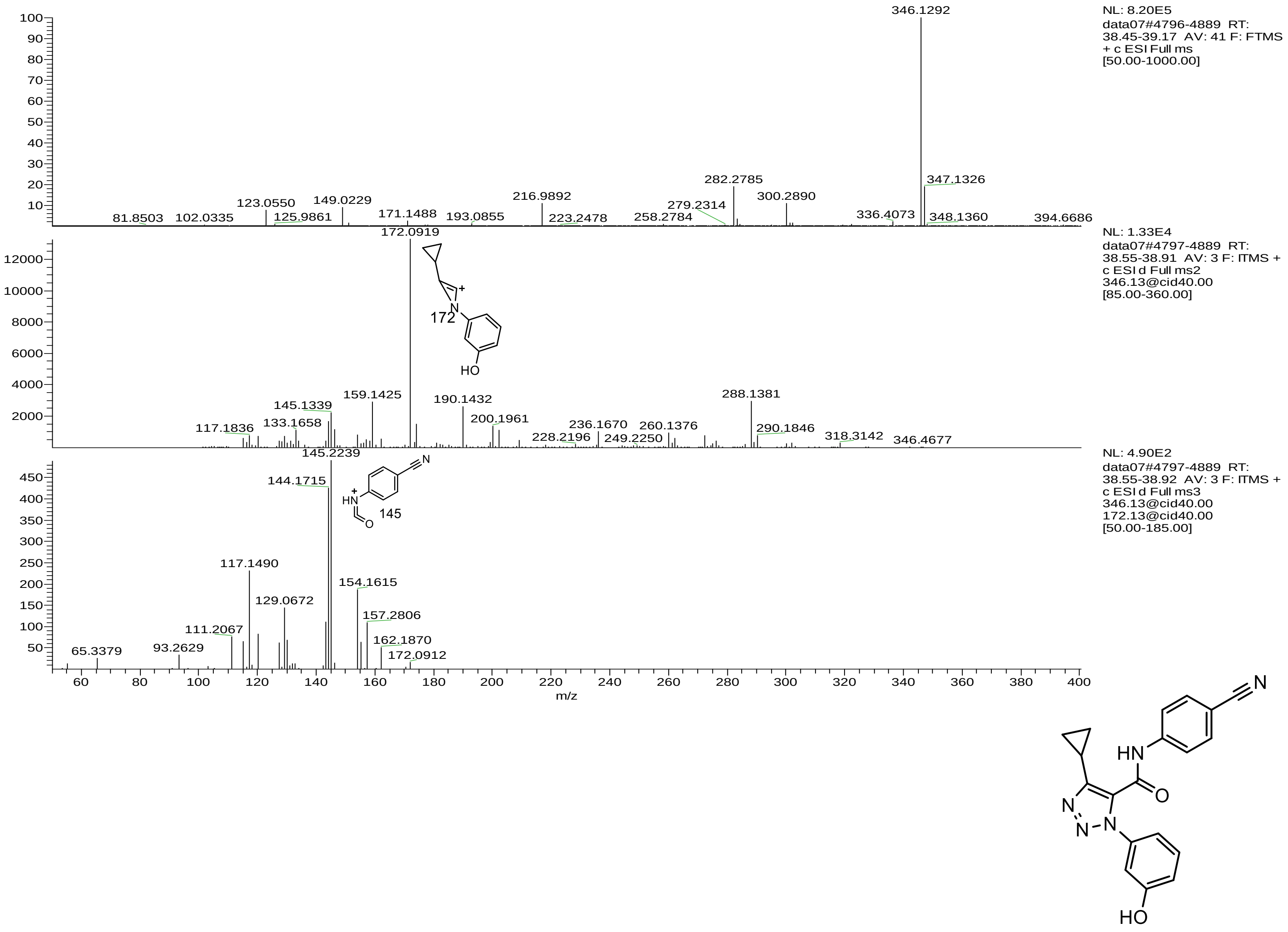
346.1292
NL: 8.20E5
data07#4796-4889 RT: 38.45-39.17 AV: 41 F: FTMS + c ESI Full ms [50.00-1000.00]
81.8503
102.0335
123.0550
125.9861
149.0229
171.1488
193.0855
216.9892
223.2478
258.2784
279.2314
282.2785
300.2890
336.4073
347.1326
348.1360
394.6686
NL: 1.33E4
data07#4797-4889 RT: 38.55-38.91 AV: 3 F: ITMS + c ESI d Full ms2 346.13@cid40.00 [85.00-360.00]
172.0919
172
HO
117.1836
133.1658
145.1339
159.1425
190.1432
200.1961
228.2196
236.1670
249.2250
260.1376
288.1381
290.1846
318.3142
346.4677
NL: 4.90E2
data07#4797-4889 RT: 38.55-38.92 AV: 3 F: ITMS + c ESI d Full ms3 346.13@cid40.00 172.13@cid40.00 [50.00-185.00]
145.2239
144.1715
HN
O
N
145
117.1490
129.0672
154.1615
157.2806
162.1870
172.0912
111.2067
93.2629
65.3379
m/z
HN
O
N
HO
